# Targeting SRSF1 improves cancer immunotherapy by dually acting on CD8^+^T and tumor cells

**DOI:** 10.1038/s41392-024-02118-2

**Published:** 2025-01-22

**Authors:** Gui-Qi Zhu, Zheng Tang, Tian-Hao Chu, Biao Wang, Shi-Ping Chen, Chen-Yang Tao, Jia-Liang Cai, Rui Yang, Wei-Feng Qu, Yi Wang, Qian-Fu Zhao, Run Huang, Meng-Xin Tian, Yuan Fang, Jun Gao, Xiao-Ling Wu, Jian Zhou, Wei-Ren Liu, Zhi Dai, Ying-Hong Shi, Jia Fan

**Affiliations:** 1 Department of Liver Surgery and Transplantation, Liver Cancer Institute, Zhongshan Hospital, Fudan University; Key Laboratory of Carcinogenesis and Cancer Invasion of Ministry of Education, Shanghai, China; 2 Research Unit of Liver cancer Recurrence and Metastasis, Chinese Academy of Medical Sciences, Beijing, China; 3 Department of Radiation Oncology, Zhongshan Hospital, Fudan University, Shanghai, China; 4 Department of General Surgery, Gastric cancer center, Zhongshan Hospital, Fudan University, 200032 Shanghai, China

**Keywords:** Drug development, Drug screening

## Abstract

Serine arginine-rich splicing factor 1 (SRSF1) is a key oncogenic splicing factor in various cancers, promoting abnormal gene expression through post-translational regulation. Although the protumoral function of SRSF1 is well-established, the effects of inhibiting tumor-intrinsic SRSF1 on the tumor microenvironment and its impact on CD8^+^ T cell-mediated antitumor immunity remain unclear. Our findings indicate that depleting SRSF1 in CD8^+^ T cells improve antitumor immune function, glycolytic metabolism, and the efficacy of adoptive T cell therapy. The inactivation of SRSF1 in tumor cells reduces transcription factors, including c-Jun, c-myc, and JunB, facilitating glycolytic metabolism reprogramming, which restores CD8^+^ T cell function and inhibits tumor growth. The small-molecule inhibitor TN2008 targets SRSF1, boosting antitumor immune responses and improving immunotherapy effectiveness in mouse models. We therefore introduce a paradigm targeting SRSF1 that simultaneously disrupts tumor cell metabolism and enhances the antitumor immunity of CD8^+^ T cells.

## Introduction

Globally, hepatocellular carcinoma (HCC) is the fourth most frequent cause of death due to cancer, and the main risk factor is chronic infection with the hepatitis B (HBV) virus.^1^ Patients with unresectable HCC still have a poor prognosis, limited therapy options, and a high recurrence rate following surgical resection.^1,2^ Recently, immune checkpoint blockade (ICB) therapy has showed its astonishing effects in reducing tumor growth for many cancer patients.^2,3^ While immune checkpoint blockade (ICB) has transformed HCC treatment, it elicits a lasting clinical response in only a minority of patients.^4,5^ It has been considered that the challenges such as low response rate and acquired resistance still exist, which mainly because tumor-intrinsic pathways activation that promote immune escape or exhaustion of cytotoxic T cell.^6^ Despite the development of numerous cancer drugs targeting essential signaling pathways to significantly reduce tumors, recurrence remains a significant issue due to the emergence of drug-resistant tumor cells. While the immune system may target residual disease, numerous tumor-targeting drugs also impair immune cell survival and function.^7^ Therefore, developing tumor-targeted drugs that boost immune function is crucial.

Suppression of endogenous anti-tumor immune responses is often necessary for the development and spread of malignancies.^7^ T cell failure within tumors frequently happens despite their capacity to recognize cancer-specific antigens, because of a collection of functional deficiencies termed T cell ‘exhaustion’.^7^ A diminished capacity to proliferate, decreased synthesis of cytotoxic effector molecules, and increased expression of inhibitory immunoreceptors, including cytotoxic T-lymphocyte-associated protein 4 (CTLA-4) and Programmed Death-1 (PD-1), are characteristics of T cell exhaustion.^7,8^ Enhancing the function of T cells with immune checkpoint blockades or by expressing chimeric antigen receptors (CARs) has shown potential in cancer treatment^8^; Regrettably, the majority of patients do not achieve a lasting response to immune-based therapy. The transportation of T cells to the tumor and the resolution of T cell exhaustion in the tumor microenvironment (TME) have been identified as two important limiting factors in the cancer immunity cycle.^8^ Recent investigations have stressed the role of tumor and T cell metabolism in the development of cancer and the resistance to immunotherapy.^9^ Glucose deprivation and hypoxia in the TME impose metabolic restrictions that substantially affect the signaling pathways of tumor-infiltrating lymphocytes, leading to reduced anti-tumor immune activity.^8,9^ Research suggests that T cells experiencing exhaustion have lower levels of glycolysis and/or mitochondrial respiration, possibly increasing their metabolic inefficiency and exhaustion.^7–9^ Hence, there is an active pursuit of metabolic interventions aimed at enhancing the effector capabilities and proliferation of T cells.

Evolutionarily conserved, the serine/arginine-rich (SR) proteins are important for constitutive and alternative splicing.^10^ Serine arginine-rich splicing factor 1 (SRSF1), which is overexpressed in many malignancies and can encourage transformation, is one of several SR proteins that have oncogenic qualities across various cancer types.^10–12^ It is commonly recognized that the typical member of the conserved SR family of RNA-binding proteins is SRSF1, formerly known as splicing factor 2/alternative splicing factor (SF2/ASF).^11^ Through its effects on mRNA stability and pre-mRNA alternative splicing, SRSF1 controls posttranscriptional gene expression.^11^ According to one previous investigation, SRSF1 suppressed autophagy in lung cancer by controlling Bcl-x splicing and interacting with PIK3C3.^12^ Furthermore, when KRASG12D is expressed, the decrease of SRSF1 protein contributes to a negative feedback biological process that helps maintain pancreatic cell homeostasis.^10^ In terms of its role in T cells, research also indicates that SRSF1 supports normal CD3ζ expression by restricting an unstable 3ʹ UTR isoform,^13,14^ and its levels decline during T cell activation.^15^ Although the role of SRSF1 in CD4^+^T cells has been elucidated in autoimmunity disease, the function of SRSF1 in CD8^+^T cells and its impact on antitumor immunity remains unexplored. Several investigations, ours among them, propose that SRSF1 may facilitate the growth of cancer cells via diverse tumor-intrinsic processes, thus serving as a potential therapeutic target in solid tumors.^11,14,16–18^ It is believed that SRSF1 contributes to tumor formation in breast, lung, and colon cancers, where its levels are increased. The cell-autonomous roles of SRSF1 in cancer carcinogenesis have been the focus of these investigations. However, the impact of SRSF1 on tumor growth through non-cell or cell-autonomous effects on CD8^+^ T cell function is not yet fully understood.

Our findings indicate that knocking out SRSF1 in CD8^+^ T cells enhances both CD38 expression and the cytotoxicity of these cells in vitro. SRSF1 depletion suppresses glycolytic genes in tumor cells by limiting bZIP and MYC transcription factors, thereby eliminating the metabolic barrier to T cell activation. Our study revealed that SRSF1 plays a dual role in tumorigenesis within tumors and T cells during immune escape. Targeting its abnormal activity with the novel inhibitor TN2008 improved T cell functionality, boosting antitumor immune responses and improving immunotherapy effectiveness in mouse models, especially when combined with anti-PD-1 immunotherapy, showed enhanced effects in resistant cancer models.

## Results

### SRSF1 levels are elevated in immunotherapy non-responsive CD8^+^ T cells

From the TCGA database and GTEx database, we found that SRSF1 mRNA expression was significantly upregulated in 15 of 31 types of tumor tissues (Supplementary Fig. 1a, b). The application of the ESTIMATE algorithm revealed a positive correlation between SRSF1 mRNA expression and exhausted CD8^+^ T cells across various cancers (Supplementary Fig. 1c), but negative correlation can be seen between SRSF1 mRNA and cytotoxic or memory T cells (Supplementary Fig. 1d). Also, we observed a strong positive correlation between SRSF1 expression and T cell exhaustion signatures (Supplementary Fig. 1e). Analysis of public scRNA-seq data revealed elevated SRSF1 expression in CD8^+^ T cells from tumors compared to adjacent tissues in cases of CRC or RCC (Supplementary Fig. 1f, g).

**Fig. 1:**
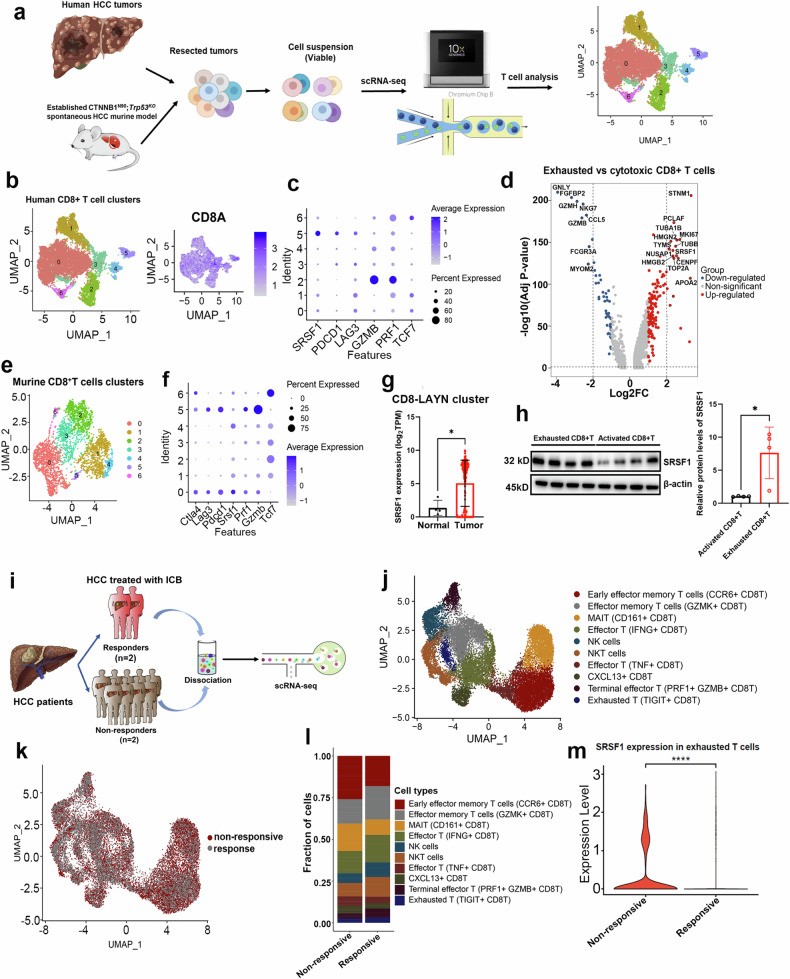
SRSF1 levels are associated with exhausted phenotypes in CD8^+^T cells. **a** Diagram illustrating the workflow of single-cell RNA sequencing (scRNA-seq) analysis. The figure was obtained by using Biorender (https://app.biorender.com/). **b** UMAP visualizations depicting various clusters within human CD8^+^ T cells. Clusters 0, 1, and 6 represent naive CD8^+^ T cells; Cluster 2 corresponds to effector CD8^+^ T cells; Clusters 3, 4, and 5 consist of exhausted CD8 + T cells. **c** Dotplots illustrate the expression levels of SRSF1 and T cell marker genes across various cell clusters. **d** Scatter plots illustrate differentially expressed genes (DEGs) between cytotoxic and exhausted CD8^+^ T cells. **e** UMAP plots for the cell clusters in murine CD8 + T cells. Cluster 0 consists of exhausted CD8^+^ T cells; Clusters 1, 2, 3, 4, and 6 comprise naive CD8^+^ T cells; Cluster 5 contains effector CD8 + T cells. **f** Dotplots showing marker genes for distinct cell clusters. **g** Expression of SRSF1 in exhausted CD8^+^ T cells from both tumor and normal tissues. **h** Western blot showing SRSF1 expressed more in exhausted CD8^+^T cells than activated CD8^+^T cells. **i** Schematic diagram of scRNA-seq analysis flow for HCC patients undergoing neoadjuvant anti-PD-1 therapy. The figure was obtained by using Biorender (https://app.biorender.com/). **j** UMAP visualizations for various clusters in human CD8^+^ T cells. **k** UMAP plots illustrate cluster variations between anti-PD-1 non-responsive and responsive groups. **l** Barplots showing different CD8^+^T cell clusters in different anti-PD-1 nonresponsive and responsive groups. **m** Comparison of SRSF1 expression in exhausted CD8^+^T cells between nonresponsive and responsive groups. Data presented as mean ± S.E.M. Statistical significance was determined by Wilcoxon signed rank test and two-tailed unpaired *t* test. The *p* value is 0.05, the *p* value is 0.0001

We examined SRSF1 expression in paracancerous and intratumoral CD8^+^ T cells to investigate its role in tumorigenesis. We conducted scRNA-seq experiments to isolate single cells from 7 human HCC samples (Supplementary Table 1) and 5 autochthonous murine HCC tissues created via hydrodynamic tail vein injection of oncogenic plasmids (Fig. 1a), including *CTNNB1*^*N90*^ and *Trp53*^*KO*^, which were the most mutation genes in TCGA HCC databases^19^ (Supplementary Fig. 1h). Our study observed increased SRSF1 levels in intratumoral human and murine CD8^+^ T cells exhibiting an exhausted phenotype, compared to those with a cytotoxic phenotype (Fig. 1b–f). Moreover, SRSF1 levels were elevated in intratumoral exhausted CD8^+^ T cells (high for LAYN) by an analysis of scRNA-seq data (Fig. 1g). Consistently, SRSF1 protein was elevated more than in tumoral exhausted CD8^+^ T cells when compared with cytotoxic CD8^+^ T cells from mice (Fig. 1h). To investigate the mechanisms regulating SRSF1 expression in exhausted CD8^+^ T cells, we conducted a ChIP assay that demonstrated NFATC2, a transcription factor associated with T cell exhaustion,^20,21^ significantly upregulates SRSF1 expression in these cells (Supplementary Fig. 1i).

We conducted scRNA-seq on four HCC tissues—two responsive and two non-responsive—to investigate SRSF1 expression in CD8^+^ T cells during neoadjuvant anti-PD-1 cancer immunotherapy (Supplementary Table 2; Fig. 1i). CD8^+^ T cells were categorized into distinct clusters, demonstrating significant heterogeneity between the two groups (Fig. 1j–l). Interestingly, SRSF1 expression was significantly increased in non-responsive HCC tissues among exhausted samples (Fig. 1m) or pan-CD8 + T cells (Supplementary Table 3). Similar results also can be validated in basal cell carcinoma from public databases (Supplementary Fig. 1j, k). Elevated SRSF1 levels in immunotherapy non-responsive CD8^+^ T cells, particularly in exhausted phenotypes, may negatively impact T-cell and immunotherapy responses.

### SRSF1 depletion in CD8^+^T cells enhance its cytotoxic phenotype and adoptive T cell therapy

**Fig. 2:**
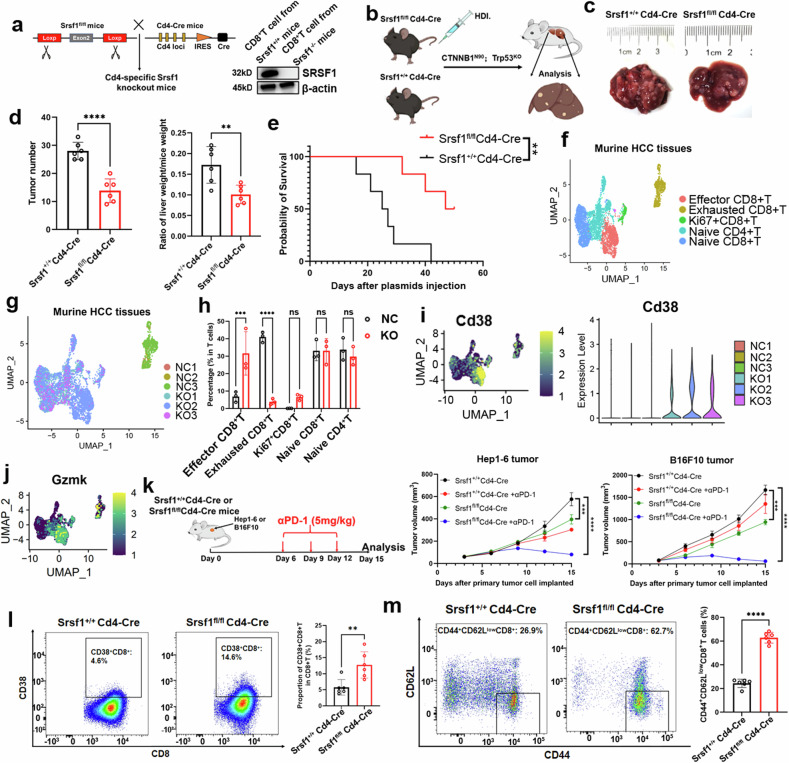
SRSF1 depletion in CD8 + T cells enhance its cytotoxicity. **a** The schematic diagram of establishment of Srsf1 conditional knockout in CD8^+^T cell and Srsf1 expression between two groups from CD8 + T cells. **b**, **c** Comparison of HCC progression between Srsf1^fl/fl^Cd4-cre and Srsf1^+/+^Cd4-cre mice, with *n* = 6 for each group. **d** The study examined the tumor count (left panel) and the liver-to-body weight ratio (right panel) in Srsf1^fl/fl^Cd4-cre and Srsf1^+/+^Cd4-cre mouse groups, with each group consisting of six mice. **e** Kaplan-Meier survival analysis for Srsf1^fl/fl^Cd4-cre and Srsf1^+/+^Cd4-cre mice (*n* = 6 per group). **f**,**g** The UMAP of T cells from murine HCC tissues. NC: Srsf1^+/+^Cd4-cre mice, KO: Srsf1^fl/fl^Cd4-cre. **h** The T cell subset proportions in Srsf1^fl/fl^Cd4-cre and Srsf1^+/+^Cd4-cre mice (*n* = 3 per group) were analyzed. **i** Analysis of scRNA-seq data reveals CD38 expression and distribution differences between Srsf1^fl/fl^Cd4-cre and Srsf1^+/+^Cd4-cre mice. **j** GZMK expression in UMAP plot from scRNA-seq analysis. **k** The schematic diagram for HCC or B16F10 tumor model combined with PD-1 therapy (Hep1-6 or B16F10 cells: 2 × 10^5) and SRSF1 knockout in CD8^+^ T cell synergized with PD-1 therapy in tumor models. **l** Flow cytometry assays revealed a higher increase of CD38 + CD8 + T cells in Srsf1^fl/fl^Cd4-cre mice compared to Srsf1^+/+^Cd4-cre mice. **m** Flow cytometry assays indicated a higher increase of CD44^+^CD62L^low^CD8 + T cells in Srsf1^fl/fl^Cd4-cre mice compared to Srsf1^+/+^Cd4-cre mice. Data presented as mean ± S.E.M. Statistical significance was determined by log rank test, two-way ANOVA, and two-tailed unpaired *t* test. **p < 0.01, ***p < 0.001, ****p < 0.0001, ns indicates no significance

To investigate Srsf1’s role in CD8^+^T cell immune regulation, we bred Srsf1^fl/fl^ mice with CD4-Cre mice, creating mice with Srsf1 deficiency in both CD4^+^ and CD8^+^ T cells. The mice, designated as Srsf1^fl/fl^Cd4-Cre, exhibit complete depletion of Srsf1 in CD8^+^ T cells (Fig. 2a) and the mice body or spleen between these two groups show no significant differences (Supplementary Fig. 2a). CD8^+^T cells from Srsf1^fl/fl^Cd4-Cre mice are designated as Srsf1^–/–^, while those from Srsf1^+/+^Cd4-Cre mice are labeled as Srsf1^+/+^. Further T cell subpopulation explorations showed the proportion of activated CD4^+^T cells (Supplementary Fig. S2b) level was significantly elevated in the Srsf1^fl/fl^; Cd4^cre^ mice compared to the control group.

We then investigated the effects of Srsf1 on T cells under pathological stress by subjecting Srsf1^fl/fl^Cd4-Cre and Srsf1^+/+^Cd4-Cre mice to a *CTNNB1*^*N90*^*; Trp53*^*KO*^-induced autochthonous HCC model (Fig. 2b). We found that Srsf1^fl/fl^Cd4-Cre and Srsf1^fl/fl^Cd8-Cre mice were more efficient than littermate control mice at reducing tumor growth in *CTNNB1*^*N90*^*; Trp53*^*KO*^ murine HCC model (Fig. 2c–e, Supplementary Fig. 2c, d). The scRNA-seq analysis showed that tumors from Srsf1^fl/fl^Cd4-Cre mice group harbored more effector CD8^+^T cells (Fig. 2f–h). Interestingly, Cd38 expression was notably elevated in effector CD8^+^ T cells from the Srsf1^–/–^ mice group (Fig. 2f–i) and CD38^+^CD8^+^T cells also conferred more cytotoxic effects (Fig. 2j), which was previously reported to be an activated phenotype.^22,23^ Recent studies indicate that tumor-infiltrating CD38^+^CD8^+^ T cells respond more effectively to anti-PD-1 therapy.^23^ This prompted us to investigate whether SRSF1 knockdown in T cells could enhance immunotherapy by increasing the infiltration of effector CD38^+^CD8^+^ T cells. Our in vivo data revealed that Srsf1 knockdown had a combinatory effect with anti-PD1 against tumor growth in Hepa1-6 and B16F10 subcutaneous tumor models (Fig. 2k). Flow cytometry assays revealed a significant increase in CD38^+^CD8^+^T cells and effector CD8^+^T cells (CD44^+^CD62L^low^CD8^+^T) in tumors from Srsf1^fl/fl^Cd4-Cre mice compared to Srsf1^+/+^Cd4-Cre mice (Fig. 2l, m).

We investigated if deleting SRSF1 enhances the tumor-specific activity of transgenic OT-I CD8 + T cells transferred into mice with OVA-expressing tumors. Naive (Srsf1^fl/fl^) or Srsf1-deficient (Cd4-Cre; Srsf1^fl/fl^) congenitally marked (Ly5.2 + ) OT-I CD8 + T cells were transferred into immunocompetent, nonirradiated (Ly5.1 + ) C57BL/6 hosts with Hep1-6-OVA HCC tumors (Supplementary Fig. 2e). Adoptively transferred control CD8^+^ T cells showed minimal impact on tumor progression (Supplementary Fig. 2f). Srsf1-deficient CD8^+^ T cells markedly inhibited the growth of Hep1-6-OVA HCC tumors (Supplementary Fig. 2f) and enhanced mouse survival (Supplementary Fig. 2f). Tumor growth suppression was linked to a higher presence of Srsf1-deficient tumor-infiltrating effector T cells (Supplementary Fig. 2g–i); Thus, Srsf1 negatively regulates CD8^+^ T cell functional potency in cancer. Thus, the data suggests lower expression of Srsf1 in CD8^+^T cells enhance its cytotoxic phenotype and adoptive T cell therapy, during antitumor response.

### SRSF1 depletion in CD8^+^ T cells increased CD38 expression and glycolytic metabolism

**Fig. 3:**
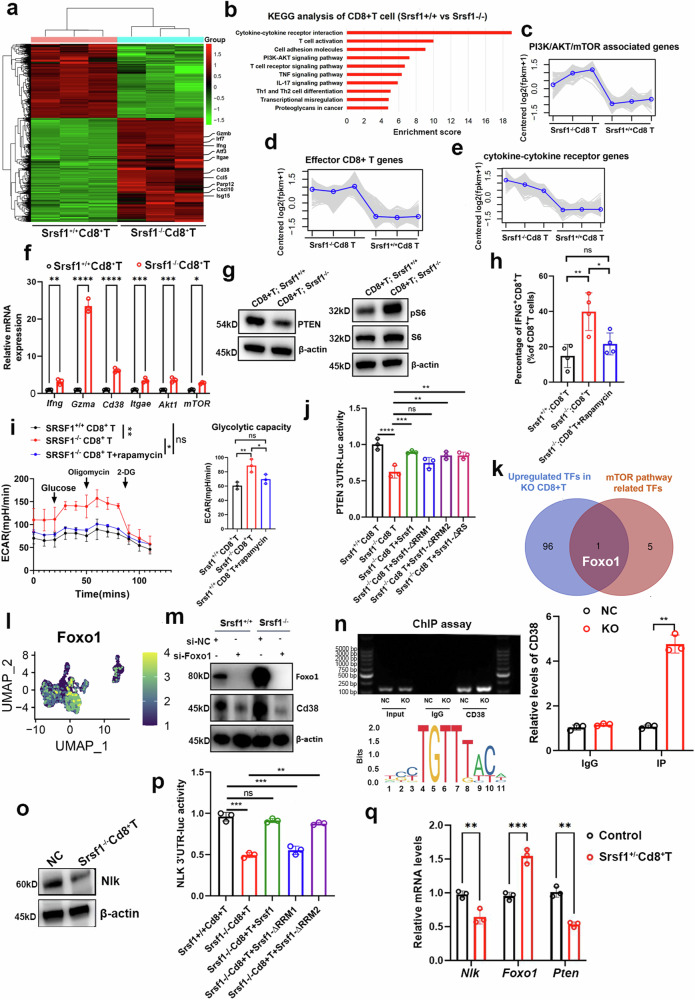
SRSF1 depletion in CD8 + T cells increased CD38 expression by regulating FOXO1. **a** Heatmap showed upregulated or downregulated DEGs in Srsf1^+/+^Cd8+T and Srsf1^–/–^Cd8+T groups. **b** KEGG analysis of CD8 + T cell (Srsf1^+/+^Cd8 T vs Srsf1^–/–^Cd8 T). **c**–**e** Gene cluster analysis of CD8 + T cell (Srsf1^+/+^Cd8 T vs Srsf1^–/–^Cd8 T). **f** qPCR analysis of cytotoxic genes between Srsf1^+/+^Cd8+T and Srsf1^–/–^Cd8+T. **g** Western blot showed PTEN and S6 expression in Srsf1^–/–^Cd8+T and Srsf1^+/+^Cd8+T. **h** The proportion of effector CD8 + T cell in the Srsf1^–/–^Cd8+T, Srsf1^+/+^Cd8+T and Srsf1^–/–^Cd8+T with rapamycin groups. **i** The Seahorse assays showed Cd8+T cells depleting Srsf1 could increase glycolytic metabolism and can be rescued by mTOR inhibitor. **j** Luciferase analysis showed SRSF1-RRM1 was binding to 3ʹUTR of PTEN mRNA. **k** The intersect result between upregulated transcription factors (TF) in SRSF1-KO CD8 + T cells and mTOR pathway related TFs. KO: Knockout. **l** Foxo1 expression in UMAP plot from scRNA-seq analysis. **m** Western blot assays showed CD38 and Foxo1 protein levels in Srsf1^+/+^ and Srsf1^–/–^ CD8 + T cells when Foxo1 was silenced. **n** ChIP assays showed Foxo1 transcriptionally upregulated Cd38. **o** Western Blot assay showed Srsf1^–/–^Cd8+T cells could decrease the expression of Nlk. **p** Luciferase analysis showed SRSF1-RRM1 was binding to 3ʹUTR of Nlk mRNA. **q** The study compares the mRNA expression levels of Nlk, Foxo1, and Pten in control versus Srsf1^+/–^ Cd8+ T cells. Data presented as mean ± S.E.M. A two-tailed unpaired *t* test was used to assess statistical significance. Significance levels are denoted as follows: * for *p* < 0.05, ** for *p* < 0.01, *** for *p* < 0.001, **** for *p* < 0.0001, and ‘ns’ indicates no significance

Subsequently, we investigated the mechanism through which Srsf1 influenced CD8^+^ T cell immunity. Firstly, we performed RNA-seq analysis of Srsf1^–/–^ and Srsf1^+/+^ CD8^+^ T cells derived from spleens of 6-week-old Srsf1^+/+^Cd4-Cre or Srsf1^fl/fl^Cd4-Cre C57BJ/6 mice (Fig. 3a). KEGG pathway analysis identified the leading pathways as those involving genes related to T cell activation, the PI3K/AKT/mTOR pathway, and cytokine-cytokine receptor interactions (Fig. 3b). A significant upregulation of T cell cytotoxicity genes (Ifng, Gzma, Cd38) and PI3K/AKT/mTOR associated genes were further seen in Srsf1-deficient CD8 + T cells (Fig. 3c–e), which were also validated by qPCR assays (Fig. 3f). Research indicates that SRSF1 can suppress mTOR pathway activity by upregulating PTEN expression in naive CD4 + T cells from systemic lupus erythematosus patients.^24–26^ We examined PTEN protein expression, a negative regulator of the mTOR pathway, and found that PTEN levels decreased while phosphorylated S6 (pS6) protein levels increased in CD8^+^T cells following anti-CD3 and anti-CD28 stimulation in Srsf1^fl/fl^Cd4-Cre mice (Fig. 3g), suggesting heightened mTORC1 pathway activity. To determine if heightened mTORC1 activation in CD8^+^ T cells from Srsf1^fl/fl^Cd4-Cre mice influences their proinflammatory phenotype, we investigated the impact of rapamycin, given SRSF1’s role in posttranscriptional gene regulation. In vitro treatment of CD8^+^ T cells from Srsf1^fl/fl^Cd4-Cre mice with rapamycin decreased both Ifn-γ production and glycolytic metabolism (Fig. 3h, i). SRSF1 includes two RNA recognition motifs, RRM1 and RRM2, which facilitate its specific RNA interactions. To evaluate the hypothesis, we generated vectors encoding HA fusion constructs of various SRSF1 domains, including variants with deletions in the RRM1 domain (∆RRM1), RRM2 domain (∆RRM2), RS domain (∆RS), and an empty vector (EV) as a control (Fig. 3j). Our study found that Pten’s 3ʹUTR activity significantly decreased in Srsf1^-/-^ CD8^+^ T cells, but was restored with the addition of Srsf1, Srsf1-∆RRM2, or Srsf1-∆RS, suggesting Srsf1’s binding to Pten’s 3ʹUTR in CD8^+^ T cells (Fig. 3j).

To investigate the mechanism behind Cd38 upregulation in Srsf1-depleted Cd8^+^ T cells, we conducted a transcription factor (TF) analysis using RNA-seq data. By intersecting these results with TFs related to mTOR pathways, FOXO1 emerged as the sole potential TF responsible for upregulating Cd38 expression in T cells (Fig. 3k), validated in scRNA-seq analysis (Fig. 3l). Western blot assays demonstrated that knocking out Srsf1 in Cd8^+^T cells significantly upregulated the expression of Cd38 and Foxo1. Additionally, the upregulation of Cd38 was reversed when Foxo1 was downregulated (Fig. 3m). Additional ChIP assays demonstrated that Foxo1 could enhance Cd38 expression at the transcriptional level in Cd8^+^T cells (Fig. 3n). Finally, we re-explored how Srsf1 regulated Foxo1 in Cd8^+^T cells. A recent study demonstrated that elevated NLK phosphorylation facilitates the nuclear export of FOXO1, thereby inhibiting its self-transcriptional activity and expression in the liver.^27^ PCR and western blot analyses revealed a significant downregulation of Nlk in Srsf^1–/–^ Cd8^+^T cells (Fig. 3o). Subsequently, we examined how Srsf1 influences the 3ʹ UTR activity of Nlk. We found that the 3ʹUTR activity of Nlk significantly decreased in Srsf1^–/–^ Cd8^+^ T cells (Fig. 3p). This activity was restored upon the introduction of Srsf1, Srsf1-∆RRM2, or Srsf1-∆RS, suggesting that Srsf1 binds to the 3ʹUTR of Nlk in Cd8^+^T cells (Fig. 3p) and validated in Cd8^+^T cells with heterozygous SRSF1 deletion (Fig. 3q).

### SRSF1 within tumors limits the activation and function of CD8^+^ T cells

Several studies have demonstrated that SRSF1 was associated with cell malignancy and highly elevated in multiple cancers, including HCC. The impact of reduced tumor-intrinsic SRSF1 expression on T cell-mediated antitumor activity is not yet understood. We employed shRNA to suppress SRSF1 expression in Hep1-6 cells expressing ovalbumin (Hep1-6-OVA; Supplementary Fig. 3a). An intriguing finding emerged when SRSF1-deficient tumor cells were introduced into various mouse strains: while SRSF1 knockdown slightly hinders Hep1-6-OVA tumor growth in nude mice, the effect is significantly greater in immunocompetent C57BL/6 mice (Supplementary Fig. 3b–d). These variations imply that tumor intrinsic SRSF1 may also influence the T-cell compartment.

We noted a marked increase in CD8^+^ T cell infiltration within tumor tissues of SRSF1-sh Hep1-6-OVA tumors (Supplementary Fig. 3e). To directly validate the impact of T cells on the restrained tumor growth observed in SRSF1-sh tumors, we depleted CD8^+^ T cells in two groups. Upon depletion of CD8 + T cells (Supplementary Fig. 3f), tumor growth was delayed in SRSF1-sh group. We subsequently assessed the functionality of tumor-infiltrating CD8^+^ T cells by measuring their IFN-γ and GZMB production. SRSF1-sh tumor cells exhibited strong signals for both IFN-γ and GZMB, in contrast to control tumors, which produced minimal amounts of these cytokines (Supplementary Fig. 3g).

We next investigated whether SRSF1 within tumor cells could directly undermine T cells’ cytotoxic functions when recognizing tumors in response to TCRs. We investigated the effect of tumor-intrinsic SRSF1 on T cells by co-culturing Hep1-6-OVA cells with OTI CD8^+^ T cells.^28^ We conducted RNA sequencing on OTI CD8^+^ T cells sorted by flow cytometry after 8 h of co-culture (Supplementary Fig. 3h). Transcriptome analysis revealed significant changes in co-cultured CD8^+^ T cells due to SRSF1-sh in tumor cells (Supplementary Fig. 3h). In CD8^+^ T cells, differentially expressed genes (DEGs) showed increased expression of cytotoxic molecules such as PRF1, IFN-γ, and GZMB (Supplementary Fig. 3h). GSEA of CD8^+^ T cell effector signature genes consistently showed that T cells co-cultured with SRSF1-sh tumor cells were in a more cytotoxic state compared to those co-cultured with control cells (Supplementary Fig. 3i). SRSF1-sh cells exhibited increased susceptibility to T cell-mediated cytotoxicity compared to control Hep1-6-OVA cells (Supplementary Fig. 3j). The SRSF1-sh co-cultured T cells demonstrated robust IFN-γ and GZMB production (Supplementary Fig. 3k). These findings indicate that SRSF1 within tumor cells acts as a suppressive molecule, limiting T cell activation and effector states.

### Tumor-intrinsic SRSF1 was associated with glycolytic metabolism in HCC cells

Tumor glucose consumption is well-known to metabolically restrict T cells, impairing their cytotoxic functions and accelerating tumor progression.^29–33^ We assessed the impact of SRSF1 on glycolytic metabolism by measuring glycolytic capacity with a Seahorse instrument. The results indicated that SRSF1-sh cells exhibited a significantly reduced glycolytic capacity compared to control Hep1-6-OVA cells (Supplementary Fig. 4a, b). Untargeted metabolomics was performed to find that the downregulated metabolites were significantly associated with central carbon metabolism when upon SRSF1-sh (Supplementary Fig. 4c).

In line with Seahorse assay findings, glycolysis pathway metabolite levels were reduced in SRSF1-sh cells (Supplementary Fig. 4d). Additionally, SRSF1 significantly inhibited 18F-FDG uptake in nude or immunocompetent C57BL/6 mice (Supplementary Fig. 4e). To confirm SRSF1’s glycolytic function in HCC tissues, PET/CT imaging of 40 patients revealed significantly higher SUV_max_ values in the high SRSF1 group compared to the low SRSF1 group (Supplementary Fig. 4f). We hypothesized that SRSF1 could be linked to HCC metabolism, with a particular focus on glycolysis. The SRSF1 high group exhibited a significant increase in glycolysis-related L-lactate levels (Supplementary Fig. 4f). The heatmap result showed that glycolysis related genes were also downregulated when SRSF1 was silenced in tumor cells (Supplementary Fig. 4g). These findings suggest that SRSF1’s role in promoting HCC is closely linked to its influence on the glycolytic metabolic pathway.

### Tumor-intrinsic SRSF1 promotes glycolysis by upregulating bZIP and c-myc expression

**Fig. 4:**
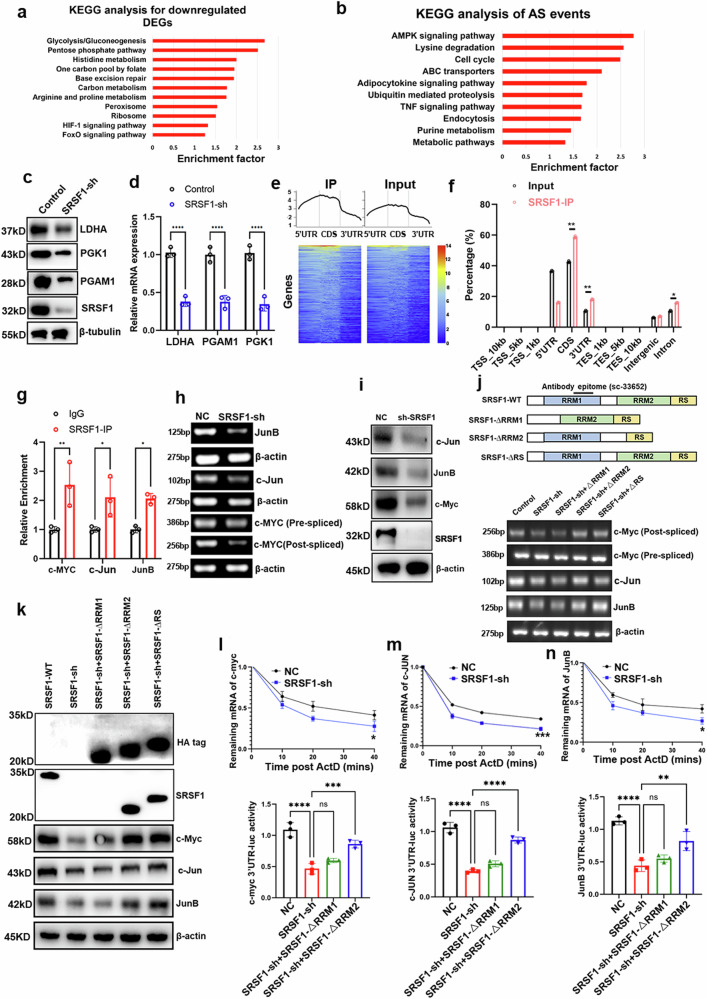
Reducing tumor-intrinsic SRSF1 suppresses glycolysis by downregulating several bZIP transcription factors and c-myc. **a** KEGG of SRSF1-downregulated genes. **b** KEGG of SRSF1-regulated AS events. **c**, **d** Western Blot and qPCR assays showed SRSF1 regulated glycolytic genes. **e**, **f** SRSF1-RIP-seq peaks showed enrichment in the 5ʹ UTR, CDS, and intronic regions. RIP-seq peaks were classified based on their distribution across various genomic elements and analyzed against the genomic background. **g** The RIP assays showed SRSF1 was binding to c-myc, c-Jun and JunB. PCR (**h**) and Western Blot (**i**) assays showed SRSF1 regulated c-myc, c-Jun and JunB transcription factors. **j** The schematic diagram of SRSF1 or mutants of SRSF1 proteins. **k** Western blot analysis of transcription factor expression in SRSF1-sh cells transfected with both endogenous and exogenous SRSF1 using anti-HA. SRSF1 mutants include ΔRRM1 (RRM1 deletion), ΔRRM2 (RRM2 deletion), and ΔRS (RS deletion). All mutants were tagged with HA. **l**–**n** SRSF1 could protect c-myc, c-Jun and JunB transcription factors by binding to 3ʹUTR of mRNAs. Data are presented as mean ± S.E.M. and analyzed using one-way ANOVA with a multiple comparison test, where significance is indicated as follows: ***p < 0.001, **p < 0.01, *p < 0.05

We conducted RNA sequencing on SRSF1-sh and control Hep1-6-OVA tumor cells to examine the impact of SRSF1-sh on glycolytic metabolism. Among the SRSF1-downregulated DEGs, those linked to tumor-related functions were primarily involved in glycolytic metabolism, the HIF1a signaling pathway, and metabolic pathways, rather than the upregulated genes (Fig. 4a). KEGG of alternative splicing events showed AMPK signaling pathway and cell cycle were the top terms (Fig. 4b), which indicated RNA splicing may not be responsible for glycolytic metabolism when SRSF1 was knockdown. We discovered that genes encoding glycolysis enzymes, such as PGK1, PGAM1, and LDHA, were identified (Supplementary Fig. 4g), and were significantly downregulated upon SRSF1 knockdown from RNA-seq analysis, which were validated by qPCR and Western blot assays (Fig. 4c, d).

Transcription factors, such as the MYC (cMyc) and bZIP families, are recognized for activating genes encoding glycolysis enzymes. We hypothesized that the downregulation of glycolysis enzymes following SRSF1-sh could be due to the suppressed expression of related transcription factors. Given SRSF1’s role as an RNA-binding protein influencing posttranscriptional gene expression through mRNA stability,^11,13,17^ we conducted SRSF1-RNA Immunoprecipitation (RIP) sequencing. The analysis revealed that SRSF1-associated RNA peaks are predominantly enriched in exons, 3ʹ UTR, and introns (Fig. 4e, f, Supplementary Table 4). We intersected RIP and RNA-seq analysis results to identify potential regulators linked to SRSF1-enhanced glycolytic cell malignancy in HCC. Our RNA-seq and RIP-seq analyses revealed that several transcription factors from the bZIP and MYC families, such as Jun (c-Jun), Junb (JunB), and c-myc, bind with SRSF1 and are downregulated at both RNA and protein levels following SRSF1-sh (Fig. 4g–i).

To explore the specific roles of RRMs in binding to those transcription factors (Fig. 4i). RT-PCR analysis of Hep1-6 cells transfected with various vectors demonstrated that ∆RRM2 and ∆RS produced effects like the control group, notably a significant up-regulation of mature c-MYC, JunB, and c-Jun mRNA (Fig. 4j). This pattern was similarly noted in the protein levels (Fig. 4k). In alignment with our earlier findings, the lack of impact from SRSF1 domains on un-spliced c-MYC mRNA suggests that SRSF1’s influence on c-MYC expression is mainly posttranscriptional. The findings indicate that the RRM1-rich domain of SRSF1 is essential for safeguarding the mRNA of these factors from degradation. To test this hypothesis, we investigated the impact of transcription inhibition using actinomycin D (ActD), both with and without SRSF1 knockdown. The study found that SRSF1 knockdown significantly reduces mature c-MYC, JunB, and c-Jun mRNA levels, indicating that SRSF1 plays a protective role against their degradation (Fig. 4l–n), which mainly caused by SRSF1 binding to the 3ʹUTR of bZIP transcription factors (Fig. 4l–n). Finally, ChIP assays showed that bZIP transcription factors, including c-Jun and JunB, and c-Myc transcriptionally upregulate the expression of LDHA, PGAM1 and PGK1, respectively (Supplementary Fig. 4h–k). Overexpression of specific transcription factors can counteract the impact of SRSF1 depletion on glycolytic gene expression (Supplementary Fig. 4l). Taken together, these data suggest SRSF1 upregulated the glycolytic genes by regulating the c-Myc, JunB and c-Jun mRNA stability.

### Development of TN2008 as a new potent SRSF1 inhibitor

**Fig. 5:**
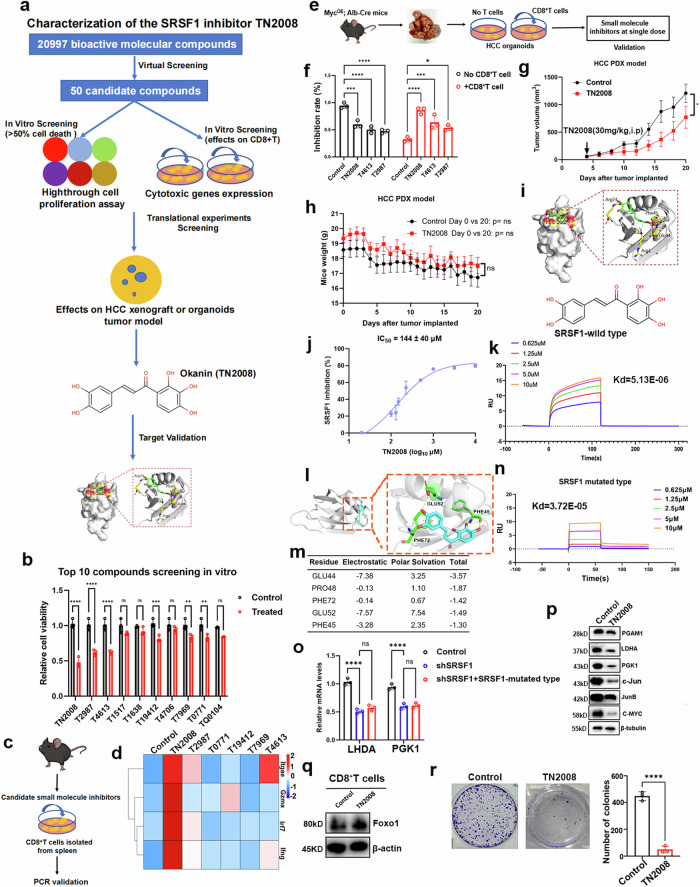
Characterization of the SRSF1 inhibitor TN2008. **a** The schematic diagram of SRSF1 inhibitor screening. **b** In vitro proliferative assays screened top SRSF1 inhibitor candidates. **c** The schematic diagram of SRSF1 inhibitor acting on CD8^+^T cells. **d** Heatmap showed SRSF1 inhibitors increased cytotoxic or memory genes of CD8^+^T cells. **e** Construction of a co-culture system for murine organoids and CD8^+^T cells. **f** Bar plots showed SRSF1 inhibitor TN2008 provided the highest inhibition rate when co-cultured HCC organoids and CD8^+^T cells. **g**, **h** SRSF1 inhibitor TN2008 inhibited the tumor growth in PDX mice model. **i** Overview of structural complex of SRSF1 bound with TN2008. **j** In vitro assessment of IC50 to evaluate TN2008’s inhibitory effect on SRSF1-mediated tumor growth inhibition (*n* = 3). **k** SPR assays showed SRSF1 recombination protein specifically bound with TN2008. **l** The homology model of the SRSF1 protein is in complexity with TN2008 by molecular dynamic simulation. **m** Key residues and their energy contributions were determined through MD simulation and MM/PBSA analysis. **n** Surface plasmon resonance (SPR) analysis demonstrated the interaction between TN2008 and the mutated SRSF1 protein. **o** qPCR assays showed SRSF1-mutated residues were responsible for glycolytic genes expression in tumor cells. **p** Western blot analysis demonstrated that TN2008 reduced glycolytic gene expression in tumor cells. **q** Western blot analysis demonstrated that TN2008 elevated Foxo1 expression in murine CD8^+^ T cells. **r** Colony formation assays showed TN2008 significantly inhibited tumor cell growth in vitro. Data presented as mean ± S.E.M. Statistical significance was assessed using a two-tailed unpaired *t* test and two-way ANOVA. Significance levels are denoted as follows: * for *p* < 0.05, ** for *p* < 0.01, *** for *p* < 0.001, **** for *p* < 0.0001, and ‘ns’ indicates no significance

Considering SRSF1’s dual role in tumorigenesis within tumors and T cells, we hypothesized that pharmacological inhibition of SRSF1 could enhance T cell-mediated antitumor immunity by simultaneously affecting both tumors and T cells. However, no potent inhibitor for SRSF1 is established to date. Our goal is to identify specific pharmacological inhibitors for SRSF1; however, the crystal structure of the SRSF1 protein has not yet been reported. Consequently, homology modeling was employed to predict the 3D structure of the SRSF1 protein. Our study highlights the crucial role of the RRM1 domain in SRSF1 protein function, prompting us to target this domain in our search for the first SRSF1 inhibitor among 20,997 small molecular compounds from the Drugbank database (Fig. 5a, Supplementary Fig. 5a). The predicted structure of SRSF1 was built (Supplementary Fig. 5a) and virtual high-throughput screening identified the top 50 pharmacological candidates for repositioning as the first SRSF1 inhibitors (Supplementary Tables 5, 6). In the primary cytotoxicity assays using a single concentration, we identified the following several significant SRSF1 inhibitors: TN2008, T4613 and T2987 (Fig. 5b). Given the dual role of SRSF1 on tumor and T cells, we selected the significant candidate inhibitors screening by cytotoxicity assays in vitro and added into CD8^+^T cells at single dose (100 μM), we found that only TN2008, T4613 and T2987 have more effects on upregulation of CD8 + T cell (Fig. 5c, d). Additionally, we also evaluated these inhibitors in the established HCC organoids co-cultured with CD8^+^T cells by c-myc^OE^; Alb-cre mice, we found that TN2008 was the most significant inhibitor for tumor growth (Fig. 5e, f). Our investigation into the impact of TN2008 on patient-derived xenografts (PDX) revealed a significant reduction in tumor growth compared to the control group (Fig. 5g, h). TN2008 was selected as an effective SRSF1 inhibitor for HCC patients, demonstrating its ability to suppress LPS-induced TLR4 expression in BV-2 microglial cells.^34^ Our study revealed that TN2008 effectively inhibits SRSF1 protein function, exhibiting a binding energy of –5.1 kcal/mol (Fig. 5i). In vitro assays demonstrated that TN2008 effectively inhibited SRSF1 activity, with an IC50 of approximately 144 µM (Fig. 5j). We also determined the protein structure of SRSF1-RRM1 in complex with TN2008 to elucidate the molecular mechanism of SRSF1-TN2008 binding (Supplementary Fig. 5a). We examined TN2008’s target engagement in cells, revealing through Biacore T100 binding kinetic studies that TN2008 exhibits high affinity for recombinant SRSF1, with a dissociation constant of 5.13e-6M, while showing no significant binding to other SRSF family members (Fig. 5k; Supplementary Fig. 5b).

Molecular docking and dynamics simulations suggested that TN2008 was likely to interact with the SRSF1 protein, primarily involving the amino acids GLU44, PRO48, GLU52, PHE72, and PHE45, as indicated by amino acid free energy decomposition experiments (Fig. 5l, m). To validate the computational prediction, we purified both wild-type SRSF1 proteins and SRSF1 variants with mutations at GLU44, PRO48, GLU52, PHE72, and PHE45. Surface plasmon resonance (SPR) assays demonstrated that TN2008 binds to SRSF1 with a dissociation constant (Kd) of 5.13e–6 (Fig. 5k), which was disrupted when GLU44, PRO48, GLU52, PHE72 and PHE45 of SRSF1 were mutated (Kd=3.72e-05) (Fig. 5n). Similar results showed that glycolytic genes cannot be rescued by overexpression of SRSF1 mutated protein in shSRSF1 tumor cells (Fig. 5o). The results demonstrate that TN2008 selectively binds to the wild-type SRSF1 protein over its mutants, highlighting the essential roles of GLU44, PRO48, GLU52, PHE72, and PHE45 in this interaction. Subsequent evaluation of TN2008 in Hep1-6-OVA cells revealed its inhibitory effect on glycolytic gene expression (Fig. 5p). Furthermore, TN2008 has the potential to enhance Foxo1 expression in Cd8^+^T cells (Fig. 5q). Similarly, TN2008 treatment exhibited significant inhibition in cell growth in vitro (Fig. 5r). In summary, TN2008 effectively inhibits SRSF1, reducing tumor cell growth.

### Targeting SRSF1 boosts T cell functionality and synergizes with anti-PD-1 therapy

We first used in vitro co-culture assays to assess if TN2008 has antitumor effects comparable to SRSF-sh. T cells co-cultured with TN2008-pretreated Hep1-6-OVA tumor cells exhibited increased cytokine release and heightened cytotoxicity (Supplementary Fig. 5c). The impact of TN2008 on T cell function was previously unestablished. To answer this question, we treated mouse CD8^+^ T cells with TN2008 for 24 h. We observed increased IFN-γ and GZMB, indicating that it caused activation phenotype of CD8 + T cells (Supplementary Fig. 5d, e).

**Fig. 6:**
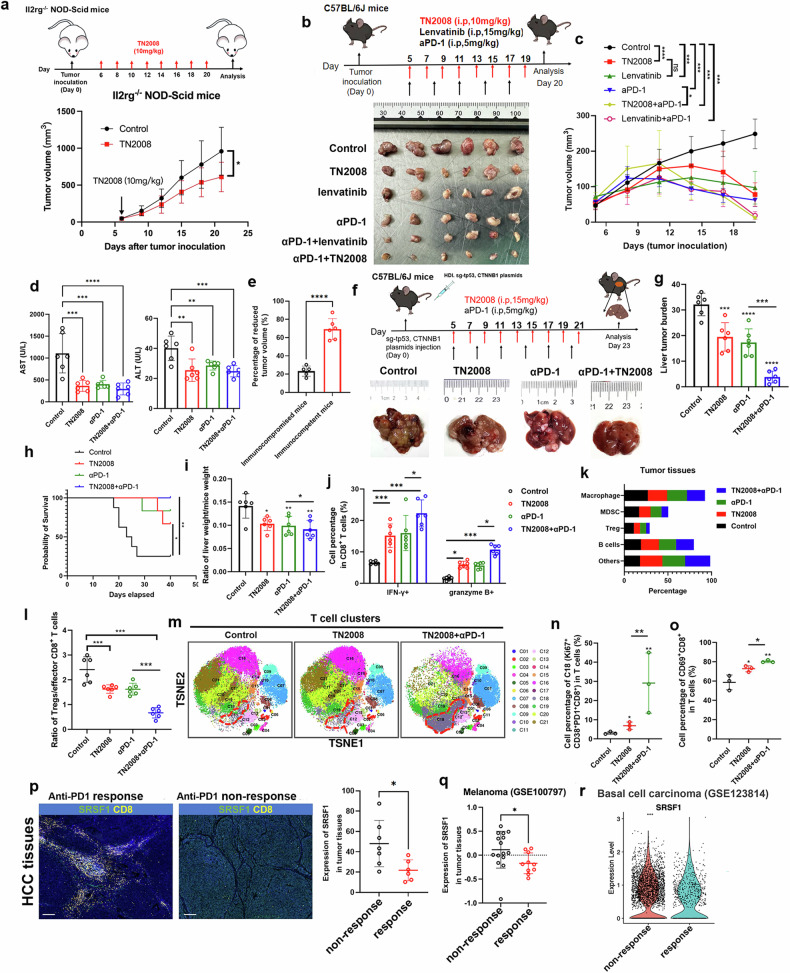
The SRSF1 inhibitor TN2008 enhances T cell infiltration and functionality and works synergistically with anti-PD-1 therapy. **a** The inhibitory effect of TN2008 on subcutaneous tumors in ll2rg-/-NOD-Scid mice. **b**, **c** TN2008 synthesizes PD-1 blockade in subcutaneous tumor model. **d** TN2008 could alleviate the function of murine liver from HCC tumor mice model. **e** Percentage of reduced tumor volume in two different mice groups. **f** TN2008 synthesizes PD-1 blockade in murine HCC spontaneous model. **g** liver tumor burden in four different groups. **h** Kaplan-Meier survival analysis between four different groups. **i** Liver-to-body weight ratio across various groups of mice. **j** Percentage of effector CD8 + T cell in four different groups. **k** Other cell percentage in different four groups. **l** The ratio of regulatory T cells (Tregs) to effector CD8 + T cells across four groups. **m** CyTOF analysis was conducted to examine T cells within the tumor microenvironment across three distinct groups. **n** The percentage of CD38^+^CD8^+^T cells among different groups. **o** The percentage of CD69^+^CD8^+^T cells among different groups. **p** The expression of SRSF1 in PD-1 responsive or non-responsive HCC cases. **q** The expression of SRSF1 in melanoma tissues among respond or non-respond cases from public database. **r** The expression of SRSF1 in basal cell carcinoma among respond or non-respond cases from public database. Data presented as mean ± S.E.M. Statistical significance was assessed using a two-tailed unpaired *t* test, two-way ANOVA, log-rank test, and Wilcoxon signed-rank test. ^*^p < 0.05, ^**^p < 0.01, ^***^p < 0.001, ^****^p < 0.0001

To evaluate the antitumor efficacy of TN2008, Hep1-6-OVA cells were inoculated into C57BL/6 mice, which were then administered TN2008 at doses of 5 mg/kg, 10 mg/kg, and 15 mg/kg daily from day 7 to day 15. TN2008 treatment effectively inhibited tumor growth in vivo in a dose-dependent manner, with 15 mg/kg showing optimal efficacy (Supplementary Fig. 5f–i). Then, we inoculated Hep1-6-OVA cells into ll2rg-/- NOD-Scid mice, which was highly immunocompromised, we treated mice with TN2008 at 10 mg/kg and found that TN2008 also effectively inhibited tumor growth (Fig. 6a).

We explored whether combining TN2008 with PD-1 blockade could enhance therapeutic outcomes and if TN2008 offers comparable or superior effects to Lenvatinib. Mice treated with the combination therapy showed reduced growth of Hep1-6 tumors compared to those in the monotherapy groups (Fig. 6b, c, Supplementary Fig. 6a). Further analysis indicated no variations in liver function tests across different groups, implying that TN2008 may exhibit limited overall and hepatic toxicity in mouse models (Fig. 6d). Interestingly, the tumor growth inhibited by TN2008 in immunocompetent C57BL/6 mice was more pronounced than in immunocompromised mice (Fig. 6e).

We then validated these findings in *CTNNB1*^*N90*^*; Trp53*^*KO*^ induced murine HCC model, in which combinational therapy exhibited slower tumor growth, better overall survival and an amelioration of TME (Fig. 6f–l). Cytometry by Time-Of-Flight (CyTOF) analysis revealed an increase in effector CD8^+^ T cells (CD69^+^ CD8^+^) and Ki67^+^CD38^+^CD8^+^ T cells, alongside a decrease in Treg and MDSC cells in both TN2008 monotherapy and combination therapy (Fig. 6m–o; Supplementary Fig. 6b–e).

Finally, SRSF1 from tumor cells was expressed higher in HCC non-PD-1 respond group than in respond group (Fig. 6p), as validated in other cancers, including melanoma (Fig. 6q) and basal cell carcinoma (Fig. 6r). B16F10 melanomas are reported to resist both anti-PD-1 monotherapy and combination therapy with checkpoint antibodies. PD1 blockade treatment of TN2008 tumors significantly inhibited tumor growth, enhanced survival, and improved the TME (Supplementary Fig. 6f–j). These findings demonstrate that TN2008 effectively induces T cell-mediated antitumor responses and enhances immunotherapy efficacy synergistically.

## Discussion

Here, we report on a novel immunotherapy strategy for cancers resistant to checkpoint blockades. Numerous human cancers exhibit resistance to PD-1 antibodies, often characterized by a notable absence of CD8^+^ T cell infiltration, resulting in ‘cold’ tumors.^35^ These tumors lack the immunogenicity needed to trigger a spontaneous T cell response that checkpoint blockade could amplify. Previous studies emphasized that metabolic restriction imposed in the TME greatly alters the cellular responses of CD8^+^ T cell or tumor cells leading to dually impaired anti-tumor immunity.^32^ Metabolic interventions are necessary to enhance T cell effector functions during these cellular events. Our findings demonstrate that inhibiting the splicing factor SRSF1 can overcome barriers to effective cancer immunotherapy (Supplementary Fig. 7).

Lactic acid is highly concentrated in the TME and is known to exert immunosuppressive effects, particularly inhibiting the function of conventional T cells in vitro.^32^ Our study demonstrates that the inhibition of SRSF1, a well-characterized RNA splicing factor in tumor cells, can reduce lactic acid production, thereby enhancing the efficacy of cancer immunotherapy. Notably, SRSF1 inhibition in either tumor cells or CD8^+^T cells yield significant benefits for T cell-mediated tumor immunity. Specifically, the inactivation of SRSF1 in tumor cells leads to a reduction in glycolytic metabolism and results in a marked increase in the number of tumor-infiltrating CD8^+^ T cells, along with a decrease in regulatory T cells (Tregs) within the TME. Furthermore, these tumor-infiltrating CD8^+^ T cells exhibit enhanced functionality and reduced expression of exhaustion markers. Inactivating SRSF1 in CD8^+^ T cells preserve a substantial pool of cytotoxic CD8^+^T cells that possess improved anti-tumor activity. These findings are particularly significant, as T cell exhaustion is recognized as a major barrier to effective tumor immunity. We provide compelling evidence that these results are applicable to a variety of human cancers. Analysis of The Cancer Genome Atlas (TCGA) data revealed elevated SRSF1 mRNA levels across numerous human cancer types, including those that have been largely resistant to checkpoint blockade therapies, such as melanoma and non-small cell lung cancer. Moreover, SRSF1 mRNA levels were negatively correlated with gene expression signatures indicative of cytotoxic CD8^+^ T cells, while showing a positive correlation with signatures of exhausted CD8^+^ T cells. These human data align with previous studies that have established a link between the cytotoxic CD8^+^ T cell signature and T cell-inflamed (‘hot’) tumors, in contrast to non-T cell inflamed (‘cold’) tumors.

Metabolic processes almost entirely drive T cell survival, activation, development, proliferation, differentiation, and antitumor effector function.^29,30,36^ Prior to activation, ATP produced mostly by OXPHOS supports naive T cells.^36^ During T cell activation, dynamic T cell activation signaling networks were used to map the T cell proteome and phosphoproteome.^36^ For the sake of cell proliferation and effector functions, activated T cells changed their metabolic state from oxidative metabolism to glycolysis.^30,31,36^ The precise function of SRSF1 in CD8 + T metabolism or T cell-mediated antitumor immunity is yet unknown, despite some research on the protein’s activity in T cells. We have demonstrated that CD8 T cells and tumor cells respond differently metabolically to SRSF1 suppression. In particular, T cell expansion of CD8 + T cells and effector activity in malignancies were significantly enhanced by SRSF1 inactivation. When compared to control CD8 + T cells, RNA-seq analysis of SRSF1-knockout (KO) cells revealed that SRSF1 inactivation increased glycolytic metabolism, which is known to enhance T cell-mediated tumor immunity. Additionally, the mTOR pathway and transcription factors essential for memory T cell differentiation, self-renewal, and persistence, such as Itgae and Irf7, were more active in SRSF1-KO T cells.^30,31,36^

A recent study demonstrated that by inhibiting ferroptosis, mTORC signaling serves as a crucial signaling hub to increase the lifespan of virus-specific memory CD4^+^T cells.^37^ Furthermore, a scRNA-seq study of CD8 T cells in human melanomas showed that patients receiving checkpoint inhibition had a better prognosis when CD8^+^T cells expressed more TCF7.^35,37^ Higher levels of Itgae, which codes for a transcription factor that stimulates the development of memory T cells, were expressed by SRSF1-KO T cells. Previous investigation has demonstrated that Itgae overexpression improves CD8 + T cell memory formation, polyfunctionality, and protective anti-tumor immunity.^38^ Therefore, in the context of knockout engineering of novel components as immune boosters, the metabolic effects of SRSF1 make it an intriguing target for T cell metabolic programming.

Why do T cells react so differently to SRSF1 suppression than do tumor cells? By regulating the expression of FOXO1 and CD38, inactivating SRSF1 in T cells dramatically increases the cytotoxicity of CD8 + T cells in malignancies. Depleting SRSF1 in tumor cells reduced glycolytic metabolism and activated CD8^+^ T cells by limiting abnormal alternative splicing of transcription factors and genes related to glycolysis. However, the expression of mTOR was reduced while PTEN increased when SRSF1 was downregulated in tumor cells (Supplementary Fig. 6k). While in CD8 + T cells, mTOR was activated and we hypothesized that FOXO1 was inactivated, however, in SRSF1-KO group, FOXO1 was activated due to decrease of NLK expression, which could facilitate the nuclear export of FOXO1. Notably, the genes differentially expressed due to SRSF1 gene inactivation showed minimal overlap between tumor cells and T cells (Supplementary Fig. 6l). The opposite effects triggered by SRSF1 in tumor or CD8 T cells may be explained by the differences from specific cell-type characteristics.

Together, we show that suppression of SRSF1 in CD8 + T cells improve antitumor efficacy in vivo, promotes T cell activation and effector function, and rewires T cell metabolism. Furthermore, by inhibiting SRSF1, TN2008 can reduce glycolytic metabolism in tumors, avoiding the metabolic constraint that is known to impair T cell effector functions within tumors. Importantly, combining SRSF1 inactivation in tumor cells with PD-1 blockade significantly improved survival in B16F10 melanoma, Hep1-6 subcutaneous tumors, and *CTNNB1*^*N90*^*; Trp53*^*KO*^ autochthonous murine HCC models. Notably, TN2008 demonstrated synergistic effects with anti-PD-1 therapy in the B16F10 melanoma model, which is resistant to PD-1 mAb. The inhibitor significantly boosted tumor infiltration and improved the functionality of cytotoxic CD8 T cells. These findings justify targeting SRSF1 in human cancers that are resistant to existing immunotherapies. This method could benefit checkpoint blockade therapy and other immunotherapies where T cells are crucial, such as neoantigen-based cancer vaccines and CAR T cell therapies for solid tumors.

## Materials and methods

### Tissue samples and clinicopathological data

Surgical specimens of 250 tumor tissues and paired adjacent non-tumor cirrhotic liver tissues were obtained from patients undergoing curative resection in 2010 at the Liver Cancer Institute, Zhongshan Hospital, Fudan University, with written consent. After surgical excision, 500 specimens were fixed immediately in 3.7% buffered formaldehyde solution and embedded in 500 paraffin. Then, 5 mm continuous sections were prepared for IHC of SRSF1. All patients were post surgically monitored until June 20, 2015. The histopathologic diagnosis was based on the World Health Organization criteria. The tumor grade was determined by the classification proposed by Edmondson and Steiner. Tumor stage was determined according to the Tumor-Node-Metastasis classification system established by the 2010 International Union Against Cancer. The Research Ethics Committee of Zhongshan Hospital approved the ethical use of human subjects for this study, and informed consent was obtained from each patient. Ethical consent was granted from the Committee on Ethics of Zhongshan Hospital, Fudan University, following Institutional Review Board approval (Y2020-622). OS was defined as the interval between surgery and death or between surgery and the last observation point. Additionally, RNA-seq data from a total of 372 human HCC samples and corresponding clinical information were downloaded from TCGA (https://cancergenome.nih.gov/).

### Cell type determination

The filtered, quality-controlled single-cell count matrix was first normalized using log-normalization method (Seurat “NormalizeData” function). We identified highly variable genes by using a normalized expression range of 0.125–3 and a quantile-normalized variance greater than 0.5. Then, we used principal component analysis to reduce the number of dimensions representing each cell, and the top 50 principal components were used as input for Harmony v0.1, which corrected the batch effects resulting from sample sources. We utilized the initial 20 Harmony dimensions for tSNE or UMAP dimensionality reduction, employing the default settings of the Run tSNE and UMAP functions in the Seurat package. To identify and annotate different cell clusters, the nearest-neighbor graph was first constructed using “FindNeighbors” function based on the top 20 Harmony dimensions. Then, the Louvain clustering method was performed using the constructed nearest-neighbor graph with “FindCluster” function. The resolution parameter was set to 1 for major cell type identification and 0.8 for subtype characterization of major cell types, other clustering parameters were set to default. Cell clusters identified through Louvain clustering were manually annotated to known cell types by using marker genes listed in Supplementary Table 3.

### Cell preparation

HCC and melanoma cell lines were used in this study. The MHCC97H cell lines, known for their high metastatic potential in human hepatocellular carcinoma (HCC), were developed at our institute. Hep1-6, murine HCC cell lines and B16F10 were purchased by Shanghai ATCC Cell Bank. Cells were maintained at 37 °C with 5% CO^2^ in Dulbecco’s modified Eagle’s medium (Invitrogen) supplemented with 10% fetal bovine serum.

### Immunohistochemistry

IHC was performed with rabbit anti-human SRSF1 (1:100; Cat# ab129108, Abcam). Phosphate-buffered saline (PBS) was used to substitute the primary antibody as a negative control (only with bio-IgG and ABC complex). IHC images were acquired under a DM6000B microscope (Leica, Germany). The expression of SRSF1 in HCC patients was divided according to the median expression of SRSF1 IHC.

### RNA immunoprecipitation assays

Cells overexpressing SRSF1 were used to perform RIP experiments using a SRSF1 antibody (5 mg per reaction) and the Magna RIP RNA-Binding Protein Immunoprecipitation Kit (Millipore) according to the manufacturer’s instructions. In brief, cells were cross-linked in 0.1% formaldehyde prior to lysis. Cell lysates were sonicated and immunoprecipitated, and the eluates were reverse-crosslinked. Relative occupancy values were calculated by determining the IP efficiency and normalized to the level observed by immunoprecipitation using non-specific IgG.

### Immunofluorescence staining

Tumor tissues were collected from Hep1-6-OVA shNC/shSRSF1 tumor bearing mice on day 15. Tumor tissues were embedded in OCT (Sakura 4583) and froze in – 80°C. Tissues were cut into 8 mm pieces transversally and adhered to microscope slides (ZSGB-BIO ZLI-9506). Sections were then blocked with 5% goat serum (ZSGB-BIO) for 1 hr and incubated with antibodies directly against CD8a (KT15) at 4°C overnight in the dark. The slides were washed 3 times with PBS. 1 mg/ml DAPI (Life technology) were added and incubated for 5 min. After a final wash step, sections were mounted using the Fluoromount-G (SouthernBiotech 0100-01). Immunofluorescence was visualized utilizing a confocal microscope (ZEISS LSM880).

### ChIP assays

ChIP assays were performed using a NFATC2 antibody (5 mg per reaction; MA1-025, 25A10.D6.D2, ThermoFisher), and the EZMagna ChIP A/G (17-10086, Millipore) according to the manufacturer’s protocols. ChIP derived DNA was quantified using real-time qPCR analysis. The primers specific for the SRSF1 and NFATC2 promoters are shown in Supplementary table 7.

### T cell co-culture assay

For SRSF1-Kd cells, 1 × 10^5^ Hep1-6-OVA cells per well were seeded into the 96-well plates (NEST) with RPMI 1640 complete medium and pre-incubated for 2 h. OTI CD8 + T cells were isolated from the spleens of OTI mice using EasySep Mouse CD8 + T Cell Isolation Kit (STEMCELL). Purified OTI CD8 + T cells were co-cultured with tumor cells at a ratio of 2:1, 0.5:1, 5:1 for 0–16 h in RPMI-1640 containing 50 U/ml IL-2 (PEPROTECH), 10% FBS, 10 mM HEPES, 100 mM NEAA and 50 mM b-Mercaptoethanol. Two hr before cell collection, Brefeldin A (BFA, BioLegend, 1:1000) was added to block cytokine secretion. T cells were washed and resuspended in staining buffer and stained with anti-CD8a (Clone: 53-6.7) and anti-IFN-γ, anti-GZMB antibodies for 30 min on ice. After a washing step, cells were performed intracellular staining as previously described in Flow Cytometry. For inhibitor treatment, Hep1-6-OVA cells were pre-treated with 10 μM or 50 μM Okanin for 48 h, and control cells were treated with DMSO. Before the co-culture was performed, pre-treated tumor cells were washed twice with DMEM and re-suspended in RPMI 1640. The processes of cell co-culture and staining were following the same steps above.

### T cell killing assay

2 mg/ml anti-CD3 (Biolegend) was coated to the culture dish and incubated at 4 °C overnight. Lymphocytes were obtained from lymph nodes of OTI mice. After the lymphocytes were resuspended, 0.5 mg/ml of anti-CD28 (Biolegend) was added to the anti-CD3-coated culture plate for 48 h. 1 × 10^5^ Hep-6-OVA cells were seeded into wells of a 96-well plate (NEST) with complete medium and the activated T cells were added. Dead tumor cells were counted by Trypan Blue staining after co-culture 6 hr.

### LC-MS analysis of metabolites

shNC or shSRSF1 Hep1-6-OVA cells were seeded in 100 mm dishes and cultured. When cells were approximately 60%-70% density, the cells were washed twice by PBS and cultured in medium with ^13^C-labeling glucose and 10% dialyzed FBS (Sigma) for 1 h. Cells were collected and washed with PBS and metabolites extracted using cold 80% methanol. Then, extracts were further spun at 13,300 rpm for 10 min and collected supernatants at 4 °C. The metabolites were subjected to vacuum freeze-drying. The Dionex Ultimate 3000 UPLC system was coupled to a TSQ Quantiva Ultra triple-quadrupole mass spectrometer (Thermo Fisher, CA), equipped with heated electrospray ionization (HESI) probe. Extracts were separated by a synergi Hydro-RP column (2.0 3 100 mm, 2.5 mm, phenomenex). A binary solvent system was used, in which mobile phase A consisted of 10 mM tributylamine adjusted with 15 mM acetic acid in water, and mobile phase B of methanol. This analysis used a 25 min gradient from 5% to 90% mobile B. Data acquired in selected reaction monitoring (SRM) for metabolites in positive-negative ion switching mode. The resolution for Q1 and Q3 are both 0.7 FWHM. The source voltage was 3500 v for positive and 2500 v for negative ion mode. The source parameters are as follows: capillary temperature: 350°C; heater temperature: 300°C; sheath gas flow rate: 35; auxiliary gas flow rate: 10. Tracefinder 3.2 (Thermo, USA) was applied for metabolite identification and peak integration.

### RNA isolation, quantitative reverse transcriptase PCR (qRT-PCR), RT-PCR

Total RNA from tumor cells was extracted using TRIzol reagent and cDNA was synthesized using EasyScript One-Step gDNA Removal and cDNA Synthesis SuperMix (TransGen). RT qPCR was performed using TransStart Top Green qPCR SuperMix (TransGen) on the StepOnePlus system (ABI). The qPCR conditions were 94 °C for 30 s; followed by 40 cycles of 94 °C for 5 s and 60 °C for 31 s. Amplification of specific transcripts was confirmed by melting curve profiles generated at the end of the PCR program. Expression levels of target genes were normalized to the expression of the ACT-b or GAPDH gene and were calculated based on the comparative cycle threshold method (2^-△△Ct^). RT-PCR was performed as described previously.^3^ RNA enrichment was measured by RT-PCR. Specific primers for RT-PCR were synthesized by BGI (Supplementary Table 7). In brief, the plasmids coding for HA-tagged SRSF1 and its domain deletion mutants as well as the empty vector were transiently transfected into HCC cells. IP was performed using the Magna RIP kit (Millipore). RNA enrichment was measured by RT-PCR. Specific primers for RT-PCR were synthesized by BGI (Supplementary Table 7).

### RNA-seq data analysis

Quality control on raw sequencing reads was performed by FastQC v0.11.8 (https://www.bioinformatics.babraham.ac.uk/projects/fastqc/). Adaptor and low-quality bases were trimmed by Trim Galore! version 0.6.0, a wrapper tool around Cutadapt and FastQC with command “trim_galore -q 20 –three_prime_clip_R1 3 –clip_R2 3 –phred33 –illumina –stringency 3 –paired –length 36”. Ribosomal RNA was removed by Bowtie2 version 2.3.5 and reads that did not align concordantly were retained and applied to quality control again with FastQC. Clean reads were then aligned to mm10 mouse genome with STAR version 2.7.0 f in default setting. Only alignments with mapping quality more than 20 were used for downstream analysis. Aligned reads were converted into bigwig format based on BPM normalization in 1 bp bin size with deepTools and visualized with Integrative Genomics Viewer (IGV). The featureCounts function of Rsubread v2.0.1 was utilized to count gene expression on transcriptome. Note that DNA contaminant was found in RNA-seq reads of co-cultured T cell samples, and this was corrected by subtracting the reads aligned to the reverse strand on exons. TPM normalization was performed as described above. Differential expressed analysis was performed by DESeq2 v1.26.0. Only genes with an average expression of at least 1 read among all samples were retained. Differentially expressed genes were defined as genes with adjust *p* < 0.01 and |log2(fold change) | <0.5.

### Western blot

Cell lysates and supernatants were resolved by electrophoresis, transferred to a polyvinylidene fluoride membrane, and probed with antibodies against β-tubulin (Cat# 2128, Cell Signaling Technology), beta-Actin antibody (Cat#ab6276, Abcam), NLK (Cat# ab97642, Abcam), Foxo1 (Cat# ab39670, Abcam), S6 (Cat# 2217, Cell Signaling Technology), p-S6 (Cat# 4858, Cell Signaling Technology), mTOR (Cat# ab134903, Abcam), PTEN (Cat# ab267787, Abcam), JunB (Cat# ab128878, Abcam), c-JUN (Cat# ab40766, Abcam), c-myc (Cat# ab32072, Abcam), CD38 (Cat# ab108403, Abcam), LDHA (Cat# ab52488, Abcam), PGAM1 (Cat# ab129191, Abcam), PGK1 (Cat# ab199438, Abcam), SRSF1 (Cat# ab129108, Abcam).

### Lentiviral-mediated gene transfer into tumor cells

Lentivirus-induced SRSF1-sh was performed in Hep1-6-OVA cells. Tumor cells in culture dishes (NEST) were co-transfected with 3 mg of either shNC or shSRSF1 PLKO.1 plasmid (Sigma). Virus-containing supernatants were gathered after 72 h, filtered using a 0.45 mm Filter (Thermo Fisher), and utilized to transfect tumor cells with 8 mg/ml Polybrene (Sigma). To select positively infected cells, tumor cells were administered with 5 mg/ml puromycin (Solarbio), while other cells received 4 mg/ml puromycin for 5 days. Stable tumor cell lines were subsequently maintained in puromycin.

### Construction of Srsf1-flox; Cd4/Cd8-cre mice

The Srsf1 conditional knockout mice were generated by CRISPR-Cas9 gene editing from Cyagen Biosciences. Cas9 protein, two gRNAs (gRNAB1: GCGCCTCGGTTTCCCGCTCCGGG, gRNAB2: CTAGTACTGCAACCTAATTTGGG) and a donor vector containing the two loxP sequences in intron 1 and intron 3 of mouse Srsf1 gene were injected into fertilized eggs. The embryos were transferred to recipient female mice to obtain F0 mice. The heterozygous Srsf1 loxP mice were intercrossed to obtain homozygous Srsf1 loxP mice. Then, homozygous Srsf1 loxp mice were crossed with CD4 or CD8 promoter-driven Cre recombinase mice (CD4-Cre mice and CD8-Cre mice) to generate Srsf1-T cell KO mice. The genotype of conditional knockout mice was confirmed by PCR using two pairs of primers (F1: - ACGTAAACGGCCACAAGTTC, R1: - TAGCCGTCGTAGTCGTAGCCGT; F2: ACTACTTCGGTCTTTGGGATGAA, R2: GGTAAGAGTCAGATCCAGAGTCCA) and sequencing.

### Animal studies

All research involving animals complied with protocols approved by the Fudan University Animal Care and Use Committee (2021-040). For hydrodynamic tail-vein injection (HDTVi), Vectors for HDTVi were prepared using the EndoFree-Maxi Kit (Qiagen) and resuspended in a sterile 0.9% NaCl solution/plasmid mix containing 10 μg of pX330-p53 (Addgene 59910) or pT3-N90-beta-catenin (Addgene 31785), and 10 μg of CMV-SB13 Transposase. CRISPR-Cas9 vector system carrying sgRNAs targeting Trp53 together with the Sleeping Beauty Transposon system overexpressing CTNNB1-N90 vector in sterile saline constituted a total volume of 10% of the mouse body weight were injected into the lateral tail vein of 6-week-old C57BL/6 J mice in 6–8 s. HDTVi-induced tumors were harvested 3–4 weeks after HDTVi. For the treatment regime, anti-PD-1 (5 mg/kg; Bio X Cell, West Lebanon, NH, USA) was i.p. injected every day or TN2008 (5 mg/kg for HCC model, 10 mg/kg for B16F10 model) every two days starting from day 7 after tumor cell implantation or plasmid injection.

Six- to eight-week-old male C57BL/6 J mice were treated under the following conditions. For subcutaneous HCC model, 1 × 10^6^ Hep1-6-OVA cells or B16F10 were resuspended in 50 μl growth-factor-reduced matrigel (#354234, Corning) and injected into the subcutaneous anesthetized 6-week-old male nude or C57BL/6 J mice. TN2008 (5 mg/kg; Targetmol, Shanghai, China) was i.p. injected every day starting at 6 days after cell inoculation.

For PDX model, tumors from HCC patients were minced into pieces (~3 × 3 × 3 mm) and transplanted to the subcutaneous tissue under aseptic conditions. When the subcutaneous tumor was 1 cm in diameter, it was minced into pieces and then subcutaneously implanted into the flanks of 4- to 5-week-old NOD/SCID mice. The P4 generation PDX model was used for the following experiment. After the tumor volume reached 100- 200 mm 3, the mice were randomly divided into the treatment or the control group. At the endpoint, the mice were euthanized with pentobarbital sodium (40 mg/kg), and the tumors were fixed in formalin and embedded in paraffin. Consecutive sections were prepared for each tumor tissue block and stained with H&E and IHC assays.

All mice were maintained under specific pathogen-free housing (~22 °C with ~40% humidity) on a 12 h dark/12 h light cycle. The following mice (at 6–8 week of age) were used for this study: male C57BL/6 J mice (The Jackson Laboratory); NSG mice (The Jackson Laboratory); C57BL/6-Tg (TcraTcrb)1100Mjb/J (OT-I) mice (The Jackson Laboratory); CD45.1 + CD45.2 + OT-I mice bred internally by crossing CD45.2 + OT-I mice and CD45.1 + C57BL/6 mice; and Srsf1^flox/flox^ mice. Srsf1^flox/flox^ mice were crossed with Cd4-Cre or Cd8-Cre mice to obtain specific Srsf1 deficiency in T cells (Srsf1fl/flCd4-Cre or Srsf1fl/flCd8-Cre mice). Hep1-6 cells (2 × 10^6) were inoculated subcutaneously into age- and sex-matched Srsf1^+/+^Cd4-Cre or Srsf1^fl/fl^Cd4-Cre or Ly5.2 + OT-I Srsf1^-/-^ mice (6–8 weeks). Tumor size was measured every 3 d using calipers fitted with a Vernier scale.

In vivo proliferation assays were performed using 5-week-old male BALB/c-nude, ll2rg^–/–^ NOD Scid mice or C57BL/6 mice (Chinese Academy of Sciences, Beijing, China). Briefly, 5 × 10^6^ tumor cells were implanted into the subcutaneous tissues. Tumor volumes were recorded every 2 days and were calculated with the formula V = (length × width^2^)/2. After 4 weeks, the mice were executed, and the tumors were excised. Other method details can be seen in supplementary materials.

## Supplementary information

Supplementary Materials

## Data Availability

The raw scRNA-seq data on human HCC we used to extend our analyses have been uploaded to the National Center for Biotechnology Information’s Gene Expression Omnibus database repository (https://www.ncbi.nlm.nih.gov/geo) under accession number GEO: GSE202642. We also examined publicly accessible scRNA-seq datasets from the Gene Expression Omnibus database, including GEO GSE100797, GSE123814, GSE221575, GSE178481, and GSE169246. Further RNA-seq raw data was available in SRA database: PRJNA1135531. Supplementary data for this study can be obtained by contacting the corresponding author.

## Code availability

The raw scRNA-seq data on human HCC we used to extend our analyses have been uploaded to the National Center for Biotechnology Information’s Gene Expression Omnibus database repository (https://www.ncbi.nlm.nih.gov/geo) under accession number GEO: GSE202642. We also examined publicly accessible scRNA-seq datasets from the Gene Expression Omnibus database, including GEO GSE100797, GSE123814, GSE221575, GSE178481, and GSE169246. Further RNA-seq raw data was available in SRA database: PRJNA1135531. Supplementary data for this study can be obtained by contacting the corresponding author.
