# Supplementary material for: Targeting SRSF1 improves cancer immunotherapy by dually acting on CD8+T and tumor cells
Source: Signal Transduct Target Ther. 2025 Jan 22;10:25. doi: 10.1038/s41392-024-02118-2 (PMC11751439; doi:10.1038/s41392-024-02118-2)
Supplement: Supplementary file 1 — Supplementary Materials [file 41392_2024_2118_MOESM1_ESM.docx]

Supplementary Materials for

Targeting SRSF1 improves cancer immunotherapy by dually acting on CD8^+^T and tumor cells

Gui-Qi Zhu^*^, Zheng Tang^*^, Tian-Hao Chu^*^, Biao Wang^*^, Shi-Ping Chen, Chen-Yang Tao, Jia-Liang Cai, Rui Yang, Wei-Feng Qu, Yi Wang, Qian-Fu Zhao, Run Huang, Meng-Xin Tian, Yuan Fang, Jun Gao, Xiao-Ling Wu, Jian Zhou, Wei-Ren Liu, Zhi Dai^#^, Ying-Hong Shi^#^, Jia Fan^#^

Correspondence to: fan.jia@zs-hospital.sh.cn (F.J); shi.yinghong@zs-hospital.sh.cn (S.Y.H); dai.zhi@zs-hospital.sh.cn (D.Z)

**This PDF file includes:**

Materials and Methods

Figures. S1 to S7

Tables S1 to S8

Materials and Methods

**Cell growth, proliferation assays**

Cell growth and the ability of anchorage-dependent growth (cell survival) was assessed by colony formation assays as described previously ^1,2^.

**Luciferase assays**

Cells were transfected as described in previous study^1,2^. After 24 hours in culture, cells were collected, lysed in passive lysis buffer (Promega), and luciferase activity was quantified on a luminometer using the dual-luciferase assay system (Promega) according to the manufacturer’s instructions.

**Rapamycin treatment**

Rapamycin was dissolved in DMSO and diluted in PBS. For in vitro culture treatment experiments, a final concentration of 1 nM or 10 nM rapamycin was added for the duration of cell cultures. For in vivo treatment experiments, rapamycin (2 mg/kg dissolved in DMSO, diluted in 150ul PBS) or PBS (equal volume DMSO in 150ul PBS) was administered to mice by intraperitoneal injection once every 2 days for 4 weeks. After 4 weeks of treatment, mice were euthanized, and spleen cells were analyzed by flow cytometry. Spleen cells were cultured with anti-CD3 (2 μg/mL) and anti-CD28 (2 μg/mL) for 24 hours.

**Proteins preparation**

The protein structure of SRSF1 was homologous modeled by SwissModel (https://swissmodel.expasy.org/) and optimized using Protein Preparation Wizard panel by correcting the bond order, adding hydrogen atoms, distributing charges, and predicting the protonation states (pH 7.0). The OPLS4 force field was used for constrained energy optimization to eliminate the atomic conflict on the structure with RMSD of heavy atoms converges to 0.3 Å, and the side chain position was optimized to obtain a reasonable side chain structure. Then amber18 was used for 100ns dynamic simulation to obtain a stable protein structure.

**Receptors for virtual screening**

The crystal structure of the RRM1 domain of mouse SRSF1 is currently unresolved. SWISS-MODEL (https://swissmodel.expasy.org/) was used for homology modeling. The protein structure of SRSF1 was optimized using *Protein Preparation Wizard* panel ^4^ by correcting the bond order, adding hydrogen atoms, distributing charges, and predicting the protonation states (pH 7.0). The OPLS4 force field was used for constrained energy optimization to eliminate the atomic conflict on the structure with RMSD of heavy atoms converges to 0.3 Å, and the side chain position was optimized to obtain a reasonable side chain structure. Sitemap was used to predict potential small molecule binding sites of proteins, and the most probable binding site was selected for further screening.

**Compound library preparation**

The compounds come from Topscience database(L1000) were processed with the *LigPrep* panel ^4^. Firstly, compounds were protonated and desalted with *Epik* program at pH7.0 ± 2.0 to generate tautomer and maintain the original atomic chirality. To ensure the conformational diversity of small molecules in the virtual screening process, each small molecule was generated up to 32 conformations^5^.

**Molecular docking and molecular dynamic analysis**

The GlideScore XP (Extra Precision) docking mode in Schrodinger software is used for molecular docking. Penalty points are given for inappropriate docking positions, and compensation points are given for appropriate hydrophobic and hydrogen bond interactions between ligands and proteins. Retaining up to 36 conformations using score function, then docking poses were further optimized using energy minimization, and the binding energies were calculated. Finally, the top 3 docking poses were retained. For other Settings, followed the default Schrodinger parameter Settings. DOCK 6.7 program was then utilized to conduct semi-flexible docking where 10000 different orientations were generated. The clustering analysis was performed to obtain the best scored poses, with an RMSD threshold of 2.0 Å. All molecular dynamics simulations were performed using GROMACS version 2016.4 ^5^ with AMBER ff99SB-ILDN force filed. CREB1-imperatorin complexes were centered in a cubic box of 10 Å solvated using TIP3P water model and SPC216 solvent configuration. MM/PBSA method was employed to calculate the binding free energies of ligand and protein as described previously ^6^.

**Interaction mode analysis**

Protein ligand interface fingerprint (PLIF) was used to calculate and analyze the binding sites and interaction types of compounds and RRM1 protein. The hydrogen bond, ionic bond, surface contact energy and other interactions were described and used to exclude the docking poses with low concurrency.

**Protein purification and surface plasmon resonance (SPR) assay**

Protein was purified by using Glutathione S-transferase (GST) tag protein purification kit (Beyotime Biotechnology). The pGEX-6P-1 plasmid expressing GST-tagged wild-type or mutant SRSF1 was transformed into E. coli BL21 star (DE3) cells, and isopropyl β-D-thiogalactopyranoside (IPTG) was added when bacteria culture was grown to a 600 nm (OD600) optical density of about 0.6. After obtaining SRSF1-GST protein, the GST tag was cut with PreScission Protease (Beyotime Biotechnology) according to manufacture’s protocol. SPR analysis was performed using the Biacore X100 system (GE Healthcare Life Sciences). Wide type SRSF1 and mutant SRSF1 protein was immobilized by amine coupling onto a CM7 chip (GE Healthcare Life Sciences) as described previously ^7^. Impetration in PBS buffer was added with a speed of 30 μL/min for 90 s, and dissociation was evaluated by passing HBS buffer alone over the chip at 30 μl/min for 10 min.

**Measurements of glucose uptake and metabolites**

The fluorescent 2-DG analog 2-NBDG was used to measure glucose uptake. The cells were incubated with 2-NBDG (10 μM) for 1 h, washed twice by PBS, and analyzed by flow cytometry (BD Biosciences, Franklin Lakes, NJ). For assessment of lactate production, the cells were resuspended in DMEM without pyruvate, plated in 96-well plates (8 ×10^4^ cells per well), and allowed to grow for 6 h. The cell media was collected and diluted 1:6 in lactate assay buffer. The amount of lactate in the media was then measured using the lactate colorimetric assay kit (BioVision) according to the manufacturer’s instructions.

**Flow cytometry**

Briefly, four different group tumor tissues were digested at 37°C for 30 min with 1 mg/ml Collagenase D and 0.1 mg/ml DNase I (Roche). Digestion was stopped by EDTA and cells were filtrated through 70 mm cell strainers and washed twice with PBS containing 1 mM EDTA and 2% FBS (staining buffer). Cells were re-suspended in the staining buffer and stained with following antibodies on ice for 30 min: anti-CD45, anti-CD8, anti-IFNg, anti-Granzyme B, anti-CD38, anti-CD11b, anti-CD33, anti-CD44, anti-CD62L, anti-FOXP3, anti-CD25, anti-F4/80, Ly6C were purchased from BioLegend. For intracellular staining, cells were fixed with fixation buffer (Biolegend) on ice for 15 min, and then washed twice with Intracellular Staining Permeabilization Wash Buffer (Biolegend). Antibodies against IFN-g (Clone XMG1.2) and Granzyme B (Clone: QA16A02) were added and incubated for 1 hr on ice. The cytokine producing cells were determined by flow cytometry. The flow cytometry data were collected on Fortessa (BD) and analyzed by FlowJo (Tree Star). For cell sorting, CD8+ T cells that were co-cultured with tumor cells for 6 hr were collected and washed with culture medium. Re-suspended cells were stained with anti-CD8a antibodies (Clone: 53-6.7) for 30 min on ice. After a washing step, cells were sorted on a BD FACS AriaIII (BD) and lysed in the buffer RLT plus (QIAGEN).

**CyTOF analysis of immune cells**

CyTOF analyses were performed by PLTTech Inc. (Hangzhou, China) according to the previously described protocol ^8^. In brief, tumour tissue was dissociated into single cells with DNAase, collagenase IV and hyaluronidase (Sigma-Aldrich, Saint Louis, MO, USA). Immune cells were enriched using Percoll density gradient media (Sigma-Aldrich), and red blood cells were removed using ACK Lysing Buffer (Sigma-Aldrich). Qualified samples were blocked and stained for 30 min with a surface antibody mix panel developed in-house (Supplementary Table 8), followed by fixation overnight. Permeabilization buffer was applied, and the cells were incubated in an intracellular antibody mix. The cells were rinsed, and the signals were detected using a CyTOF system (Helios, Fluidigm, South San Francisco, CA, USA). The types of immune cells were identified via nonlinear dimensionality reduction [t-distributed stochastic neighbor embedding (tSNE)].

**References**

1 Zhu, G. Q. *et al.* CD36(+) cancer-associated fibroblasts provide immunosuppressive microenvironment for hepatocellular carcinoma via secretion of macrophage migration inhibitory factor. *Cell Discov* **9**, 25, (2023).

2 Zhu, G. Q. *et al.* Targeting HNRNPM Inhibits Cancer Stemness and Enhances Antitumor Immunity in Wnt-activated Hepatocellular Carcinoma. *Cell Mol Gastroenterol Hepatol* **13**, 1413-1447, (2022).

3 Zhou, X. *et al.* Transcriptome analysis of alternative splicing events regulated by SRSF10 reveals position-dependent splicing modulation. *Nucleic Acids Res* **42**, 4019-4030, (2014).

4 Zheng, X., Zheng, T., Liao, Y. & Luo, L. Identification of Potential Inhibitors of MurD Enzyme of Staphylococcus aureus from a Marine Natural Product Library. *Molecules* **26**, 6426, (2021).

5 Pronk, S. *et al.* GROMACS 4.5: a high-throughput and highly parallel open source molecular simulation toolkit. *Bioinformatics* **29**, 845-854, (2013).

6 Kumari, R., Kumar, R. & Lynn, A. g_mmpbsa--a GROMACS tool for high-throughput MM-PBSA calculations. *J Chem Inf Model* **54**, 1951-1962, (2014).

7 Pettersen, E. F. *et al.* UCSF Chimera--a visualization system for exploratory research and analysis. *J Comput Chem* **25**, 1605-1612, (2004).

8 Han, G. *et al.* Metal-isotope-tagged monoclonal antibodies for high-dimensional mass cytometry. *Nat Protoc* **13**, 2121-2148, (2018).

**Figure. S1.** High levels of SRSF1 were associated with exhausted CD8+T cells and anti-PD-1 non-responsive CD8+T cells.

(a) Barplots showing SRSF1 were highly expressed in tumor tissues in multiple cancers from TCGA databases. (b) IHC staining showing SRSF1 expressed more in HCC tissues and high expression of SRSF1 indicated HCC poor prognosis. (c-d) Correlation between SRSF1 expression and exhausted, cytotoxic and memory CD8+T cells in multiple cancers from ESTIMATE algorithm. (e) Correlation between SRSF1 expression and exhausted CD8+T signatures in multiple cancers from TCGA databases. (f) Violin plots showing SRSF1 expression in CD8+T cells between CRC or adjacent tissues from GEO database (GSE221575). (g) Barplots showing SRSF1 expression in CD8+T cells between RCC or adjacent tissues from GEO database (GSE178481). (h) TP53 and CTNNB1 mutation profile in HCC from TCGA database. (i) SRSF1 expression in CD8+T cells between nonresponsive and responsive groups from our HCC patients. (j) The expression of SRSF1 and exhausted CD8+T marker genes in basal cell carcinoma from GEO database (GSE123814), red indicates upregulated and blue indicates downregulated. (k) SRSF1 expression in CD8+T cells between nonresponsive and responsive groups from basal cell carcinoma patients. Data shown as mean ± S.E.M. Statistical significance was determined by two-tailed unpaired t test and two-way ANOVA. *p < 0.05, **p < 0.01, ***p < 0.001, ****p < 0.0001.

**
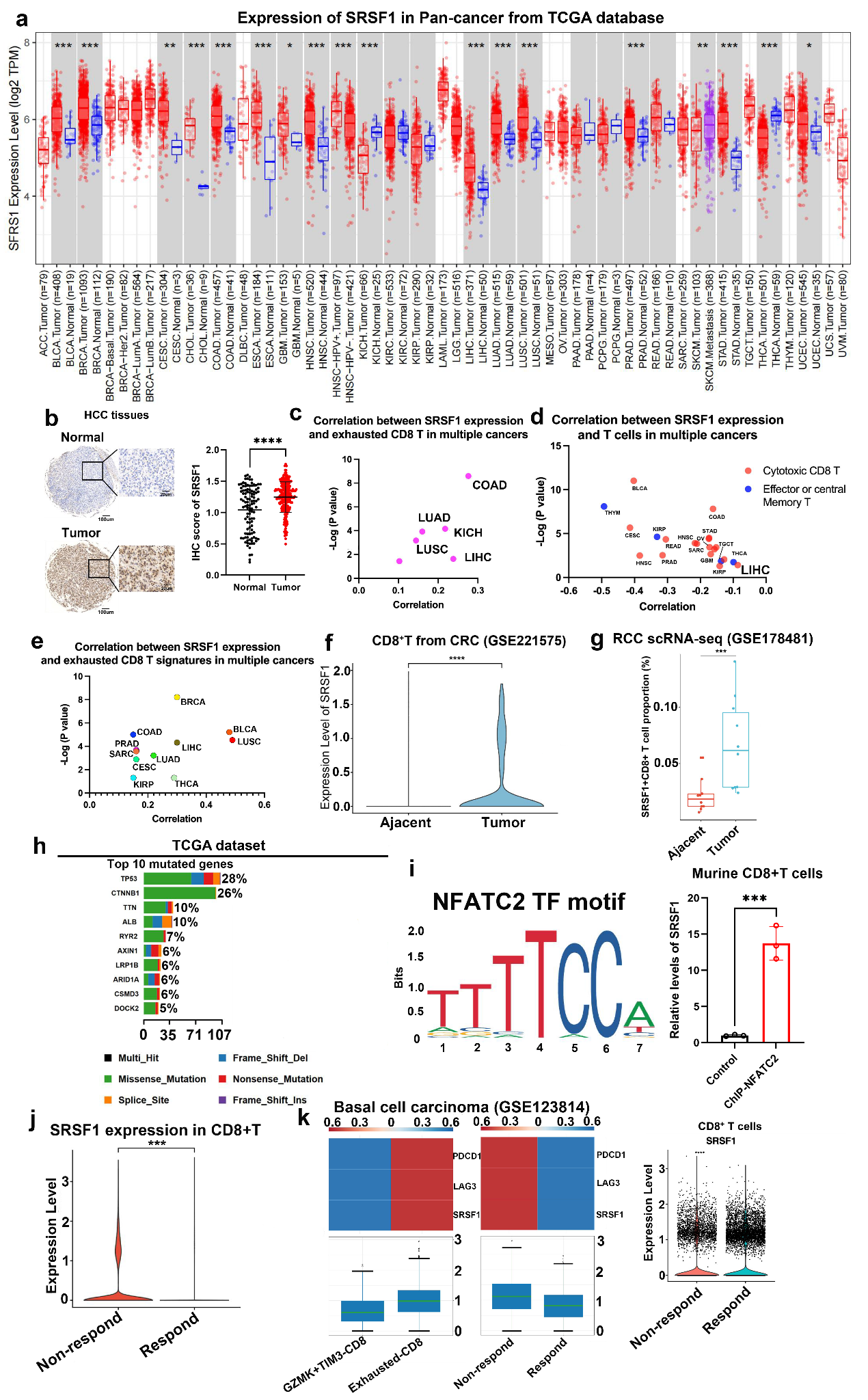
**

**Figure. S2.** SRSF1 knockout in CD8+ T cell enhances anti-tumor effects and adoptive T cell therapy.

(a) The mice body and spleen characterization in Srsf1^fl/fl^ and Srsf1^fl/fl^;Cd4^cre^ mice groups. (b) The proportion of CD44+CD62LlowCD4+T cells from spleen between Srsf1^fl/fl^ and Srsf1^fl/fl^; Cd4^cre^ mice groups. (c) The schematic diagram of HCC mice model establishment between two groups. (d) Tumor number in Srsf1^fl/fl^Cd8-cre and Srsf1^+/+^Cd8-cre mice groups (n=6 per group). (e) The schematic diagram for HCC tumor model by adoptive T cell therapy. (f) SRSF1 knockout in CD8+ T cell enhances their adoptive T cell therapy, i.v. 1x10^6 CD8+T cells and the survival analysis for three groups. (g) SRSF1 knockout in CD8+ T cell enhances their infiltration. (h-i) SRSF1 knockout in CD8+ T cell enhances their cytotoxicity in vivo. Data shown as mean ± S.E.M. Statistical significance was determined by two-tailed unpaired t test, two-way ANOVA and log rank test. *p < 0.05, **p < 0.01, ***p < 0.001, ****p < 0.0001.


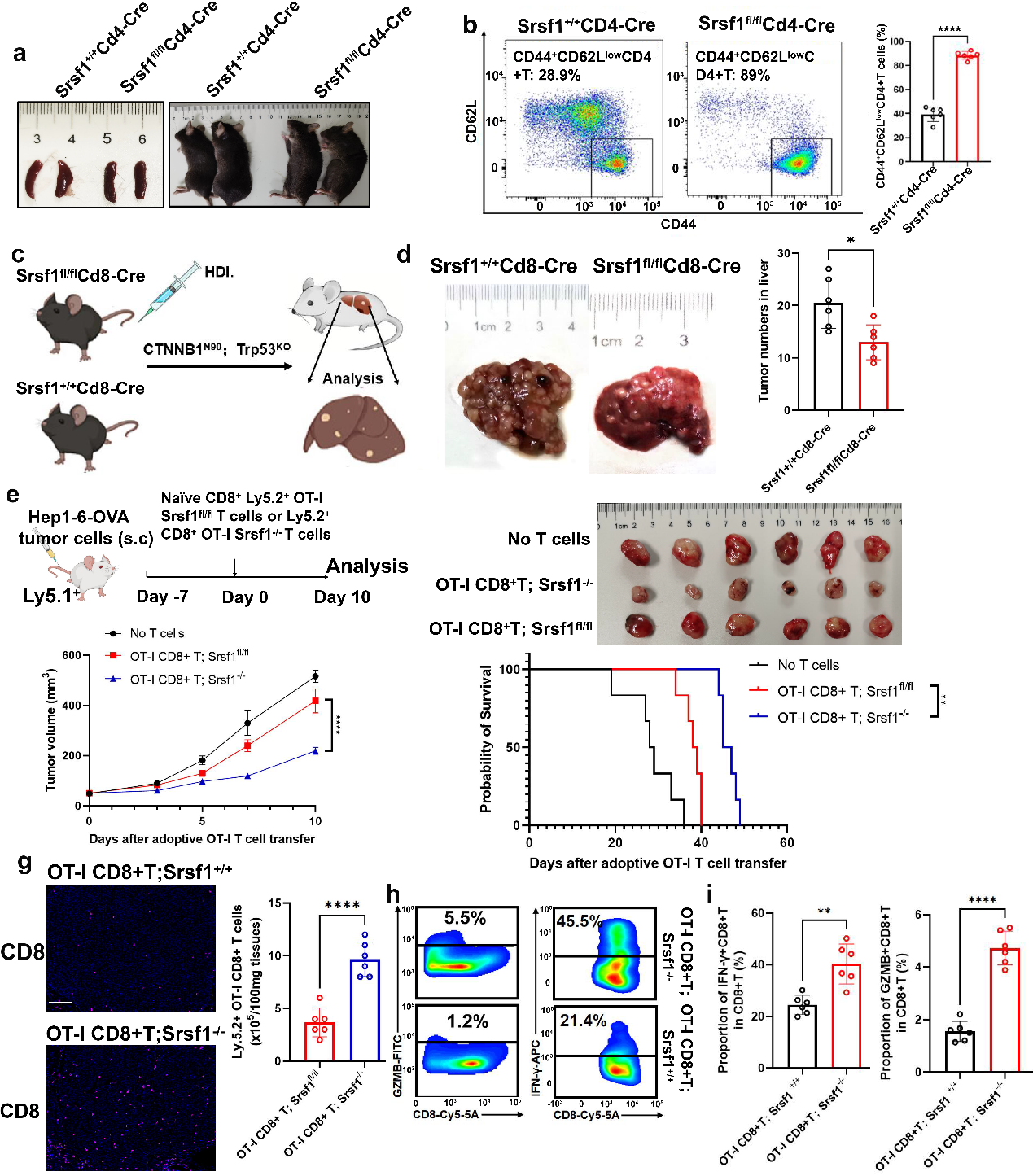


**Figure. S3.** SRSF1 inhibition increased CD8^+^T cell infiltration and functionality. (a) SRSF1 was knockdown when transfected with SRSF1-sh lentivirus in Hep1-6 cells. (b) SRSF1 knockdown decreased tumor growth in C57BL/6J mice. (c) SRSF1 knockdown decreased tumor growth in nude mice. (d) Relative tumor growth change between nude mice and C57BL/6J mice. (e) SRSF1 knockdown increased CD8+T cell infiltrations. (f) Tumor inhibition by SRSF1 knockdown required the effects of CD8+T cell. (g) SRSF1 knockdown increased CD8+T cell effector function. (h) Scatter plot showing SRSF1 knockdown increased CD8+T cell cytotoxicity. (i) GSEA analysis showed SRSF1 knockdown increased CD8+T cell cytotoxicity. (j) T cell co-culture assays showed SRSF1 knockdown increased CD8+T cell cytotoxicity. (k) SRSF1 knockdown increased CD8+T cell effector function. Data shown as mean ± S.E.M. Statistical significance was determined by two-tailed unpaired t test, two-way ANOVA. *p < 0.05, **p < 0.01, ***p < 0.001, ****p < 0.0001.

**
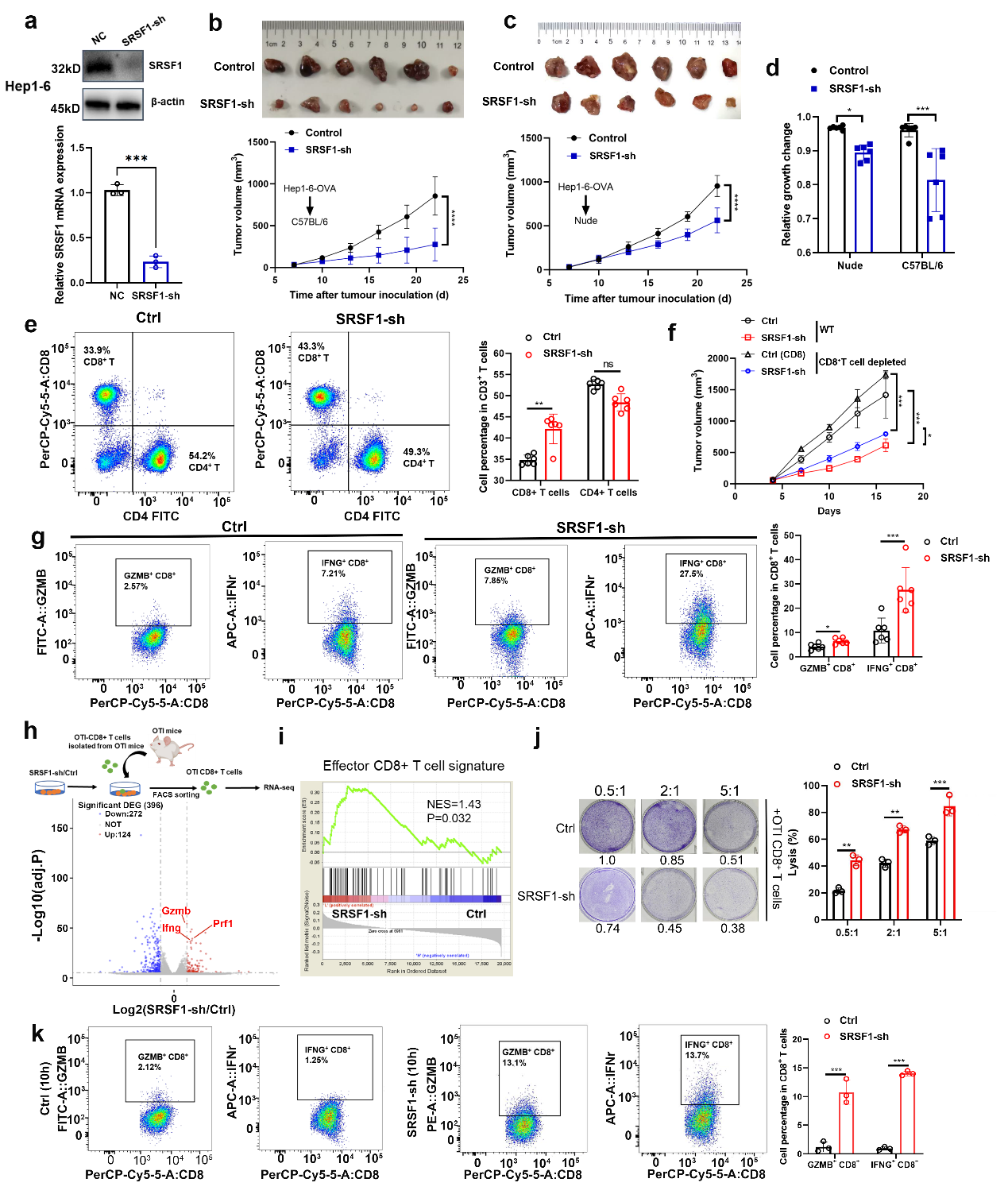
**

**Figure. S4.** SRSF1 depletion inhibited glycolysis in HCC by inhibiting bZIP and c-myc transcription factors. (a-b) ECAR analysis showed SRSF1 knockdown decreased the glycolysis of tumor cells. (c) Untargeted metabolomics showed SRSF1 knockdown decreased central carbon metabolism in cancer. (d) Targeted metabolomics SRSF1 knockdown decreased glycolysis-related metabolites in HCC. (e) SRSF1 knockdown inhibited glucose uptake in HCC tumor model from C57BL/6J mice. (f) HCC patients with SRSF1 high expression had higher glucose uptake rate and lactate levels. (g) Heatmap showed the expression of glycolytic pathway genes when SRSF1 was knockdown. (h-i) Schematic diagram of specific binding sites of bZIP and c-myc transcription factors to the promoter of glycolytic genes. (j) bZIP transcription factors transcriptionally upregulated the expression of glycolytic genes. (k) c-myc transcription factor transcriptionally upregulated the expression of glycolytic genes. (l) Western Blot assays showed the downregulated expression of glycolytic genes by SRSF1 knockdown can be rescued by overexpression of transcription factors. Data shown as mean ± S.E.M. Statistical significance was determined by two-tailed unpaired t test and two-way ANOVA. *p < 0.05, **p < 0.01, ***p < 0.001, ****p < 0.0001.

**
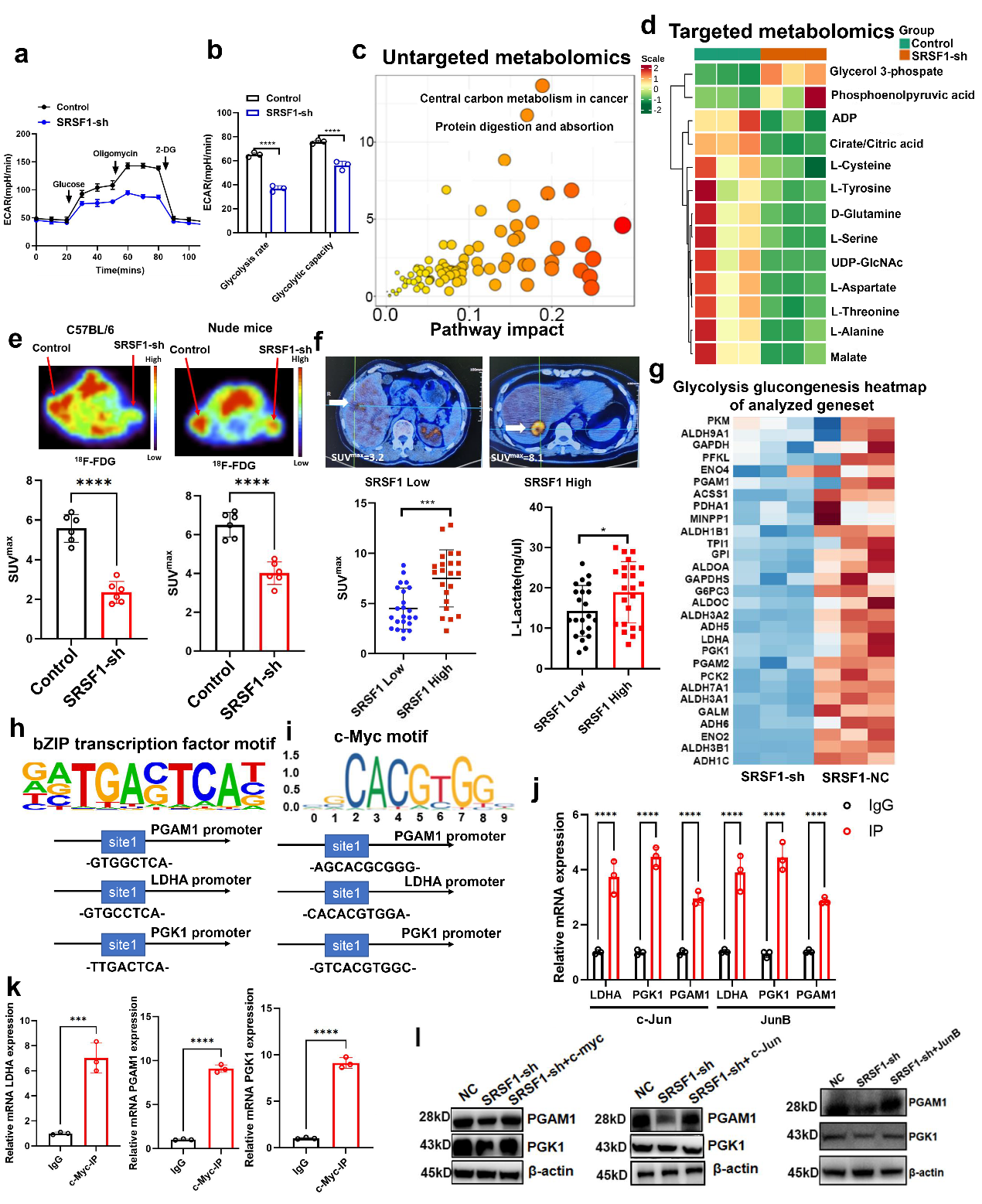
**

**Figure. S5.** The role of SRSF1 inhibitor TN2008 on tumor cell and CD8+ cells. (a) The structure of SRSF1-RRM1 (Top left), overview structural complex of SRSF1 bound with TN2008 (Top right and bottom left) and structure assessment of SRSF1 by homology modelling (Bottom right). The conformation in the dark green area of the figure (Bottom right) is the stereochemistry allowed region, which can exist stably. The light green colored region is the maximum allowable zone, where the conformation can exist in stereochemistry but is unstable. Blank areas are not allowed in stereochemistry and cannot exist. This can be seen that more than 90% of the amino acids in the model can be within the maximum allowable range, so it is believed that the residue conformation of the model conforms to the rules of stereochemistry and can be used for subsequent virtual screening. (b) The SPR assays showed TN2008 specifically bound with SRSF1 and TN2008 cannot specifically bound with other SRSF members (down panel). (c) The schematic diagram of TN2008 co-cultured with OTI CD8+T cells from mice spleen. (d) The effect of different doses of TN2008 on CD8+T cells. (e) The percentage of effector CD8+T cell co-cultured with tumor cells pretreated with TN2008. (f-h) The effect of different doses of TN2008 on subcutaneous tumors of HCC in mice. (i) Mice body weights between different groups. Data shown as mean ± S.E.M. Statistical significance was determined by two-tailed unpaired t test and two-way ANOVA. *p < 0.05, **p < 0.01, ***p < 0.001, ****p < 0.0001.

**
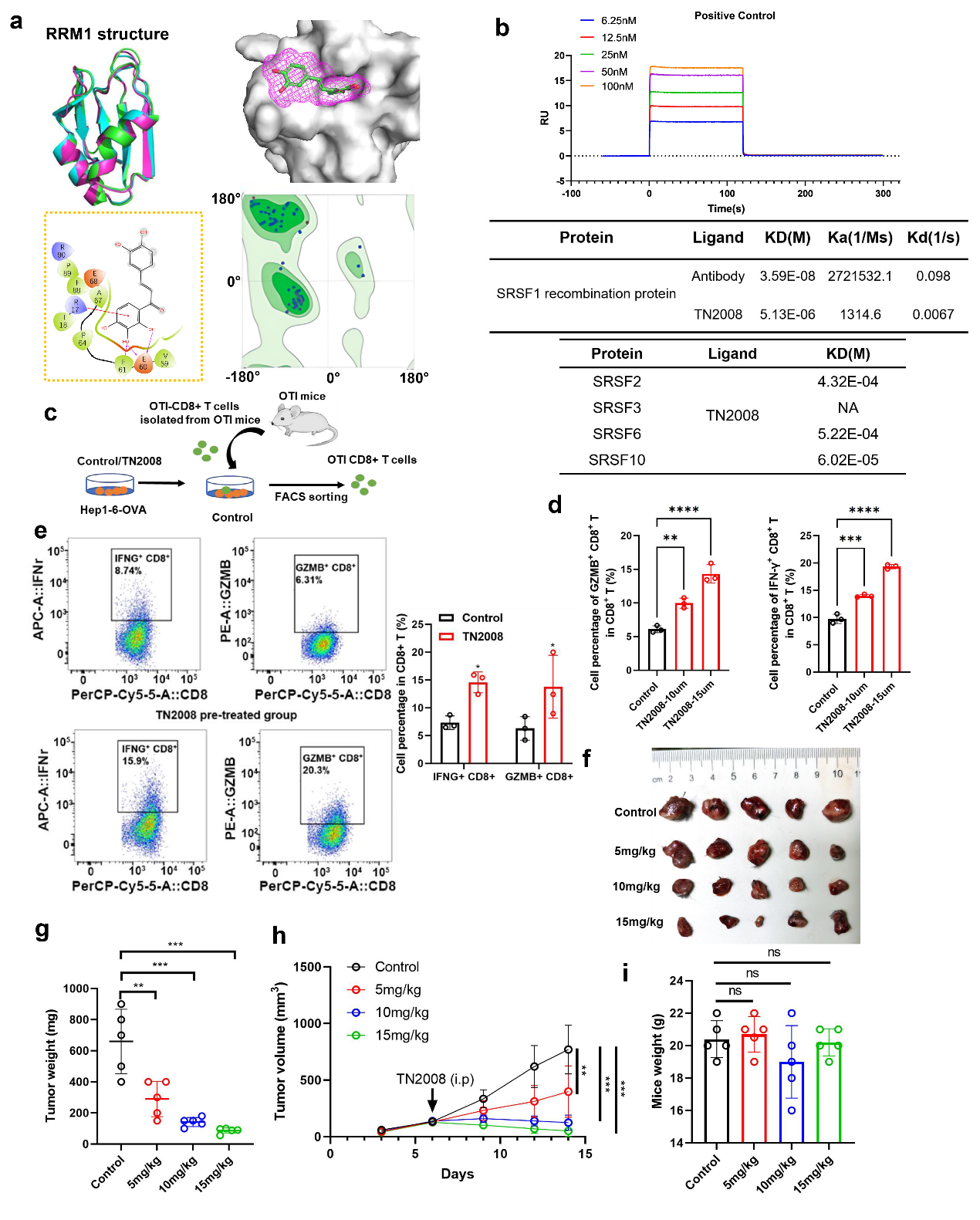
**

**Figure. S6.** The role of TN2008 in mice tumor models. (a) The growth curve of subcutaneous tumors for different groups in each mouse over time. (b) Different immune cells from tumor microenvironment in different groups from spontaneous HCC tumor model. (c) The density plot shows the expression of CD86 in three groups from spontaneously HCC tumor model. (d) The density plot shows the expression of KI-67 in three groups from spontaneously HCC tumor model. (e) The expression of CD38 marker among three groups. (f) TN2008 sythergized with anti-PD-1 in PD-1 resistant murine melanoma models. (g) The tumor growth between four different groups. (h) Survival analysis between different groups. (i-j) The percentage of effector CD8+T cells (i) and Tregs (j) between four different groups. (k) The western blot showed PTEN and mTOR protein levels when SRSF1 was silenced in tumor cells. (l) The intersect transcriptomic results between CD8+T-SRSF1-KO and tumor cell-SRSF1-sh groups. Data shown as mean ± S.E.M. Statistical significance was determined by two-tailed unpaired test, two-way ANOVA and log-rank test. *p < 0.05, **p < 0.01, ***p < 0.001, ****p < 0.0001.

**
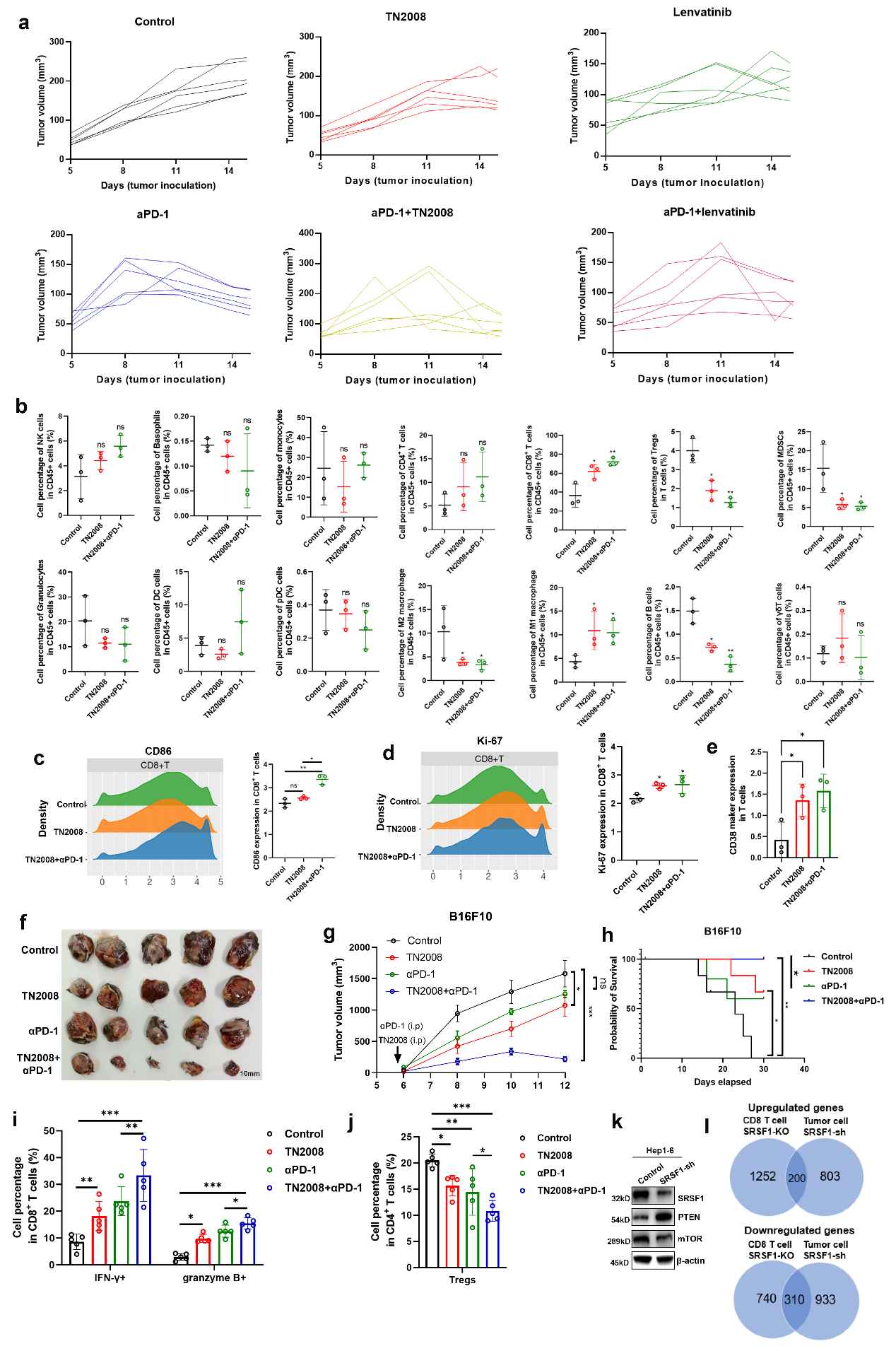
**

**Figure. S7.** The graphical abstract for this study. The figure was obtained by using Biorender (https://app.biorender.com/).


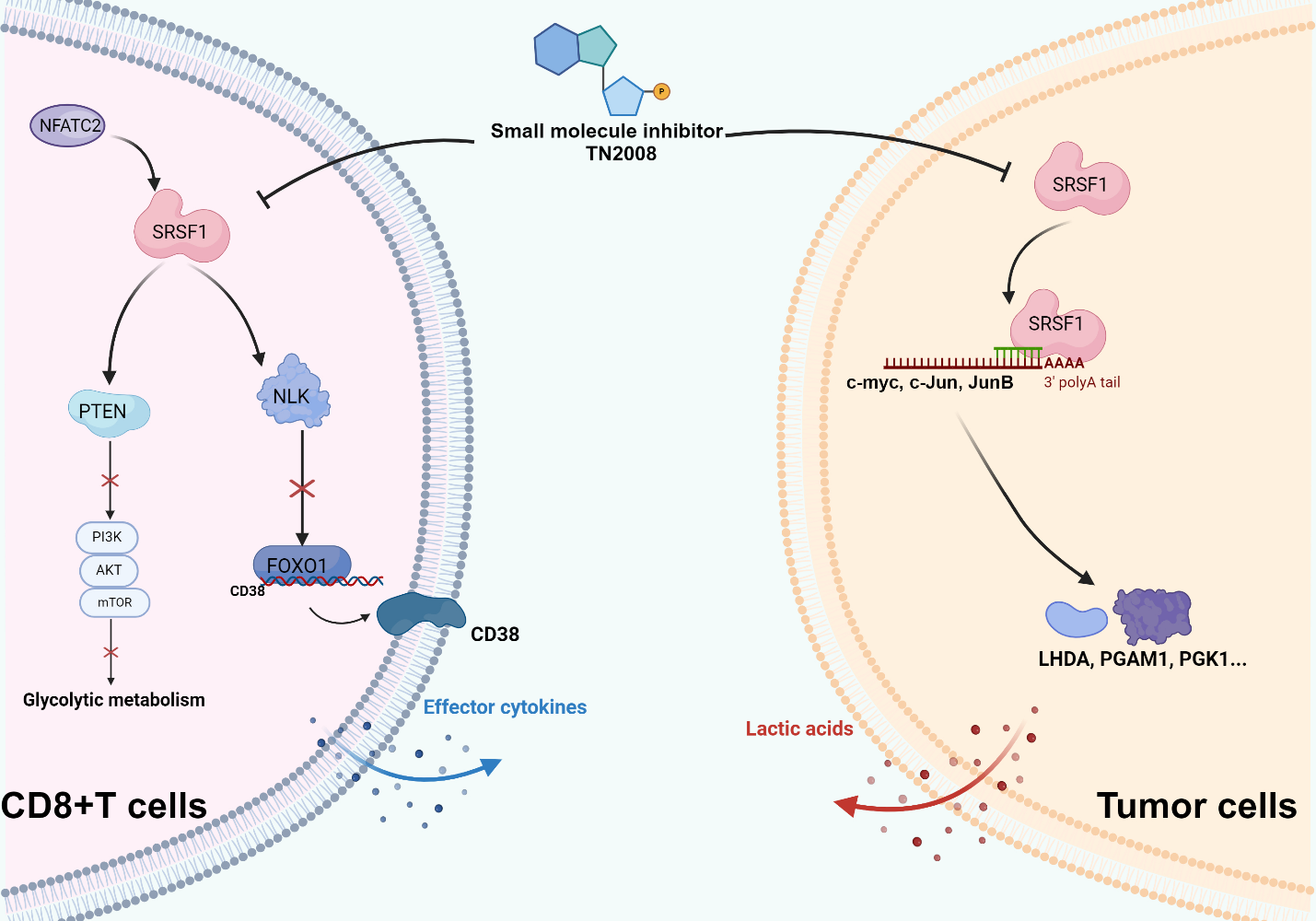


| **Table S1.** The baseline characteristics of 7 HBV-related HCC patients. | | | | | | | | | | |
| --- | --- | --- | --- | --- | --- | --- | --- | --- | --- | --- |
| Sample | Age | Gender | HBsAg | Diagnosis | Tumor number | Tumor diameter (cm) | Tumor grade | Tumor encapsulation | Microvascular invasion | Macrovascular invasion |
| T1 | 66 | Male | Positive | HCC | 2 | 17*16/14*12 | III | Yes | Yes | No |
| T2 | 54 | Male | Positive | HCC | 2 | 10*8/3.7*3 | II | No | Yes | No |
| T3 | 58 | Male | Positive | HCC | 1 | 3.9*3.2 | III | Yes | No | No |
| T4 | 64 | Male | Positive | HCC | 2 | 8*4.5/3.8*2.5 | II | Yes | No | No |
| T5 | 77 | Male | Positive | HCC | 2 | 7*6/4.5*3 | II | No | Yes | No |
| T6 | 41 | Male | Positive | HCC | 1 | 3.5*3.5 | II | No | Yes | No |
| T7 | 54 | Male | Positive | HCC | 1 | 3.5*2.9 | II | Yes | Yes | No |

**Table S2.** Clinical summary of HCC patients treated with immune checkpoint blockade.

| Patient ID | Age | Gender | HBV | Tumor diameter (cm) | Tumor grade | Treatment response^a^ (R/NR) |  |
| --- | --- | --- | --- | --- | --- | --- | --- |
| neoP1 | 64 | Male | Positive | 1.7*1.7 | II | R |  |
| neoP2 | 48 | Male | Positive | 2.5*2.1/1.5*1 | II | NR |  |
| neoP3 | 62 | Male | Positive | 3*2.5 | III | NR |  |
| neoP4 | 73 | Male | Negative | 6*5.5 | II | R |  |
| ^a^ The response annotation was determined based on the clinical-radiological best response. Stable and progressive disease was classified as non-responsive, while partial and complete response was classified as responsive.  Abbreviations: HCC, hepatocellular carcinoma; HBV, Hepatitis B virus; R, responsive; NR, non-responsive; scRNA-seq, single-cell RNA sequencing; IHC; immunohistochemistry. | | | | | | | |

| **Table S3.** List of cell type-specific marker genes used for cell annotation | | | | | | | | | | |
| --- | --- | --- | --- | --- | --- | --- | --- | --- | --- | --- |
| **CD8+ T cells** | **CD4+ T cells** | **NK cells** | **NKT cells** | **Effector CD8+ T cells** | **Exhausted CD8+ T cells** | **Naïve T cells** | **MAIT** | **Early effector memory T cells (CCR6+ CD8T)** | **CXCL13+ CD8T** | **Terminal effector T (PRF1+ GZMB+ CD8T)** |
| CD8A | CD4 | GNLY | CD3D | IFNG | TIGIT | TCF7 | KLRB1 | TNFAIP3 | CXCL13 | GNLY |
| CD8B | IL7R | NKG7 | CD3E | TNF | PDCD1 | LEF1 | SLC4A10 | CCL20 | CTLA4 | GZMB |
| CD3D | TRBC2 | CD247 | GNLY | GZMB | CD274 | CCR7 | NCR3 | NFKBIA | RGS1 | FGFBP2 |
| CD3E |  | FCER1G | NKG7 | GZMA | CTLA4 |  | IL23R | FOSB | MIR155HG | CX3CR1 |
|  |  | KLRG1 | CD247 | GZMK | LAG3 |  | LST1 | FOS | DUSP4 | GZMH |
|  |  | TYROBP | FCER1G | GZMH | HAVCR2 |  |  | CEBPD | PDCD1 | NKG7 |
|  |  |  | KLRG1 | PRF1 | CD244 |  |  | DUSP1 | ITM2A | LGALS1 |
|  |  |  | TYROBP |  | CD160 |  |  | PPP1R15A | GEM | ADGRG1 |
|  |  |  |  |  |  |  |  | NR4A1 | CCL4L2 | EFHD2 |
|  |  |  |  |  |  |  |  | CD69 | TNFRSF9 | KLF2 |

| **Table S4.** The results of genes binding to SRSF1 by RIP experiment. | | |
| --- | --- | --- |
| Gene | SRSF1_NC_input | SRSF1_NC_IP |
| JUNB | 13.8 | 45.3 |
| MYC | 13.7 | 39.5 |
| c-JUN | 33.7 | 88.4 |
| ZNF354B | 0.1 | 2.1 |
| HMGB2P1 | 2.2 | 33.3 |
| LRRFIP1P1 | 2.8 | 42.4 |
| SDHAP3 | 0.4 | 5.8 |
| TRIM52 | 0.6 | 8.3 |
| TCHH | 2.6 | 38.1 |
| RP1-163M9.8 | 1975.5 | 20939.3 |
| UBE2Q2P1 | 0.6 | 6.4 |
| RNU1-3 | 13286.8 | 139461.6 |
| ANKRD24 | 0.2 | 2.1 |
| RNVU1-19 | 16.4 | 169.5 |
| BMS1P1 | 0.2 | 2.1 |
| RNVU1-15 | 24.8 | 253.9 |
| ANKS3 | 0.2 | 2.4 |
| RNU1-1 | 21279.4 | 212383.6 |
| MIR194-2HG | 0.1 | 1.2 |
| C19orf57 | 0.1 | 1.2 |
| CRELD1 | 0.2 | 2.4 |
| LINC01239 | 0.2 | 1.8 |
| CEACAM16 | 0.3 | 2.7 |
| VN1R21P | 0.6 | 5.9 |
| CTD-2165H16.4 | 0.2 | 1.6 |
| CELF6 | 0.1 | 1.3 |
| BRDTP1 | 0.7 | 6.7 |
| OR7E126P | 0.5 | 5.1 |
| PYROXD2 | 0.4 | 3.5 |
| FNBP1P1 | 0.5 | 4.5 |
| SLC25A27 | 0.3 | 2.8 |
| GOLGA6L17P | 0.9 | 8.8 |
| VAMP1 | 0.5 | 5.1 |
| ZNF860 | 0.1 | 1.4 |
| CTD-2639E6.9 | 1 | 9.6 |
| AC074117.10 | 0.8 | 8 |
| RP11-629O1.2 | 0.7 | 6.7 |
| ABHD16B | 0.3 | 3 |
| RYR2 | 0.2 | 1.5 |
| GNB3 | 0.1 | 1.3 |
| APOBEC3D | 0.2 | 1.8 |
| FXYD1 | 0.2 | 2.4 |
| PTMAP8 | 1.3 | 13.2 |
| GOLGA6L5P | 1.9 | 18.3 |
| FTX_3 | 9.3 | 89.9 |
| HOXA2 | 0.6 | 5.4 |
| RP11-5A19.5 | 0.7 | 6.6 |
| LINC01348 | 0.4 | 4.1 |
| RP11-280G9.1 | 1.8 | 17.6 |
| HTATSF1P2 | 2.9 | 27.5 |
| AC137932.4 | 0.5 | 4.7 |
| ZNF767P | 0.5 | 4.3 |
| RP11-158M2.2 | 1.6 | 15.3 |
| JAKMIP3 | 0.9 | 8.5 |
| ZNF761 | 0.3 | 2.7 |
| WEE2-AS1 | 0.8 | 7.3 |
| RP11-6N17.10 | 2.6 | 24.2 |
| AC009237.8 | 0.4 | 3.4 |
| RP3-340N1.2 | 0.7 | 6.4 |
| CTC-338M12.7 | 3.2 | 30 |
| PPFIA4 | 0.4 | 3.9 |
| ZNF112 | 0.9 | 8.7 |
| RNU1-18P | 80.5 | 736.5 |
| SPDYE5 | 0.1 | 1.2 |
| CHMP4BP1 | 1.5 | 14.1 |
| OR7E128P | 0.4 | 4.1 |
| LINC01268 | 0.2 | 1.5 |
| RP11-631M6.2 | 0.6 | 5.6 |
| RP11-295D4.3 | 0.2 | 2.1 |
| GIP | 0.6 | 5.8 |
| CCDC146 | 0.1 | 0.9 |
| NBPF3 | 0.1 | 0.6 |
| CTB-52I2.4 | 0.5 | 4.8 |
| STX1B | 0.1 | 0.8 |
| HNRNPA1P39 | 0.9 | 8.7 |
| PSORS1C1 | 0.1 | 0.7 |
| ST18 | 0 | 0.3 |
| GPRC5D | 0.4 | 3.7 |
| RP11-163E9.1 | 1.3 | 12.3 |
| AP4B1-AS1 | 0.3 | 2.4 |
| CALM2P2 | 1 | 9.4 |
| CTB-31O20.4 | 0.2 | 1.7 |
| KRT8P32 | 0.3 | 2.8 |
| CTD-2574D22.3 | 0.2 | 1.9 |
| C17orf107 | 0.1 | 1.2 |
| CCDC183-AS1 | 3.2 | 29.1 |
| KIF21B | 0.1 | 1.3 |
| RNVU1-18 | 130.5 | 1160 |
| BX470102.3 | 2.6 | 22.7 |
| HOXB6 | 1 | 9.3 |
| AC007238.1 | 0.5 | 4.6 |
| RP11-927P21.5 | 1.9 | 16.1 |
| PVT1_3 | 9.6 | 83.4 |
| BDH1 | 0.1 | 0.9 |
| RNU1-82P | 16.6 | 144.1 |
| RP11-536K17.1 | 6.6 | 56.4 |
| GS1-124K5.12 | 2.4 | 20.2 |
| BMS1P10 | 3.3 | 27.6 |

| **Table S5.** The spatial information of each atom for SRSF1-RRM1 domain by homology modelling. | | | | | |
| --- | --- | --- | --- | --- | --- |
| **Atoms** | **Radical group** | **amino acid** | **Spatial value1** | **Spatial value2** | **Spatial value3** |
| ATOM1 | N | ARG | 33.605 | 42.786 | 42.198 |
| ATOM2 | H1 | ARG | 33.773 | 43.566 | 41.579 |
| ATOM3 | H2 | ARG | 33.05 | 43.145 | 42.962 |
| ATOM4 | H3 | ARG | 34.428 | 42.357 | 42.597 |
| ATOM5 | CA | ARG | 32.734 | 41.717 | 41.642 |
| ATOM6 | HA | ARG | 31.739 | 42.111 | 41.436 |
| ATOM7 | CB | ARG | 33.259 | 41.276 | 40.278 |
| ATOM8 | HB2 | ARG | 32.432 | 40.826 | 39.728 |
| ATOM9 | HB3 | ARG | 33.723 | 42.121 | 39.77 |
| ATOM10 | CG | ARG | 34.337 | 40.092 | 40.31 |
| ATOM11 | HG2 | ARG | 35.12 | 40.374 | 41.013 |
| ATOM12 | HG3 | ARG | 33.878 | 39.159 | 40.639 |
| ATOM13 | CD | ARG | 34.892 | 39.927 | 38.84 |
| ATOM14 | HD2 | ARG | 34.089 | 39.621 | 38.169 |
| ATOM15 | HD3 | ARG | 35.345 | 40.847 | 38.474 |
| ATOM16 | NE | ARG | 35.931 | 38.896 | 38.837 |
| ATOM17 | HE | ARG | 36.39 | 38.634 | 39.698 |
| ATOM18 | CZ | ARG | 36.465 | 38.376 | 37.748 |
| ATOM19 | NH1 | ARG | 35.98 | 38.771 | 36.592 |
| ATOM20 | HH11 | ARG | 35.32 | 39.535 | 36.61 |
| ATOM21 | HH12 | ARG | 36.303 | 38.454 | 35.689 |
| ATOM22 | NH2 | ARG | 37.318 | 37.422 | 37.877 |
| ATOM23 | HH21 | ARG | 37.505 | 37.133 | 38.826 |
| ATOM24 | HH22 | ARG | 37.786 | 36.988 | 37.094 |
| ATOM25 | C | ARG | 32.556 | 40.525 | 42.61 |
| ATOM26 | O | ARG | 33.413 | 40.08 | 43.378 |
| ATOM27 | N | ILE | 31.298 | 39.997 | 42.62 |
| ATOM28 | H | ILE | 30.599 | 40.482 | 42.075 |
| ATOM29 | CA | ILE | 30.958 | 38.785 | 43.399 |
| ATOM30 | HA | ILE | 31.733 | 38.694 | 44.16 |
| ATOM31 | CB | ILE | 29.621 | 38.96 | 44.069 |
| ATOM32 | HB | ILE | 29.376 | 38.179 | 44.789 |
| ATOM33 | CG2 | ILE | 29.647 | 40.244 | 44.889 |
| ATOM34 | HG21 | ILE | 30.604 | 40.212 | 45.409 |
| ATOM35 | HG22 | ILE | 29.492 | 41.134 | 44.278 |
| ATOM36 | HG23 | ILE | 28.817 | 40.196 | 45.593 |
| ATOM37 | CG1 | ILE | 28.425 | 38.988 | 43.086 |
| ATOM38 | HG12 | ILE | 28.287 | 39.968 | 42.629 |
| ATOM39 | HG13 | ILE | 28.619 | 38.367 | 42.212 |
| ATOM40 | CD1 | ILE | 27.016 | 38.561 | 43.691 |
| ATOM41 | HD11 | ILE | 27.101 | 37.583 | 44.163 |
| ATOM42 | HD12 | ILE | 26.633 | 39.279 | 44.416 |
| ATOM43 | HD13 | ILE | 26.34 | 38.473 | 42.84 |
| ATOM44 | C | ILE | 31.064 | 37.538 | 42.59 |
| ATOM45 | O | ILE | 30.949 | 37.586 | 41.374 |
| ATOM46 | N | TYR | 31.25 | 36.41 | 43.262 |
| ATOM47 | H | TYR | 31.393 | 36.523 | 44.255 |
| ATOM48 | CA | TYR | 31.375 | 35.068 | 42.733 |
| ATOM49 | HA | TYR | 31.622 | 35.112 | 41.672 |
| ATOM50 | CB | TYR | 32.36 | 34.232 | 43.611 |
| ATOM51 | HB2 | TYR | 33.335 | 34.631 | 43.331 |
| ATOM52 | HB3 | TYR | 32.064 | 34.554 | 44.61 |
| ATOM53 | CG | TYR | 32.41 | 32.708 | 43.509 |
| ATOM54 | CD1 | TYR | 31.559 | 31.918 | 44.321 |
| ATOM55 | HD1 | TYR | 30.791 | 32.399 | 44.909 |
| ATOM56 | CE1 | TYR | 31.581 | 30.492 | 44.269 |
| ATOM57 | HE1 | TYR | 30.855 | 29.885 | 44.79 |
| ATOM58 | CZ | TYR | 32.556 | 29.935 | 43.374 |
| ATOM59 | OH | TYR | 32.611 | 28.622 | 43.176 |
| ATOM60 | HH | TYR | 31.888 | 28.209 | 43.654 |
| ATOM61 | CE2 | TYR | 33.531 | 30.738 | 42.767 |
| ATOM62 | HE2 | TYR | 34.249 | 30.191 | 42.174 |
| ATOM63 | CD2 | TYR | 33.484 | 32.117 | 42.775 |
| ATOM64 | HD2 | TYR | 34.156 | 32.729 | 42.192 |
| ATOM65 | C | TYR | 29.988 | 34.446 | 42.835 |
| ATOM66 | O | TYR | 29.314 | 34.45 | 43.857 |
| ATOM67 | N | VAL | 29.552 | 33.783 | 41.784 |
| ATOM68 | H | VAL | 29.94 | 33.957 | 40.868 |
| ATOM69 | CA | VAL | 28.437 | 32.787 | 41.747 |
| ATOM70 | HA | VAL | 27.945 | 32.64 | 42.709 |
| ATOM71 | CB | VAL | 27.44 | 33.323 | 40.752 |
| ATOM72 | HB | VAL | 27.925 | 33.367 | 39.777 |
| ATOM73 | CG1 | VAL | 26.103 | 32.469 | 40.688 |
| ATOM74 | HG11 | VAL | 25.571 | 32.586 | 41.632 |
| ATOM75 | HG12 | VAL | 25.396 | 32.714 | 39.895 |
| ATOM76 | HG13 | VAL | 26.386 | 31.421 | 40.584 |
| ATOM77 | CG2 | VAL | 26.951 | 34.724 | 41 |
| ATOM78 | HG21 | VAL | 26.621 | 34.817 | 42.035 |
| ATOM79 | HG22 | VAL | 27.766 | 35.415 | 40.787 |
| ATOM80 | HG23 | VAL | 26.078 | 34.989 | 40.404 |
| ATOM81 | C | VAL | 28.944 | 31.419 | 41.245 |
| ATOM82 | O | VAL | 29.271 | 31.294 | 40.041 |
| ATOM83 | N | GLY | 29.003 | 30.414 | 42.128 |
| ATOM84 | H | GLY | 28.714 | 30.614 | 43.075 |
| ATOM85 | CA | GLY | 29.321 | 29.02 | 41.709 |
| ATOM86 | HA2 | GLY | 29.264 | 28.932 | 40.625 |
| ATOM87 | HA3 | GLY | 30.331 | 28.804 | 42.058 |
| ATOM88 | C | GLY | 28.379 | 27.955 | 42.144 |
| ATOM89 | O | GLY | 27.348 | 28.242 | 42.721 |
| ATOM90 | N | ASN | 28.837 | 26.686 | 41.873 |
| ATOM91 | H | ASN | 29.734 | 26.577 | 41.422 |
| ATOM92 | CA | ASN | 27.924 | 25.537 | 41.818 |
| ATOM93 | HA | ASN | 28.508 | 24.663 | 41.528 |
| ATOM94 | CB | ASN | 27.415 | 25.204 | 43.253 |
| ATOM95 | HB2 | ASN | 28.161 | 25.47 | 44.002 |
| ATOM96 | HB3 | ASN | 26.489 | 25.77 | 43.354 |
| ATOM97 | CG | ASN | 26.96 | 23.781 | 43.436 |
| ATOM98 | OD1 | ASN | 27.647 | 22.834 | 43.072 |
| ATOM99 | ND2 | ASN | 25.759 | 23.526 | 44.037 |
| ATOM100 | HD21 | ASN | 25.179 | 24.327 | 44.24 |
| ATOM101 | HD22 | ASN | 25.464 | 22.562 | 43.975 |
| ATOM102 | C | ASN | 26.903 | 25.702 | 40.729 |
| ATOM103 | O | ASN | 25.707 | 25.505 | 40.995 |
| ATOM104 | N | LEU | 27.32 | 26.093 | 39.558 |
| ATOM105 | H | LEU | 28.319 | 26.148 | 39.417 |
| ATOM106 | CA | LEU | 26.372 | 26.264 | 38.484 |
| ATOM107 | HA | LEU | 25.485 | 26.784 | 38.845 |
| ATOM108 | CB | LEU | 26.949 | 27.214 | 37.413 |
| ATOM109 | HB2 | LEU | 27.778 | 26.574 | 37.111 |
| ATOM110 | HB3 | LEU | 26.231 | 27.229 | 36.592 |
| ATOM111 | CG | LEU | 27.354 | 28.602 | 37.881 |
| ATOM112 | HG | LEU | 28.059 | 28.487 | 38.705 |
| ATOM113 | CD1 | LEU | 28.024 | 29.4 | 36.752 |
| ATOM114 | HD11 | LEU | 28.117 | 30.408 | 37.154 |
| ATOM115 | HD12 | LEU | 28.901 | 28.83 | 36.443 |
| ATOM116 | HD13 | LEU | 27.397 | 29.538 | 35.871 |
| ATOM117 | CD2 | LEU | 26.122 | 29.418 | 38.276 |
| ATOM118 | HD21 | LEU | 25.488 | 29.605 | 37.41 |
| ATOM119 | HD22 | LEU | 25.589 | 29.001 | 39.131 |
| ATOM120 | HD23 | LEU | 26.573 | 30.362 | 38.584 |
| ATOM121 | C | LEU | 25.812 | 24.919 | 37.89 |
| ATOM122 | O | LEU | 26.525 | 23.951 | 37.978 |
| ATOM123 | N | PRO | 24.696 | 24.965 | 37.086 |
| ATOM124 | CD | PRO | 23.708 | 26.056 | 36.871 |
| ATOM125 | HD2 | PRO | 24.048 | 26.791 | 36.141 |
| ATOM126 | HD3 | PRO | 23.502 | 26.563 | 37.813 |
| ATOM127 | CG | PRO | 22.432 | 25.412 | 36.327 |
| ATOM128 | HG2 | PRO | 21.94 | 26.143 | 35.685 |
| ATOM129 | HG3 | PRO | 21.98 | 25.059 | 37.254 |
| ATOM130 | CB | PRO | 22.992 | 24.249 | 35.576 |
| ATOM131 | HB2 | PRO | 23.021 | 24.613 | 34.549 |
| ATOM132 | HB3 | PRO | 22.278 | 23.431 | 35.675 |
| ATOM133 | CA | PRO | 24.348 | 23.855 | 36.158 |
| ATOM134 | HA | PRO | 24.201 | 22.979 | 36.79 |
| ATOM135 | C | PRO | 25.355 | 23.614 | 34.959 |
| ATOM136 | O | PRO | 25.884 | 24.598 | 34.447 |
| ATOM137 | N | PRO | 25.418 | 22.406 | 34.403 |
| ATOM138 | CD | PRO | 24.888 | 21.144 | 34.92 |
| ATOM139 | HD2 | PRO | 23.833 | 21.029 | 34.675 |
| ATOM140 | HD3 | PRO | 24.992 | 21.17 | 36.005 |
| ATOM141 | CG | PRO | 25.735 | 20.019 | 34.337 |
| ATOM142 | HG2 | PRO | 25.134 | 19.109 | 34.345 |
| ATOM143 | HG3 | PRO | 26.696 | 19.855 | 34.825 |
| ATOM144 | CB | PRO | 25.998 | 20.56 | 32.959 |
| ATOM145 | HB2 | PRO | 25.2 | 20.3 | 32.264 |
| ATOM146 | HB3 | PRO | 26.933 | 20.168 | 32.559 |
| ATOM147 | CA | PRO | 26.194 | 22.092 | 33.197 |
| ATOM148 | HA | PRO | 27.236 | 22.302 | 33.439 |
| ATOM149 | C | PRO | 25.879 | 22.816 | 31.935 |
| ATOM150 | O | PRO | 26.838 | 23.151 | 31.156 |
| ATOM151 | N | ASP | 24.619 | 23.17 | 31.574 |
| ATOM152 | H | ASP | 23.853 | 22.854 | 32.151 |
| ATOM153 | CA | ASP | 24.355 | 23.879 | 30.29 |
| ATOM154 | HA | ASP | 25.289 | 23.89 | 29.729 |
| ATOM155 | CB | ASP | 23.406 | 22.986 | 29.451 |
| ATOM156 | HB2 | ASP | 23.339 | 23.446 | 28.465 |
| ATOM157 | HB3 | ASP | 23.855 | 22.008 | 29.28 |
| ATOM158 | CG | ASP | 21.974 | 22.834 | 29.966 |
| ATOM159 | OD1 | ASP | 21.762 | 23.346 | 31.12 |
| ATOM160 | OD2 | ASP | 21.036 | 22.252 | 29.328 |
| ATOM161 | C | ASP | 23.948 | 25.329 | 30.395 |
| ATOM162 | O | ASP | 23.325 | 25.898 | 29.481 |
| ATOM163 | N | ILE | 24.298 | 25.958 | 31.515 |
| ATOM164 | H | ILE | 24.773 | 25.424 | 32.229 |
| ATOM165 | CA | ILE | 24.134 | 27.418 | 31.711 |
| ATOM166 | HA | ILE | 23.064 | 27.609 | 31.621 |
| ATOM167 | CB | ILE | 24.544 | 27.861 | 33.167 |
| ATOM168 | HB | ILE | 24.001 | 27.217 | 33.857 |
| ATOM169 | CG2 | ILE | 26.046 | 27.777 | 33.38 |
| ATOM170 | HG21 | ILE | 26.14 | 27.973 | 34.448 |
| ATOM171 | HG22 | ILE | 26.403 | 26.753 | 33.269 |
| ATOM172 | HG23 | ILE | 26.576 | 28.553 | 32.828 |
| ATOM173 | CG1 | ILE | 23.966 | 29.294 | 33.266 |
| ATOM174 | HG12 | ILE | 24.482 | 30.053 | 32.678 |
| ATOM175 | HG13 | ILE | 22.891 | 29.36 | 33.099 |
| ATOM176 | CD1 | ILE | 24.049 | 29.811 | 34.695 |
| ATOM177 | HD11 | ILE | 23.231 | 30.488 | 34.941 |
| ATOM178 | HD12 | ILE | 23.901 | 28.979 | 35.384 |
| ATOM179 | HD13 | ILE | 25.019 | 30.273 | 34.879 |
| ATOM180 | C | ILE | 24.82 | 28.233 | 30.651 |
| ATOM181 | O | ILE | 25.999 | 28.052 | 30.35 |
| ATOM182 | N | ARG | 24.015 | 29.218 | 30.194 |
| ATOM183 | H | ARG | 23.055 | 29.324 | 30.489 |
| ATOM184 | CA | ARG | 24.537 | 30.165 | 29.224 |
| ATOM185 | HA | ARG | 25.584 | 29.896 | 29.084 |
| ATOM186 | CB | ARG | 23.761 | 29.952 | 27.956 |
| ATOM187 | HB2 | ARG | 22.691 | 30.147 | 28.03 |
| ATOM188 | HB3 | ARG | 24.211 | 30.643 | 27.244 |
| ATOM189 | CG | ARG | 23.897 | 28.611 | 27.303 |
| ATOM190 | HG2 | ARG | 24.931 | 28.345 | 27.525 |
| ATOM191 | HG3 | ARG | 23.318 | 27.762 | 27.667 |
| ATOM192 | CD | ARG | 23.508 | 28.642 | 25.814 |
| ATOM193 | HD2 | ARG | 23.191 | 27.635 | 25.543 |
| ATOM194 | HD3 | ARG | 22.569 | 29.148 | 25.588 |
| ATOM195 | NE | ARG | 24.554 | 29.114 | 25.018 |
| ATOM196 | HE | ARG | 25.361 | 29.596 | 25.387 |
| ATOM197 | CZ | ARG | 24.553 | 29.019 | 23.729 |
| ATOM198 | NH1 | ARG | 23.459 | 28.608 | 23.051 |
| ATOM199 | HH11 | ARG | 22.606 | 28.239 | 23.447 |
| ATOM200 | HH12 | ARG | 23.653 | 28.524 | 22.064 |
| ATOM201 | NH2 | ARG | 25.581 | 29.432 | 23.032 |
| ATOM202 | HH21 | ARG | 26.452 | 29.642 | 23.498 |
| ATOM203 | HH22 | ARG | 25.527 | 29.514 | 22.026 |
| ATOM204 | C | ARG | 24.518 | 31.563 | 29.751 |
| ATOM205 | O | ARG | 23.851 | 31.801 | 30.736 |
| ATOM206 | N | THR | 25.335 | 32.45 | 29.234 |
| ATOM207 | H | THR | 25.933 | 32.129 | 28.486 |
| ATOM208 | CA | THR | 25.752 | 33.767 | 29.77 |
| ATOM209 | HA | THR | 26.358 | 33.643 | 30.667 |
| ATOM210 | CB | THR | 26.673 | 34.483 | 28.677 |
| ATOM211 | HB | THR | 26.396 | 34.201 | 27.662 |
| ATOM212 | CG2 | THR | 26.872 | 36 | 28.834 |
| ATOM213 | HG21 | THR | 27.09 | 36.287 | 29.863 |
| ATOM214 | HG22 | THR | 27.671 | 36.388 | 28.203 |
| ATOM215 | HG23 | THR | 25.945 | 36.5 | 28.554 |
| ATOM216 | OG1 | THR | 27.964 | 33.889 | 28.825 |
| ATOM217 | HG1 | THR | 27.873 | 32.986 | 28.514 |
| ATOM218 | C | THR | 24.559 | 34.62 | 30.353 |
| ATOM219 | O | THR | 24.633 | 35.022 | 31.513 |
| ATOM220 | N | LYS | 23.541 | 34.971 | 29.53 |
| ATOM221 | H | LYS | 23.583 | 34.808 | 28.534 |
| ATOM222 | CA | LYS | 22.32 | 35.752 | 29.938 |
| ATOM223 | HA | LYS | 22.657 | 36.665 | 30.428 |
| ATOM224 | CB | LYS | 21.657 | 36.244 | 28.667 |
| ATOM225 | HB2 | LYS | 21.579 | 35.395 | 27.987 |
| ATOM226 | HB3 | LYS | 20.643 | 36.517 | 28.959 |
| ATOM227 | CG | LYS | 22.353 | 37.347 | 27.932 |
| ATOM228 | HG2 | LYS | 22.29 | 38.312 | 28.435 |
| ATOM229 | HG3 | LYS | 23.404 | 37.059 | 27.91 |
| ATOM230 | CD | LYS | 21.831 | 37.496 | 26.511 |
| ATOM231 | HD2 | LYS | 22.485 | 36.812 | 25.969 |
| ATOM232 | HD3 | LYS | 20.856 | 37.033 | 26.361 |
| ATOM233 | CE | LYS | 21.739 | 38.903 | 25.929 |
| ATOM234 | HE2 | LYS | 21.387 | 38.966 | 24.899 |
| ATOM235 | HE3 | LYS | 20.947 | 39.446 | 26.444 |
| ATOM236 | NZ | LYS | 23.039 | 39.572 | 26.061 |
| ATOM237 | HZ1 | LYS | 23.645 | 39.295 | 25.302 |
| ATOM238 | HZ2 | LYS | 22.867 | 40.561 | 25.951 |
| ATOM239 | HZ3 | LYS | 23.501 | 39.351 | 26.932 |
| ATOM240 | C | LYS | 21.372 | 35.101 | 30.949 |
| ATOM241 | O | LYS | 20.548 | 35.784 | 31.574 |
| ATOM242 | N | ASP | 21.536 | 33.787 | 31.207 |
| ATOM243 | H | ASP | 22.266 | 33.311 | 30.695 |
| ATOM244 | CA | ASP | 20.795 | 33.116 | 32.226 |
| ATOM245 | HA | ASP | 19.749 | 33.398 | 32.105 |
| ATOM246 | CB | ASP | 20.988 | 31.606 | 32.189 |
| ATOM247 | HB2 | ASP | 21.943 | 31.503 | 32.705 |
| ATOM248 | HB3 | ASP | 20.221 | 31.182 | 32.838 |
| ATOM249 | CG | ASP | 20.997 | 30.745 | 30.913 |
| ATOM250 | OD1 | ASP | 21.423 | 29.563 | 31.063 |
| ATOM251 | OD2 | ASP | 20.582 | 31.22 | 29.842 |
| ATOM252 | C | ASP | 21.231 | 33.583 | 33.585 |
| ATOM253 | O | ASP | 20.371 | 33.813 | 34.409 |
| ATOM254 | N | ILE | 22.49 | 33.978 | 33.895 |
| ATOM255 | H | ILE | 23.152 | 34.032 | 33.134 |
| ATOM256 | CA | ILE | 22.954 | 34.576 | 35.137 |
| ATOM257 | HA | ILE | 22.555 | 33.947 | 35.932 |
| ATOM258 | CB | ILE | 24.476 | 34.717 | 35.248 |
| ATOM259 | HB | ILE | 24.909 | 35.477 | 34.598 |
| ATOM260 | CG2 | ILE | 24.819 | 35.232 | 36.645 |
| ATOM261 | HG21 | ILE | 25.904 | 35.301 | 36.729 |
| ATOM262 | HG22 | ILE | 24.487 | 36.259 | 36.795 |
| ATOM263 | HG23 | ILE | 24.436 | 34.631 | 37.47 |
| ATOM264 | CG1 | ILE | 25.156 | 33.331 | 35.046 |
| ATOM265 | HG12 | ILE | 25.017 | 32.741 | 35.952 |
| ATOM266 | HG13 | ILE | 24.665 | 32.887 | 34.181 |
| ATOM267 | CD1 | ILE | 26.616 | 33.31 | 34.717 |
| ATOM268 | HD11 | ILE | 27.148 | 33.896 | 35.466 |
| ATOM269 | HD12 | ILE | 27.029 | 32.303 | 34.781 |
| ATOM270 | HD13 | ILE | 26.825 | 33.773 | 33.753 |
| ATOM271 | C | ILE | 22.31 | 35.962 | 35.305 |
| ATOM272 | O | ILE | 21.92 | 36.419 | 36.37 |
| ATOM273 | N | GLU | 22.2 | 36.766 | 34.196 |
| ATOM274 | H | GLU | 22.662 | 36.456 | 33.354 |
| ATOM275 | CA | GLU | 21.566 | 38.139 | 34.335 |
| ATOM276 | HA | GLU | 22.171 | 38.654 | 35.081 |
| ATOM277 | CB | GLU | 21.694 | 38.951 | 32.968 |
| ATOM278 | HB2 | GLU | 21.478 | 38.318 | 32.107 |
| ATOM279 | HB3 | GLU | 20.904 | 39.703 | 32.956 |
| ATOM280 | CG | GLU | 23.036 | 39.62 | 32.761 |
| ATOM281 | HG2 | GLU | 23.321 | 40.268 | 33.59 |
| ATOM282 | HG3 | GLU | 23.735 | 38.791 | 32.652 |
| ATOM283 | CD | GLU | 23.268 | 40.394 | 31.455 |
| ATOM284 | OE1 | GLU | 22.287 | 40.907 | 30.883 |
| ATOM285 | OE2 | GLU | 24.383 | 40.613 | 30.965 |
| ATOM286 | C | GLU | 20.076 | 38.006 | 34.661 |
| ATOM287 | O | GLU | 19.548 | 38.744 | 35.486 |
| ATOM288 | N | ASP | 19.387 | 37.024 | 34.079 |
| ATOM289 | H | ASP | 20.02 | 36.419 | 33.574 |
| ATOM290 | CA | ASP | 17.961 | 36.711 | 34.464 |
| ATOM291 | HA | ASP | 17.436 | 37.666 | 34.499 |
| ATOM292 | CB | ASP | 17.25 | 36.045 | 33.339 |
| ATOM293 | HB2 | ASP | 17.823 | 35.121 | 33.267 |
| ATOM294 | HB3 | ASP | 16.29 | 35.769 | 33.776 |
| ATOM295 | CG | ASP | 17.161 | 36.89 | 32.069 |
| ATOM296 | OD1 | ASP | 16.975 | 38.092 | 32.22 |
| ATOM297 | OD2 | ASP | 16.887 | 36.263 | 31.027 |
| ATOM298 | C | ASP | 17.676 | 35.988 | 35.826 |
| ATOM299 | O | ASP | 16.518 | 35.856 | 36.332 |
| ATOM300 | N | VAL | 18.77 | 35.636 | 36.527 |
| ATOM301 | H | VAL | 19.66 | 35.715 | 36.056 |
| ATOM302 | CA | VAL | 18.783 | 35.279 | 37.945 |
| ATOM303 | HA | VAL | 17.766 | 34.999 | 38.222 |
| ATOM304 | CB | VAL | 19.783 | 34.031 | 38.23 |
| ATOM305 | HB | VAL | 20.774 | 34.39 | 37.953 |
| ATOM306 | CG1 | VAL | 19.933 | 33.74 | 39.7 |
| ATOM307 | HG11 | VAL | 18.996 | 33.485 | 40.195 |
| ATOM308 | HG12 | VAL | 20.508 | 32.817 | 39.774 |
| ATOM309 | HG13 | VAL | 20.618 | 34.509 | 40.055 |
| ATOM310 | CG2 | VAL | 19.382 | 32.727 | 37.48 |
| ATOM311 | HG21 | VAL | 18.355 | 32.46 | 37.73 |
| ATOM312 | HG22 | VAL | 19.499 | 32.794 | 36.399 |
| ATOM313 | HG23 | VAL | 20.09 | 32.003 | 37.883 |
| ATOM314 | C | VAL | 19.155 | 36.536 | 38.814 |
| ATOM315 | O | VAL | 18.919 | 36.536 | 40.027 |
| ATOM316 | N | PHE | 19.794 | 37.608 | 38.33 |
| ATOM317 | H | PHE | 20.103 | 37.647 | 37.37 |
| ATOM318 | CA | PHE | 20.34 | 38.667 | 39.207 |
| ATOM319 | HA | PHE | 20.076 | 38.428 | 40.237 |
| ATOM320 | CB | PHE | 21.866 | 38.608 | 39.166 |
| ATOM321 | HB2 | PHE | 22.15 | 38.462 | 38.124 |
| ATOM322 | HB3 | PHE | 22.255 | 39.618 | 39.296 |
| ATOM323 | CG | PHE | 22.464 | 37.528 | 40.105 |
| ATOM324 | CD1 | PHE | 22.872 | 36.286 | 39.649 |
| ATOM325 | HD1 | PHE | 22.752 | 36.068 | 38.599 |
| ATOM326 | CE1 | PHE | 23.658 | 35.384 | 40.423 |
| ATOM327 | HE1 | PHE | 23.976 | 34.544 | 39.823 |
| ATOM328 | CZ | PHE | 23.849 | 35.629 | 41.823 |
| ATOM329 | HZ | PHE | 24.348 | 34.883 | 42.424 |
| ATOM330 | CE2 | PHE | 23.398 | 36.861 | 42.311 |
| ATOM331 | HE2 | PHE | 23.455 | 37.094 | 43.364 |
| ATOM332 | CD2 | PHE | 22.779 | 37.852 | 41.448 |
| ATOM333 | HD2 | PHE | 22.45 | 38.815 | 41.808 |
| ATOM334 | C | PHE | 19.824 | 40.077 | 39.02 |
| ATOM335 | O | PHE | 20.113 | 40.919 | 39.807 |
| ATOM336 | N | TYR | 18.953 | 40.273 | 38.034 |
| ATOM337 | H | TYR | 18.843 | 39.581 | 37.307 |
| ATOM338 | CA | TYR | 18.458 | 41.594 | 37.744 |
| ATOM339 | HA | TYR | 19.366 | 42.187 | 37.636 |
| ATOM340 | CB | TYR | 17.573 | 41.453 | 36.495 |
| ATOM341 | HB2 | TYR | 17.621 | 42.436 | 36.027 |
| ATOM342 | HB3 | TYR | 17.989 | 40.864 | 35.678 |
| ATOM343 | CG | TYR | 16.104 | 40.96 | 36.668 |
| ATOM344 | CD1 | TYR | 15.777 | 39.581 | 36.563 |
| ATOM345 | HD1 | TYR | 16.596 | 38.901 | 36.383 |
| ATOM346 | CE1 | TYR | 14.488 | 39.119 | 36.649 |
| ATOM347 | HE1 | TYR | 14.285 | 38.059 | 36.597 |
| ATOM348 | CZ | TYR | 13.492 | 40.08 | 37.043 |
| ATOM349 | OH | TYR | 12.19 | 39.644 | 37.3 |
| ATOM350 | HH | TYR | 11.646 | 40.343 | 37.67 |
| ATOM351 | CE2 | TYR | 13.815 | 41.431 | 37.096 |
| ATOM352 | HE2 | TYR | 13.001 | 42.136 | 37.176 |
| ATOM353 | CD2 | TYR | 15.088 | 41.894 | 36.853 |
| ATOM354 | HD2 | TYR | 15.397 | 42.929 | 36.82 |
| ATOM355 | C | TYR | 17.654 | 42.292 | 38.813 |
| ATOM356 | O | TYR | 17.698 | 43.562 | 38.816 |
| ATOM357 | N | LYS | 16.908 | 41.618 | 39.673 |
| ATOM358 | H | LYS | 16.852 | 40.62 | 39.529 |
| ATOM359 | CA | LYS | 16.074 | 42.23 | 40.712 |
| ATOM360 | HA | LYS | 15.294 | 42.88 | 40.314 |
| ATOM361 | CB | LYS | 15.264 | 41.014 | 41.348 |
| ATOM362 | HB2 | LYS | 15.927 | 40.259 | 41.769 |
| ATOM363 | HB3 | LYS | 14.708 | 41.356 | 42.221 |
| ATOM364 | CG | LYS | 14.3 | 40.393 | 40.338 |
| ATOM365 | HG2 | LYS | 13.622 | 41.199 | 40.06 |
| ATOM366 | HG3 | LYS | 14.748 | 40.007 | 39.423 |
| ATOM367 | CD | LYS | 13.532 | 39.237 | 41.031 |
| ATOM368 | HD2 | LYS | 14.151 | 38.34 | 41.054 |
| ATOM369 | HD3 | LYS | 13.336 | 39.592 | 42.042 |
| ATOM370 | CE | LYS | 12.268 | 38.801 | 40.271 |
| ATOM371 | HE2 | LYS | 11.697 | 39.685 | 39.988 |
| ATOM372 | HE3 | LYS | 12.589 | 38.465 | 39.285 |
| ATOM373 | NZ | LYS | 11.517 | 37.733 | 40.968 |
| ATOM374 | HZ1 | LYS | 11.104 | 38.118 | 41.806 |
| ATOM375 | HZ2 | LYS | 10.727 | 37.359 | 40.462 |
| ATOM376 | HZ3 | LYS | 12.172 | 36.996 | 41.188 |
| ATOM377 | C | LYS | 16.798 | 42.951 | 41.811 |
| ATOM378 | O | LYS | 16.195 | 43.771 | 42.527 |
| ATOM379 | N | TYR | 18.075 | 42.735 | 41.943 |
| ATOM380 | H | TYR | 18.498 | 42.139 | 41.246 |
| ATOM381 | CA | TYR | 18.909 | 43.366 | 42.912 |
| ATOM382 | HA | TYR | 18.258 | 43.68 | 43.728 |
| ATOM383 | CB | TYR | 19.88 | 42.263 | 43.552 |
| ATOM384 | HB2 | TYR | 20.404 | 41.721 | 42.765 |
| ATOM385 | HB3 | TYR | 20.463 | 42.75 | 44.333 |
| ATOM386 | CG | TYR | 19.174 | 41.111 | 44.178 |
| ATOM387 | CD1 | TYR | 18.92 | 39.933 | 43.435 |
| ATOM388 | HD1 | TYR | 19.298 | 39.743 | 42.441 |
| ATOM389 | CE1 | TYR | 18.364 | 38.787 | 44.028 |
| ATOM390 | HE1 | TYR | 18.143 | 37.886 | 43.475 |
| ATOM391 | CZ | TYR | 17.879 | 38.906 | 45.336 |
| ATOM392 | OH | TYR | 17.183 | 37.803 | 45.783 |
| ATOM393 | HH | TYR | 16.978 | 37.845 | 46.72 |
| ATOM394 | CE2 | TYR | 18.051 | 40.054 | 46.124 |
| ATOM395 | HE2 | TYR | 17.651 | 40.089 | 47.126 |
| ATOM396 | CD2 | TYR | 18.632 | 41.228 | 45.517 |
| ATOM397 | HD2 | TYR | 18.774 | 42.141 | 46.077 |
| ATOM398 | C | TYR | 19.638 | 44.605 | 42.471 |
| ATOM399 | O | TYR | 20.016 | 45.331 | 43.355 |
| ATOM400 | N | GLY | 19.884 | 44.742 | 41.141 |
| ATOM401 | H | GLY | 19.553 | 44.068 | 40.466 |
| ATOM402 | CA | GLY | 20.621 | 45.911 | 40.524 |
| ATOM403 | HA2 | GLY | 20.096 | 46.852 | 40.685 |
| ATOM404 | HA3 | GLY | 21.59 | 45.898 | 41.024 |
| ATOM405 | C | GLY | 20.789 | 45.953 | 38.975 |
| ATOM406 | O | GLY | 20.475 | 44.886 | 38.44 |
| ATOM407 | N | ALA | 21.52 | 46.909 | 38.498 |
| ATOM408 | H | ALA | 21.862 | 47.629 | 39.118 |
| ATOM409 | CA | ALA | 22.107 | 46.886 | 37.188 |
| ATOM410 | HA | ALA | 21.379 | 46.581 | 36.436 |
| ATOM411 | CB | ALA | 22.258 | 48.317 | 36.798 |
| ATOM412 | HB1 | ALA | 22.938 | 48.776 | 37.515 |
| ATOM413 | HB2 | ALA | 22.828 | 48.45 | 35.879 |
| ATOM414 | HB3 | ALA | 21.307 | 48.843 | 36.705 |
| ATOM415 | C | ALA | 23.417 | 46.107 | 37.163 |
| ATOM416 | O | ALA | 24.425 | 46.231 | 37.892 |
| ATOM417 | N | ILE | 23.498 | 45.378 | 36.051 |
| ATOM418 | H | ILE | 22.844 | 45.506 | 35.291 |
| ATOM419 | CA | ILE | 24.71 | 44.504 | 35.759 |
| ATOM420 | HA | ILE | 25.182 | 44.293 | 36.719 |
| ATOM421 | CB | ILE | 24.265 | 43.115 | 35.201 |
| ATOM422 | HB | ILE | 23.656 | 43.333 | 34.324 |
| ATOM423 | CG2 | ILE | 25.536 | 42.482 | 34.595 |
| ATOM424 | HG21 | ILE | 25.292 | 41.486 | 34.226 |
| ATOM425 | HG22 | ILE | 25.922 | 42.915 | 33.672 |
| ATOM426 | HG23 | ILE | 26.333 | 42.401 | 35.335 |
| ATOM427 | CG1 | ILE | 23.555 | 42.153 | 36.166 |
| ATOM428 | HG12 | ILE | 23.98 | 41.164 | 35.996 |
| ATOM429 | HG13 | ILE | 23.985 | 42.402 | 37.137 |
| ATOM430 | CD1 | ILE | 22.034 | 42.027 | 36.226 |
| ATOM431 | HD11 | ILE | 21.655 | 43.038 | 36.376 |
| ATOM432 | HD12 | ILE | 21.678 | 41.669 | 35.26 |
| ATOM433 | HD13 | ILE | 21.686 | 41.328 | 36.986 |
| ATOM434 | C | ILE | 25.73 | 45.268 | 34.854 |
| ATOM435 | O | ILE | 25.414 | 45.577 | 33.713 |
| ATOM436 | N | ARG | 26.964 | 45.433 | 35.359 |
| ATOM437 | H | ARG | 27.121 | 45.24 | 36.338 |
| ATOM438 | CA | ARG | 28.044 | 46.028 | 34.516 |
| ATOM439 | HA | ARG | 27.734 | 46.821 | 33.837 |
| ATOM440 | CB | ARG | 29.051 | 46.727 | 35.492 |
| ATOM441 | HB2 | ARG | 28.494 | 47.353 | 36.189 |
| ATOM442 | HB3 | ARG | 29.592 | 45.992 | 36.088 |
| ATOM443 | CG | ARG | 30.104 | 47.466 | 34.749 |
| ATOM444 | HG2 | ARG | 30.612 | 46.723 | 34.134 |
| ATOM445 | HG3 | ARG | 29.621 | 48.126 | 34.028 |
| ATOM446 | CD | ARG | 31.093 | 48.15 | 35.658 |
| ATOM447 | HD2 | ARG | 31.495 | 47.402 | 36.342 |
| ATOM448 | HD3 | ARG | 31.88 | 48.511 | 34.996 |
| ATOM449 | NE | ARG | 30.459 | 49.267 | 36.408 |
| ATOM450 | HE | ARG | 29.515 | 49.483 | 36.123 |
| ATOM451 | CZ | ARG | 30.968 | 49.996 | 37.337 |
| ATOM452 | NH1 | ARG | 32.176 | 49.941 | 37.753 |
| ATOM453 | HH11 | ARG | 32.952 | 49.531 | 37.254 |
| ATOM454 | HH12 | ARG | 32.413 | 50.646 | 38.436 |
| ATOM455 | NH2 | ARG | 30.293 | 50.953 | 37.936 |
| ATOM456 | HH21 | ARG | 29.353 | 51.107 | 37.602 |
| ATOM457 | HH22 | ARG | 30.735 | 51.593 | 38.58 |
| ATOM458 | C | ARG | 28.725 | 44.978 | 33.671 |
| ATOM459 | O | ARG | 28.968 | 45.243 | 32.474 |
| ATOM460 | N | ASP | 28.888 | 43.757 | 34.25 |
| ATOM461 | H | ASP | 28.754 | 43.677 | 35.248 |
| ATOM462 | CA | ASP | 29.542 | 42.593 | 33.606 |
| ATOM463 | HA | ASP | 29.341 | 42.531 | 32.537 |
| ATOM464 | CB | ASP | 31.137 | 42.795 | 33.562 |
| ATOM465 | HB2 | ASP | 31.489 | 43.116 | 34.542 |
| ATOM466 | HB3 | ASP | 31.593 | 41.865 | 33.221 |
| ATOM467 | CG | ASP | 31.524 | 43.758 | 32.359 |
| ATOM468 | OD1 | ASP | 32.417 | 44.663 | 32.571 |
| ATOM469 | OD2 | ASP | 31.041 | 43.588 | 31.221 |
| ATOM470 | C | ASP | 29.301 | 41.234 | 34.157 |
| ATOM471 | O | ASP | 29.215 | 41.118 | 35.398 |
| ATOM472 | N | ILE | 29.034 | 40.199 | 33.347 |
| ATOM473 | H | ILE | 28.83 | 40.471 | 32.396 |
| ATOM474 | CA | ILE | 29.123 | 38.746 | 33.674 |
| ATOM475 | HA | ILE | 29.066 | 38.568 | 34.748 |
| ATOM476 | CB | ILE | 27.909 | 37.976 | 33.108 |
| ATOM477 | HB | ILE | 28.039 | 38.075 | 32.031 |
| ATOM478 | CG2 | ILE | 27.856 | 36.495 | 33.486 |
| ATOM479 | HG21 | ILE | 28.049 | 36.308 | 34.542 |
| ATOM480 | HG22 | ILE | 26.975 | 35.96 | 33.13 |
| ATOM481 | HG23 | ILE | 28.699 | 36.047 | 32.96 |
| ATOM482 | CG1 | ILE | 26.573 | 38.583 | 33.549 |
| ATOM483 | HG12 | ILE | 26.507 | 39.591 | 33.139 |
| ATOM484 | HG13 | ILE | 25.83 | 38.014 | 32.99 |
| ATOM485 | CD1 | ILE | 26.216 | 38.653 | 35.017 |
| ATOM486 | HD11 | ILE | 25.408 | 39.36 | 35.201 |
| ATOM487 | HD12 | ILE | 25.928 | 37.73 | 35.521 |
| ATOM488 | HD13 | ILE | 27.131 | 39.038 | 35.468 |
| ATOM489 | C | ILE | 30.5 | 38.214 | 33.113 |
| ATOM490 | O | ILE | 31.039 | 38.757 | 32.152 |
| ATOM491 | N | ASP | 30.974 | 37.081 | 33.649 |
| ATOM492 | H | ASP | 30.343 | 36.712 | 34.346 |
| ATOM493 | CA | ASP | 32.178 | 36.406 | 33.192 |
| ATOM494 | HA | ASP | 32.35 | 36.346 | 32.117 |
| ATOM495 | CB | ASP | 33.418 | 37.131 | 33.772 |
| ATOM496 | HB2 | ASP | 33.491 | 38.159 | 33.417 |
| ATOM497 | HB3 | ASP | 33.516 | 37.049 | 34.855 |
| ATOM498 | CG | ASP | 34.804 | 36.649 | 33.309 |
| ATOM499 | OD1 | ASP | 34.84 | 35.86 | 32.328 |
| ATOM500 | OD2 | ASP | 35.856 | 37.12 | 33.795 |
| ATOM501 | C | ASP | 32.097 | 34.923 | 33.666 |
| ATOM502 | O | ASP | 32.501 | 34.74 | 34.807 |
| ATOM503 | N | LEU | 31.534 | 33.999 | 32.892 |
| ATOM504 | H | LEU | 31.401 | 34.218 | 31.915 |
| ATOM505 | CA | LEU | 31.497 | 32.561 | 33.156 |
| ATOM506 | HA | LEU | 31.219 | 32.291 | 34.175 |
| ATOM507 | CB | LEU | 30.241 | 32.013 | 32.463 |
| ATOM508 | HB2 | LEU | 29.42 | 32.533 | 32.956 |
| ATOM509 | HB3 | LEU | 30.274 | 32.414 | 31.451 |
| ATOM510 | CG | LEU | 29.924 | 30.501 | 32.367 |
| ATOM511 | HG | LEU | 30.799 | 29.912 | 32.092 |
| ATOM512 | CD1 | LEU | 29.376 | 29.891 | 33.607 |
| ATOM513 | HD11 | LEU | 28.511 | 30.421 | 34.005 |
| ATOM514 | HD12 | LEU | 29.273 | 28.839 | 33.34 |
| ATOM515 | HD13 | LEU | 30.141 | 29.926 | 34.383 |
| ATOM516 | CD2 | LEU | 28.795 | 30.319 | 31.319 |
| ATOM517 | HD21 | LEU | 27.9 | 30.869 | 31.611 |
| ATOM518 | HD22 | LEU | 29.099 | 30.516 | 30.291 |
| ATOM519 | HD23 | LEU | 28.528 | 29.266 | 31.231 |
| ATOM520 | C | LEU | 32.768 | 31.724 | 32.743 |
| ATOM521 | O | LEU | 33.285 | 31.967 | 31.643 |
| ATOM522 | N | LYS | 33.276 | 30.863 | 33.637 |
| ATOM523 | H | LYS | 32.64 | 30.656 | 34.394 |
| ATOM524 | CA | LYS | 34.477 | 30.041 | 33.505 |
| ATOM525 | HA | LYS | 35.052 | 30.406 | 32.654 |
| ATOM526 | CB | LYS | 35.41 | 30.219 | 34.704 |
| ATOM527 | HB2 | LYS | 34.953 | 29.629 | 35.498 |
| ATOM528 | HB3 | LYS | 36.324 | 29.673 | 34.471 |
| ATOM529 | CG | LYS | 35.639 | 31.64 | 35.257 |
| ATOM530 | HG2 | LYS | 34.647 | 31.942 | 35.593 |
| ATOM531 | HG3 | LYS | 36.424 | 31.677 | 36.013 |
| ATOM532 | CD | LYS | 36.14 | 32.605 | 34.087 |
| ATOM533 | HD2 | LYS | 36.56 | 32.11 | 33.212 |
| ATOM534 | HD3 | LYS | 35.228 | 33.116 | 33.78 |
| ATOM535 | CE | LYS | 37.166 | 33.605 | 34.647 |
| ATOM536 | HE2 | LYS | 36.749 | 33.937 | 35.598 |
| ATOM537 | HE3 | LYS | 38.059 | 33.025 | 34.882 |
| ATOM538 | NZ | LYS | 37.481 | 34.735 | 33.729 |
| ATOM539 | HZ1 | LYS | 37.966 | 34.351 | 32.93 |
| ATOM540 | HZ2 | LYS | 36.624 | 35.115 | 33.353 |
| ATOM541 | HZ3 | LYS | 37.941 | 35.552 | 34.106 |
| ATOM542 | C | LYS | 34.082 | 28.553 | 33.342 |
| ATOM543 | O | LYS | 33.276 | 28.04 | 34.131 |
| ATOM544 | N | ASN | 34.657 | 27.84 | 32.315 |
| ATOM545 | H | ASN | 35.144 | 28.348 | 31.591 |
| ATOM546 | CA | ASN | 34.699 | 26.42 | 32.175 |
| ATOM547 | HA | ASN | 33.71 | 25.962 | 32.166 |
| ATOM548 | CB | ASN | 35.315 | 25.981 | 30.772 |
| ATOM549 | HB2 | ASN | 34.639 | 26.3 | 29.978 |
| ATOM550 | HB3 | ASN | 36.282 | 26.465 | 30.637 |
| ATOM551 | CG | ASN | 35.293 | 24.468 | 30.576 |
| ATOM552 | OD1 | ASN | 36.26 | 23.752 | 30.664 |
| ATOM553 | ND2 | ASN | 34.156 | 23.882 | 30.337 |
| ATOM554 | HD21 | ASN | 33.302 | 24.331 | 30.037 |
| ATOM555 | HD22 | ASN | 34.326 | 22.892 | 30.227 |
| ATOM556 | C | ASN | 35.511 | 25.731 | 33.333 |
| ATOM557 | O | ASN | 36.506 | 26.255 | 33.826 |
| ATOM558 | N | ARG | 35.086 | 24.452 | 33.644 |
| ATOM559 | H | ARG | 34.238 | 24.207 | 33.154 |
| ATOM560 | CA | ARG | 35.799 | 23.43 | 34.417 |
| ATOM561 | HA | ARG | 36.855 | 23.696 | 34.392 |
| ATOM562 | CB | ARG | 35.29 | 23.371 | 35.827 |
| ATOM563 | HB2 | ARG | 34.237 | 23.089 | 35.826 |
| ATOM564 | HB3 | ARG | 35.869 | 22.553 | 36.256 |
| ATOM565 | CG | ARG | 35.574 | 24.673 | 36.61 |
| ATOM566 | HG2 | ARG | 36.636 | 24.912 | 36.552 |
| ATOM567 | HG3 | ARG | 35.018 | 25.541 | 36.253 |
| ATOM568 | CD | ARG | 35.169 | 24.452 | 38.07 |
| ATOM569 | HD2 | ARG | 35.389 | 25.342 | 38.66 |
| ATOM570 | HD3 | ARG | 34.095 | 24.278 | 38.136 |
| ATOM571 | NE | ARG | 35.832 | 23.39 | 38.848 |
| ATOM572 | HE | ARG | 36.832 | 23.532 | 38.864 |
| ATOM573 | CZ | ARG | 35.304 | 22.405 | 39.539 |
| ATOM574 | NH1 | ARG | 34.003 | 22.138 | 39.489 |
| ATOM575 | HH11 | ARG | 33.311 | 22.738 | 39.063 |
| ATOM576 | HH12 | ARG | 33.714 | 21.383 | 40.094 |
| ATOM577 | NH2 | ARG | 36.037 | 21.622 | 40.323 |
| ATOM578 | HH21 | ARG | 36.999 | 21.563 | 40.021 |
| ATOM579 | HH22 | ARG | 35.588 | 20.878 | 40.838 |
| ATOM580 | C | ARG | 35.598 | 22.097 | 33.76 |
| ATOM581 | O | ARG | 34.475 | 21.714 | 33.353 |
| ATOM582 | N | ARG | 36.644 | 21.299 | 33.8 |
| ATOM583 | H | ARG | 37.497 | 21.626 | 34.231 |
| ATOM584 | CA | ARG | 36.521 | 19.89 | 33.373 |
| ATOM585 | HA | ARG | 35.761 | 19.829 | 32.594 |
| ATOM586 | CB | ARG | 37.896 | 19.44 | 32.786 |
| ATOM587 | HB2 | ARG | 38.625 | 20.094 | 33.265 |
| ATOM588 | HB3 | ARG | 38.029 | 18.376 | 32.981 |
| ATOM589 | CG | ARG | 37.851 | 19.688 | 31.274 |
| ATOM590 | HG2 | ARG | 37.217 | 18.927 | 30.82 |
| ATOM591 | HG3 | ARG | 37.467 | 20.656 | 30.952 |
| ATOM592 | CD | ARG | 39.246 | 19.65 | 30.718 |
| ATOM593 | HD2 | ARG | 39.185 | 19.961 | 29.675 |
| ATOM594 | HD3 | ARG | 39.821 | 20.343 | 31.332 |
| ATOM595 | NE | ARG | 39.856 | 18.298 | 30.691 |
| ATOM596 | HE | ARG | 39.218 | 17.589 | 31.022 |
| ATOM597 | CZ | ARG | 41.042 | 17.964 | 30.337 |
| ATOM598 | NH1 | ARG | 41.878 | 18.723 | 29.67 |
| ATOM599 | HH11 | ARG | 41.712 | 19.72 | 29.677 |
| ATOM600 | HH12 | ARG | 42.81 | 18.444 | 29.398 |
| ATOM601 | NH2 | ARG | 41.394 | 16.707 | 30.423 |
| ATOM602 | HH21 | ARG | 40.773 | 15.918 | 30.539 |
| ATOM603 | HH22 | ARG | 42.352 | 16.428 | 30.27 |
| ATOM604 | C | ARG | 36.018 | 18.927 | 34.573 |
| ATOM605 | O | ARG | 36.613 | 19.084 | 35.637 |
| ATOM606 | N | GLY | 34.994 | 18.083 | 34.293 |
| ATOM607 | H | GLY | 34.791 | 17.949 | 33.312 |
| ATOM608 | CA | GLY | 34.431 | 17.151 | 35.297 |
| ATOM609 | HA2 | GLY | 33.606 | 16.612 | 34.831 |
| ATOM610 | HA3 | GLY | 35.19 | 16.383 | 35.445 |
| ATOM611 | C | GLY | 33.839 | 17.73 | 36.583 |
| ATOM612 | O | GLY | 34.046 | 17.251 | 37.667 |
| ATOM613 | N | GLY | 33.034 | 18.815 | 36.437 |
| ATOM614 | H | GLY | 32.905 | 19.143 | 35.49 |
| ATOM615 | CA | GLY | 32.272 | 19.367 | 37.513 |
| ATOM616 | HA2 | GLY | 31.686 | 18.606 | 38.029 |
| ATOM617 | HA3 | GLY | 32.957 | 19.837 | 38.219 |
| ATOM618 | C | GLY | 31.346 | 20.559 | 37.066 |
| ATOM619 | O | GLY | 31.456 | 21.013 | 35.934 |
| ATOM620 | N | PRO | 30.522 | 21.1 | 37.989 |
| ATOM621 | CD | PRO | 30.329 | 20.709 | 39.404 |
| ATOM622 | HD2 | PRO | 31.286 | 20.323 | 39.756 |
| ATOM623 | HD3 | PRO | 29.492 | 20.011 | 39.405 |
| ATOM624 | CG | PRO | 30.123 | 21.94 | 40.099 |
| ATOM625 | HG2 | PRO | 31.087 | 22.439 | 40.204 |
| ATOM626 | HG3 | PRO | 29.734 | 21.856 | 41.114 |
| ATOM627 | CB | PRO | 29.141 | 22.634 | 39.158 |
| ATOM628 | HB2 | PRO | 28.971 | 23.692 | 39.362 |
| ATOM629 | HB3 | PRO | 28.132 | 22.222 | 39.153 |
| ATOM630 | CA | PRO | 29.817 | 22.385 | 37.834 |
| ATOM631 | HA | PRO | 29.098 | 22.281 | 37.021 |
| ATOM632 | C | PRO | 30.724 | 23.615 | 37.411 |
| ATOM633 | O | PRO | 31.706 | 23.871 | 38.061 |
| ATOM634 | N | PRO | 30.292 | 24.486 | 36.435 |
| ATOM635 | CD | PRO | 29.125 | 24.328 | 35.619 |
| ATOM636 | HD2 | PRO | 28.272 | 24.197 | 36.285 |
| ATOM637 | HD3 | PRO | 29.313 | 23.423 | 35.042 |
| ATOM638 | CG | PRO | 29.021 | 25.501 | 34.735 |
| ATOM639 | HG2 | PRO | 28.213 | 26.167 | 35.037 |
| ATOM640 | HG3 | PRO | 28.823 | 25.162 | 33.718 |
| ATOM641 | CB | PRO | 30.365 | 26.265 | 34.823 |
| ATOM642 | HB2 | PRO | 30.224 | 27.343 | 34.909 |
| ATOM643 | HB3 | PRO | 31.028 | 25.923 | 34.028 |
| ATOM644 | CA | PRO | 30.984 | 25.707 | 36.019 |
| ATOM645 | HA | PRO | 32.006 | 25.463 | 35.73 |
| ATOM646 | C | PRO | 30.857 | 26.701 | 37.22 |
| ATOM647 | O | PRO | 30.087 | 26.593 | 38.191 |
| ATOM648 | N | PHE | 31.586 | 27.85 | 37.04 |
| ATOM649 | H | PHE | 32.186 | 27.969 | 36.236 |
| ATOM650 | CA | PHE | 31.506 | 29.087 | 37.924 |
| ATOM651 | HA | PHE | 30.533 | 29.142 | 38.413 |
| ATOM652 | CB | PHE | 32.507 | 29.036 | 39.097 |
| ATOM653 | HB2 | PHE | 32.297 | 29.786 | 39.86 |
| ATOM654 | HB3 | PHE | 32.51 | 28.05 | 39.562 |
| ATOM655 | CG | PHE | 33.964 | 29.123 | 38.702 |
| ATOM656 | CD1 | PHE | 34.633 | 28.031 | 38.023 |
| ATOM657 | HD1 | PHE | 34.032 | 27.208 | 37.664 |
| ATOM658 | CE1 | PHE | 36.009 | 28.181 | 37.695 |
| ATOM659 | HE1 | PHE | 36.504 | 27.409 | 37.126 |
| ATOM660 | CZ | PHE | 36.751 | 29.199 | 38.22 |
| ATOM661 | HZ | PHE | 37.793 | 29.26 | 37.941 |
| ATOM662 | CE2 | PHE | 36.101 | 30.256 | 38.9 |
| ATOM663 | HE2 | PHE | 36.643 | 31.182 | 39.03 |
| ATOM664 | CD2 | PHE | 34.73 | 30.232 | 39.168 |
| ATOM665 | HD2 | PHE | 34.303 | 31.044 | 39.738 |
| ATOM666 | C | PHE | 31.503 | 30.377 | 37.075 |
| ATOM667 | O | PHE | 32.055 | 30.468 | 35.98 |
| ATOM668 | N | ALA | 30.757 | 31.334 | 37.601 |
| ATOM669 | H | ALA | 30.111 | 31.183 | 38.363 |
| ATOM670 | CA | ALA | 30.773 | 32.702 | 37.043 |
| ATOM671 | HA | ALA | 31.583 | 32.878 | 36.335 |
| ATOM672 | CB | ALA | 29.419 | 32.925 | 36.343 |
| ATOM673 | HB1 | ALA | 29.244 | 33.86 | 35.81 |
| ATOM674 | HB2 | ALA | 29.307 | 32.13 | 35.606 |
| ATOM675 | HB3 | ALA | 28.593 | 32.885 | 37.052 |
| ATOM676 | C | ALA | 30.981 | 33.867 | 38.045 |
| ATOM677 | O | ALA | 30.611 | 33.833 | 39.199 |
| ATOM678 | N | PHE | 31.438 | 35.007 | 37.502 |
| ATOM679 | H | PHE | 31.526 | 34.992 | 36.496 |
| ATOM680 | CA | PHE | 31.711 | 36.258 | 38.148 |
| ATOM681 | HA | PHE | 31.59 | 36.029 | 39.207 |
| ATOM682 | CB | PHE | 33.251 | 36.681 | 37.981 |
| ATOM683 | HB2 | PHE | 33.415 | 36.955 | 36.938 |
| ATOM684 | HB3 | PHE | 33.388 | 37.591 | 38.565 |
| ATOM685 | CG | PHE | 34.25 | 35.704 | 38.593 |
| ATOM686 | CD1 | PHE | 35.106 | 34.902 | 37.851 |
| ATOM687 | HD1 | PHE | 35.019 | 34.892 | 36.775 |
| ATOM688 | CE1 | PHE | 36.228 | 34.267 | 38.467 |
| ATOM689 | HE1 | PHE | 36.804 | 33.588 | 37.855 |
| ATOM690 | CZ | PHE | 36.458 | 34.276 | 39.864 |
| ATOM691 | HZ | PHE | 37.209 | 33.623 | 40.284 |
| ATOM692 | CE2 | PHE | 35.558 | 35.012 | 40.633 |
| ATOM693 | HE2 | PHE | 35.68 | 35.089 | 41.703 |
| ATOM694 | CD2 | PHE | 34.498 | 35.643 | 40.017 |
| ATOM695 | HD2 | PHE | 33.898 | 36.292 | 40.638 |
| ATOM696 | C | PHE | 30.716 | 37.353 | 37.66 |
| ATOM697 | O | PHE | 30.32 | 37.33 | 36.507 |
| ATOM698 | N | VAL | 30.291 | 38.218 | 38.554 |
| ATOM699 | H | VAL | 30.691 | 38.06 | 39.468 |
| ATOM700 | CA | VAL | 29.257 | 39.216 | 38.441 |
| ATOM701 | HA | VAL | 28.993 | 39.399 | 37.4 |
| ATOM702 | CB | VAL | 27.818 | 38.774 | 38.998 |
| ATOM703 | HB | VAL | 27.894 | 38.608 | 40.073 |
| ATOM704 | CG1 | VAL | 26.708 | 39.784 | 38.744 |
| ATOM705 | HG11 | VAL | 27.053 | 40.78 | 39.02 |
| ATOM706 | HG12 | VAL | 26.399 | 39.781 | 37.699 |
| ATOM707 | HG13 | VAL | 25.855 | 39.459 | 39.34 |
| ATOM708 | CG2 | VAL | 27.373 | 37.438 | 38.366 |
| ATOM709 | HG21 | VAL | 27.315 | 37.515 | 37.28 |
| ATOM710 | HG22 | VAL | 27.98 | 36.592 | 38.69 |
| ATOM711 | HG23 | VAL | 26.345 | 37.344 | 38.718 |
| ATOM712 | C | VAL | 29.689 | 40.507 | 39.099 |
| ATOM713 | O | VAL | 30.082 | 40.565 | 40.262 |
| ATOM714 | N | GLU | 29.587 | 41.606 | 38.303 |
| ATOM715 | H | GLU | 29.164 | 41.387 | 37.413 |
| ATOM716 | CA | GLU | 29.806 | 42.992 | 38.628 |
| ATOM717 | HA | GLU | 30.059 | 43.141 | 39.677 |
| ATOM718 | CB | GLU | 30.944 | 43.574 | 37.719 |
| ATOM719 | HB2 | GLU | 31.603 | 42.715 | 37.593 |
| ATOM720 | HB3 | GLU | 30.427 | 43.766 | 36.779 |
| ATOM721 | CG | GLU | 31.515 | 44.873 | 38.343 |
| ATOM722 | HG2 | GLU | 32.193 | 45.241 | 37.573 |
| ATOM723 | HG3 | GLU | 30.739 | 45.635 | 38.421 |
| ATOM724 | CD | GLU | 32.329 | 44.734 | 39.628 |
| ATOM725 | OE1 | GLU | 33.545 | 44.877 | 39.567 |
| ATOM726 | OE2 | GLU | 31.885 | 44.167 | 40.641 |
| ATOM727 | C | GLU | 28.526 | 43.78 | 38.398 |
| ATOM728 | O | GLU | 27.898 | 43.708 | 37.323 |
| ATOM729 | N | PHE | 28.247 | 44.718 | 39.292 |
| ATOM730 | H | PHE | 28.856 | 44.778 | 40.096 |
| ATOM731 | CA | PHE | 27.175 | 45.652 | 39.435 |
| ATOM732 | HA | PHE | 26.539 | 45.53 | 38.558 |
| ATOM733 | CB | PHE | 26.225 | 45.342 | 40.604 |
| ATOM734 | HB2 | PHE | 26.795 | 45.456 | 41.526 |
| ATOM735 | HB3 | PHE | 25.546 | 46.195 | 40.588 |
| ATOM736 | CG | PHE | 25.447 | 44.062 | 40.605 |
| ATOM737 | CD1 | PHE | 24.092 | 43.997 | 40.302 |
| ATOM738 | HD1 | PHE | 23.646 | 44.961 | 40.111 |
| ATOM739 | CE1 | PHE | 23.403 | 42.773 | 40.427 |
| ATOM740 | HE1 | PHE | 22.336 | 42.822 | 40.272 |
| ATOM741 | CZ | PHE | 24.116 | 41.602 | 40.852 |
| ATOM742 | HZ | PHE | 23.546 | 40.688 | 40.772 |
| ATOM743 | CE2 | PHE | 25.455 | 41.683 | 41.24 |
| ATOM744 | HE2 | PHE | 25.889 | 40.742 | 41.546 |
| ATOM745 | CD2 | PHE | 26.071 | 42.98 | 41.197 |
| ATOM746 | HD2 | PHE | 27.102 | 43.088 | 41.501 |
| ATOM747 | C | PHE | 27.664 | 47.128 | 39.376 |
| ATOM748 | O | PHE | 28.725 | 47.511 | 39.86 |
| ATOM749 | N | GLU | 26.735 | 47.997 | 38.942 |
| ATOM750 | H | GLU | 25.916 | 47.524 | 38.589 |
| ATOM751 | CA | GLU | 26.881 | 49.396 | 38.822 |
| ATOM752 | HA | GLU | 27.869 | 49.617 | 38.418 |
| ATOM753 | CB | GLU | 25.796 | 50.002 | 37.884 |
| ATOM754 | HB2 | GLU | 24.795 | 49.742 | 38.226 |
| ATOM755 | HB3 | GLU | 25.876 | 51.089 | 37.903 |
| ATOM756 | CG | GLU | 25.853 | 49.487 | 36.429 |
| ATOM757 | HG2 | GLU | 25.91 | 48.418 | 36.222 |
| ATOM758 | HG3 | GLU | 25.003 | 49.903 | 35.888 |
| ATOM759 | CD | GLU | 26.989 | 50.095 | 35.578 |
| ATOM760 | OE1 | GLU | 26.903 | 50.124 | 34.37 |
| ATOM761 | OE2 | GLU | 27.995 | 50.536 | 36.178 |
| ATOM762 | C | GLU | 26.813 | 50.073 | 40.217 |
| ATOM763 | O | GLU | 27.14 | 51.236 | 40.381 |
| ATOM764 | N | ASP | 26.497 | 49.283 | 41.24 |
| ATOM765 | H | ASP | 26.125 | 48.369 | 41.025 |
| ATOM766 | CA | ASP | 26.512 | 49.755 | 42.639 |
| ATOM767 | HA | ASP | 27.107 | 50.661 | 42.755 |
| ATOM768 | CB | ASP | 25.053 | 50.193 | 42.958 |
| ATOM769 | HB2 | ASP | 24.725 | 50.798 | 42.112 |
| ATOM770 | HB3 | ASP | 24.366 | 49.349 | 42.896 |
| ATOM771 | CG | ASP | 24.717 | 50.748 | 44.377 |
| ATOM772 | OD1 | ASP | 25.598 | 50.721 | 45.254 |
| ATOM773 | OD2 | ASP | 23.625 | 51.268 | 44.619 |
| ATOM774 | C | ASP | 26.95 | 48.586 | 43.509 |
| ATOM775 | O | ASP | 26.275 | 47.566 | 43.572 |
| ATOM776 | N | PRO | 28.112 | 48.681 | 44.172 |
| ATOM777 | CD | PRO | 28.925 | 49.898 | 44.265 |
| ATOM778 | HD2 | PRO | 28.312 | 50.798 | 44.292 |
| ATOM779 | HD3 | PRO | 29.529 | 49.991 | 43.362 |
| ATOM780 | CG | PRO | 29.632 | 49.841 | 45.579 |
| ATOM781 | HG2 | PRO | 29.094 | 50.19 | 46.46 |
| ATOM782 | HG3 | PRO | 30.613 | 50.314 | 45.619 |
| ATOM783 | CB | PRO | 29.787 | 48.327 | 45.777 |
| ATOM784 | HB2 | PRO | 29.805 | 48.099 | 46.842 |
| ATOM785 | HB3 | PRO | 30.646 | 47.927 | 45.239 |
| ATOM786 | CA | PRO | 28.53 | 47.697 | 45.11 |
| ATOM787 | HA | PRO | 28.918 | 46.789 | 44.649 |
| ATOM788 | C | PRO | 27.53 | 47.281 | 46.199 |
| ATOM789 | O | PRO | 27.666 | 46.228 | 46.781 |
| ATOM790 | N | ARG | 26.554 | 48.141 | 46.493 |
| ATOM791 | H | ARG | 26.483 | 48.977 | 45.932 |
| ATOM792 | CA | ARG | 25.533 | 47.894 | 47.5 |
| ATOM793 | HA | ARG | 25.919 | 47.51 | 48.444 |
| ATOM794 | CB | ARG | 24.87 | 49.206 | 48.044 |
| ATOM795 | HB2 | ARG | 24.242 | 49.612 | 47.251 |
| ATOM796 | HB3 | ARG | 24.17 | 48.896 | 48.82 |
| ATOM797 | CG | ARG | 25.917 | 50.23 | 48.464 |
| ATOM798 | HG2 | ARG | 26.362 | 49.749 | 49.334 |
| ATOM799 | HG3 | ARG | 26.741 | 50.492 | 47.8 |
| ATOM800 | CD | ARG | 25.257 | 51.626 | 48.703 |
| ATOM801 | HD2 | ARG | 24.436 | 51.569 | 49.417 |
| ATOM802 | HD3 | ARG | 26.017 | 52.292 | 49.112 |
| ATOM803 | NE | ARG | 24.673 | 52.166 | 47.472 |
| ATOM804 | HE | ARG | 25.039 | 51.641 | 46.691 |
| ATOM805 | CZ | ARG | 23.844 | 53.134 | 47.461 |
| ATOM806 | NH1 | ARG | 23.607 | 54.011 | 48.411 |
| ATOM807 | HH11 | ARG | 24.088 | 53.852 | 49.284 |
| ATOM808 | HH12 | ARG | 22.799 | 54.615 | 48.347 |
| ATOM809 | NH2 | ARG | 23.282 | 53.327 | 46.315 |
| ATOM810 | HH21 | ARG | 23.488 | 52.624 | 45.619 |
| ATOM811 | HH22 | ARG | 22.498 | 53.941 | 46.139 |
| ATOM812 | C | ARG | 24.408 | 47.017 | 46.933 |
| ATOM813 | O | ARG | 23.83 | 46.218 | 47.678 |
| ATOM814 | N | ASP | 24.19 | 47.071 | 45.627 |
| ATOM815 | H | ASP | 24.669 | 47.741 | 45.042 |
| ATOM816 | CA | ASP | 23.408 | 46.079 | 44.859 |
| ATOM817 | HA | ASP | 22.442 | 45.929 | 45.341 |
| ATOM818 | CB | ASP | 23.114 | 46.622 | 43.434 |
| ATOM819 | HB2 | ASP | 23.979 | 46.979 | 42.874 |
| ATOM820 | HB3 | ASP | 22.708 | 45.812 | 42.828 |
| ATOM821 | CG | ASP | 22.121 | 47.82 | 43.459 |
| ATOM822 | OD1 | ASP | 21.354 | 48.143 | 44.384 |
| ATOM823 | OD2 | ASP | 22.039 | 48.609 | 42.443 |
| ATOM824 | C | ASP | 24.108 | 44.771 | 44.803 |
| ATOM825 | O | ASP | 23.399 | 43.77 | 44.993 |
| ATOM826 | N | ALA | 25.422 | 44.648 | 44.638 |
| ATOM827 | H | ALA | 25.888 | 45.49 | 44.332 |
| ATOM828 | CA | ALA | 26.208 | 43.431 | 44.831 |
| ATOM829 | HA | ALA | 25.895 | 42.767 | 44.025 |
| ATOM830 | CB | ALA | 27.658 | 43.756 | 44.541 |
| ATOM831 | HB1 | ALA | 28.23 | 44.264 | 45.317 |
| ATOM832 | HB2 | ALA | 28.206 | 42.88 | 44.192 |
| ATOM833 | HB3 | ALA | 27.728 | 44.407 | 43.67 |
| ATOM834 | C | ALA | 26.012 | 42.761 | 46.227 |
| ATOM835 | O | ALA | 25.795 | 41.571 | 46.349 |
| ATOM836 | N | GLU | 26.103 | 43.551 | 47.257 |
| ATOM837 | H | GLU | 26.449 | 44.497 | 47.173 |
| ATOM838 | CA | GLU | 25.735 | 43.033 | 48.59 |
| ATOM839 | HA | GLU | 26.308 | 42.146 | 48.861 |
| ATOM840 | CB | GLU | 26.046 | 44.109 | 49.599 |
| ATOM841 | HB2 | GLU | 27.092 | 44.416 | 49.614 |
| ATOM842 | HB3 | GLU | 25.371 | 44.934 | 49.372 |
| ATOM843 | CG | GLU | 25.647 | 43.542 | 51.054 |
| ATOM844 | HG2 | GLU | 24.607 | 43.276 | 51.237 |
| ATOM845 | HG3 | GLU | 26.293 | 42.674 | 51.184 |
| ATOM846 | CD | GLU | 26.266 | 44.489 | 52.037 |
| ATOM847 | OE1 | GLU | 25.565 | 44.907 | 53.006 |
| ATOM848 | OE2 | GLU | 27.44 | 44.929 | 51.957 |
| ATOM849 | C | GLU | 24.299 | 42.511 | 48.713 |
| ATOM850 | O | GLU | 24.156 | 41.327 | 49.077 |
| ATOM851 | N | ASP | 23.226 | 43.253 | 48.344 |
| ATOM852 | H | ASP | 23.327 | 44.103 | 47.808 |
| ATOM853 | CA | ASP | 21.805 | 42.699 | 48.338 |
| ATOM854 | HA | ASP | 21.513 | 42.306 | 49.312 |
| ATOM855 | CB | ASP | 20.895 | 43.832 | 47.9 |
| ATOM856 | HB2 | ASP | 21.108 | 44.114 | 46.869 |
| ATOM857 | HB3 | ASP | 19.897 | 43.403 | 47.811 |
| ATOM858 | CG | ASP | 20.779 | 45.097 | 48.688 |
| ATOM859 | OD1 | ASP | 20.928 | 45.129 | 49.947 |
| ATOM860 | OD2 | ASP | 20.343 | 46.114 | 48.065 |
| ATOM861 | C | ASP | 21.695 | 41.457 | 47.433 |
| ATOM862 | O | ASP | 20.957 | 40.567 | 47.758 |
| ATOM863 | N | ALA | 22.523 | 41.309 | 46.411 |
| ATOM864 | H | ALA | 22.995 | 42.142 | 46.09 |
| ATOM865 | CA | ALA | 22.625 | 40.137 | 45.626 |
| ATOM866 | HA | ALA | 21.611 | 39.952 | 45.272 |
| ATOM867 | CB | ALA | 23.421 | 40.383 | 44.366 |
| ATOM868 | HB1 | ALA | 23.099 | 41.243 | 43.779 |
| ATOM869 | HB2 | ALA | 24.435 | 40.645 | 44.67 |
| ATOM870 | HB3 | ALA | 23.461 | 39.502 | 43.724 |
| ATOM871 | C | ALA | 23.227 | 38.944 | 46.461 |
| ATOM872 | O | ALA | 22.698 | 37.848 | 46.333 |
| ATOM873 | N | VAL | 24.373 | 39.121 | 47.106 |
| ATOM874 | H | VAL | 24.676 | 40.084 | 47.128 |
| ATOM875 | CA | VAL | 24.99 | 38.055 | 47.89 |
| ATOM876 | HA | VAL | 25.14 | 37.274 | 47.145 |
| ATOM877 | CB | VAL | 26.328 | 38.429 | 48.475 |
| ATOM878 | HB | VAL | 26.144 | 39.363 | 49.006 |
| ATOM879 | CG1 | VAL | 27.018 | 37.46 | 49.527 |
| ATOM880 | HG11 | VAL | 27.172 | 36.455 | 49.134 |
| ATOM881 | HG12 | VAL | 27.92 | 38.003 | 49.809 |
| ATOM882 | HG13 | VAL | 26.412 | 37.267 | 50.412 |
| ATOM883 | CG2 | VAL | 27.332 | 38.745 | 47.343 |
| ATOM884 | HG21 | VAL | 26.99 | 39.557 | 46.701 |
| ATOM885 | HG22 | VAL | 28.346 | 38.981 | 47.665 |
| ATOM886 | HG23 | VAL | 27.491 | 37.809 | 46.807 |
| ATOM887 | C | VAL | 24.104 | 37.474 | 48.96 |
| ATOM888 | O | VAL | 23.968 | 36.242 | 49.055 |
| ATOM889 | N | TYR | 23.471 | 38.395 | 49.663 |
| ATOM890 | H | TYR | 23.739 | 39.362 | 49.554 |
| ATOM891 | CA | TYR | 22.491 | 38.125 | 50.758 |
| ATOM892 | HA | TYR | 22.834 | 37.247 | 51.305 |
| ATOM893 | CB | TYR | 22.216 | 39.331 | 51.672 |
| ATOM894 | HB2 | TYR | 22.055 | 40.258 | 51.122 |
| ATOM895 | HB3 | TYR | 21.273 | 39.247 | 52.212 |
| ATOM896 | CG | TYR | 23.418 | 39.497 | 52.578 |
| ATOM897 | CD1 | TYR | 23.632 | 38.625 | 53.671 |
| ATOM898 | HD1 | TYR | 22.938 | 37.858 | 53.982 |
| ATOM899 | CE1 | TYR | 24.74 | 38.814 | 54.497 |
| ATOM900 | HE1 | TYR | 24.902 | 38.183 | 55.358 |
| ATOM901 | CZ | TYR | 25.789 | 39.707 | 54.157 |
| ATOM902 | OH | TYR | 26.827 | 39.947 | 54.975 |
| ATOM903 | HH | TYR | 27.213 | 40.784 | 54.706 |
| ATOM904 | CE2 | TYR | 25.59 | 40.567 | 53.043 |
| ATOM905 | HE2 | TYR | 26.274 | 41.364 | 52.79 |
| ATOM906 | CD2 | TYR | 24.428 | 40.428 | 52.277 |
| ATOM907 | HD2 | TYR | 24.154 | 41.037 | 51.428 |
| ATOM908 | C | TYR | 21.148 | 37.515 | 50.177 |
| ATOM909 | O | TYR | 20.268 | 37.049 | 50.885 |
| ATOM910 | N | GLY | 21.038 | 37.694 | 48.846 |
| ATOM911 | H | GLY | 21.789 | 38.179 | 48.377 |
| ATOM912 | CA | GLY | 19.78 | 37.369 | 48.076 |
| ATOM913 | HA2 | GLY | 18.933 | 37.543 | 48.739 |
| ATOM914 | HA3 | GLY | 19.69 | 38.017 | 47.205 |
| ATOM915 | C | GLY | 19.853 | 35.865 | 47.569 |
| ATOM916 | O | GLY | 18.938 | 35.125 | 47.849 |
| ATOM917 | N | ARG | 20.89 | 35.507 | 46.779 |
| ATOM918 | H | ARG | 21.577 | 36.242 | 46.694 |
| ATOM919 | CA | ARG | 21.127 | 34.319 | 45.986 |
| ATOM920 | HA | ARG | 20.162 | 33.822 | 45.883 |
| ATOM921 | CB | ARG | 21.616 | 34.642 | 44.585 |
| ATOM922 | HB2 | ARG | 22.553 | 35.189 | 44.685 |
| ATOM923 | HB3 | ARG | 21.727 | 33.711 | 44.03 |
| ATOM924 | CG | ARG | 20.598 | 35.368 | 43.752 |
| ATOM925 | HG2 | ARG | 20.307 | 36.368 | 44.073 |
| ATOM926 | HG3 | ARG | 21.105 | 35.554 | 42.805 |
| ATOM927 | CD | ARG | 19.288 | 34.544 | 43.592 |
| ATOM928 | HD2 | ARG | 19.464 | 33.525 | 43.246 |
| ATOM929 | HD3 | ARG | 18.707 | 34.544 | 44.514 |
| ATOM930 | NE | ARG | 18.367 | 35.172 | 42.525 |
| ATOM931 | HE | ARG | 18.798 | 35.606 | 41.72 |
| ATOM932 | CZ | ARG | 17.043 | 35.146 | 42.478 |
| ATOM933 | NH1 | ARG | 16.426 | 34.746 | 43.533 |
| ATOM934 | HH11 | ARG | 16.896 | 34.217 | 44.254 |
| ATOM935 | HH12 | ARG | 15.418 | 34.687 | 43.487 |
| ATOM936 | NH2 | ARG | 16.392 | 35.482 | 41.394 |
| ATOM937 | HH21 | ARG | 16.808 | 35.585 | 40.48 |
| ATOM938 | HH22 | ARG | 15.405 | 35.63 | 41.549 |
| ATOM939 | C | ARG | 21.96 | 33.219 | 46.603 |
| ATOM940 | O | ARG | 21.999 | 32.127 | 46.058 |
| ATOM941 | N | ASP | 22.611 | 33.396 | 47.771 |
| ATOM942 | H | ASP | 22.457 | 34.256 | 48.277 |
| ATOM943 | CA | ASP | 23.436 | 32.384 | 48.356 |
| ATOM944 | HA | ASP | 24.142 | 32.072 | 47.586 |
| ATOM945 | CB | ASP | 24.238 | 32.919 | 49.509 |
| ATOM946 | HB2 | ASP | 24.848 | 33.752 | 49.159 |
| ATOM947 | HB3 | ASP | 23.528 | 33.167 | 50.299 |
| ATOM948 | CG | ASP | 25.241 | 31.873 | 49.903 |
| ATOM949 | OD1 | ASP | 25.894 | 32.029 | 50.965 |
| ATOM950 | OD2 | ASP | 25.524 | 30.927 | 49.156 |
| ATOM951 | C | ASP | 22.619 | 31.156 | 48.758 |
| ATOM952 | O | ASP | 22.046 | 31.078 | 49.897 |
| ATOM953 | N | GLY | 22.547 | 30.161 | 47.914 |
| ATOM954 | H | GLY | 23.201 | 30.173 | 47.145 |
| ATOM955 | CA | GLY | 21.749 | 28.984 | 48.161 |
| ATOM956 | HA2 | GLY | 22.379 | 28.172 | 47.799 |
| ATOM957 | HA3 | GLY | 21.513 | 28.753 | 49.2 |
| ATOM958 | C | GLY | 20.403 | 29.051 | 47.466 |
| ATOM959 | O | GLY | 19.526 | 28.311 | 47.806 |
| ATOM960 | N | TYR | 20.211 | 29.916 | 46.472 |
| ATOM961 | H | TYR | 21.024 | 30.478 | 46.268 |
| ATOM962 | CA | TYR | 19.004 | 29.841 | 45.63 |
| ATOM963 | HA | TYR | 18.104 | 29.859 | 46.245 |
| ATOM964 | CB | TYR | 19.163 | 31.01 | 44.649 |
| ATOM965 | HB2 | TYR | 19.189 | 31.939 | 45.218 |
| ATOM966 | HB3 | TYR | 20.14 | 30.829 | 44.201 |
| ATOM967 | CG | TYR | 18.143 | 31.174 | 43.541 |
| ATOM968 | CD1 | TYR | 16.789 | 31.308 | 43.823 |
| ATOM969 | HD1 | TYR | 16.509 | 31.311 | 44.865 |
| ATOM970 | CE1 | TYR | 15.842 | 31.509 | 42.733 |
| ATOM971 | HE1 | TYR | 14.77 | 31.531 | 42.866 |
| ATOM972 | CZ | TYR | 16.303 | 31.575 | 41.397 |
| ATOM973 | OH | TYR | 15.493 | 31.841 | 40.362 |
| ATOM974 | HH | TYR | 14.608 | 32.064 | 40.658 |
| ATOM975 | CE2 | TYR | 17.676 | 31.402 | 41.155 |
| ATOM976 | HE2 | TYR | 18.067 | 31.498 | 40.152 |
| ATOM977 | CD2 | TYR | 18.571 | 31.202 | 42.2 |
| ATOM978 | HD2 | TYR | 19.616 | 31.083 | 41.959 |
| ATOM979 | C | TYR | 18.946 | 28.526 | 44.858 |
| ATOM980 | O | TYR | 20.01 | 28.209 | 44.28 |
| ATOM981 | N | ASP | 17.807 | 27.775 | 44.807 |
| ATOM982 | H | ASP | 16.946 | 28.09 | 45.231 |
| ATOM983 | CA | ASP | 17.658 | 26.562 | 44.043 |
| ATOM984 | HA | ASP | 18.529 | 25.908 | 44.084 |
| ATOM985 | CB | ASP | 16.527 | 25.622 | 44.51 |
| ATOM986 | HB2 | ASP | 15.534 | 26.032 | 44.327 |
| ATOM987 | HB3 | ASP | 16.467 | 24.748 | 43.861 |
| ATOM988 | CG | ASP | 16.682 | 25.13 | 45.929 |
| ATOM989 | OD1 | ASP | 17.799 | 24.83 | 46.343 |
| ATOM990 | OD2 | ASP | 15.622 | 25.028 | 46.627 |
| ATOM991 | C | ASP | 17.383 | 26.914 | 42.575 |
| ATOM992 | O | ASP | 16.311 | 27.462 | 42.282 |
| ATOM993 | N | TYR | 18.216 | 26.605 | 41.597 |
| ATOM994 | H | TYR | 18.937 | 25.931 | 41.809 |
| ATOM995 | CA | TYR | 18.201 | 27.003 | 40.149 |
| ATOM996 | HA | TYR | 17.196 | 27.312 | 39.86 |
| ATOM997 | CB | TYR | 19.189 | 28.192 | 40.008 |
| ATOM998 | HB2 | TYR | 18.971 | 29.079 | 40.603 |
| ATOM999 | HB3 | TYR | 20.178 | 27.867 | 40.332 |
| ATOM1000 | CG | TYR | 19.16 | 28.694 | 38.584 |
| ATOM1001 | CD1 | TYR | 20.286 | 28.553 | 37.774 |
| ATOM1002 | HD1 | TYR | 21.136 | 27.994 | 38.137 |
| ATOM1003 | CE1 | TYR | 20.225 | 28.986 | 36.364 |
| ATOM1004 | HE1 | TYR | 21.065 | 28.87 | 35.695 |
| ATOM1005 | CZ | TYR | 19.024 | 29.489 | 35.834 |
| ATOM1006 | OH | TYR | 18.9 | 29.833 | 34.482 |
| ATOM1007 | HH | TYR | 19.528 | 29.26 | 34.037 |
| ATOM1008 | CE2 | TYR | 17.977 | 29.704 | 36.736 |
| ATOM1009 | HE2 | TYR | 17.022 | 29.947 | 36.293 |
| ATOM1010 | CD2 | TYR | 17.996 | 29.377 | 38.107 |
| ATOM1011 | HD2 | TYR | 17.144 | 29.559 | 38.745 |
| ATOM1012 | C | TYR | 18.478 | 25.807 | 39.266 |
| ATOM1013 | O | TYR | 19.535 | 25.28 | 39.375 |
| ATOM1014 | N | ASP | 17.65 | 25.495 | 38.292 |
| ATOM1015 | H | ASP | 16.778 | 26.005 | 38.279 |
| ATOM1016 | CA | ASP | 17.621 | 24.259 | 37.423 |
| ATOM1017 | HA | ASP | 16.617 | 24.191 | 37.004 |
| ATOM1018 | CB | ASP | 18.578 | 24.507 | 36.261 |
| ATOM1019 | HB2 | ASP | 18.59 | 25.561 | 35.983 |
| ATOM1020 | HB3 | ASP | 19.632 | 24.357 | 36.496 |
| ATOM1021 | CG | ASP | 18.295 | 23.577 | 34.982 |
| ATOM1022 | OD1 | ASP | 17.246 | 22.927 | 34.831 |
| ATOM1023 | OD2 | ASP | 19.121 | 23.384 | 34.083 |
| ATOM1024 | C | ASP | 17.854 | 22.915 | 38.143 |
| ATOM1025 | O | ASP | 18.65 | 22.032 | 37.651 |
| ATOM1026 | N | GLY | 17.254 | 22.73 | 39.335 |
| ATOM1027 | H | GLY | 16.708 | 23.516 | 39.658 |
| ATOM1028 | CA | GLY | 17.485 | 21.57 | 40.199 |
| ATOM1029 | HA2 | GLY | 16.617 | 21.394 | 40.835 |
| ATOM1030 | HA3 | GLY | 17.661 | 20.699 | 39.569 |
| ATOM1031 | C | GLY | 18.786 | 21.701 | 41.055 |
| ATOM1032 | O | GLY | 19.101 | 20.834 | 41.891 |
| ATOM1033 | N | TYR | 19.669 | 22.643 | 40.751 |
| ATOM1034 | H | TYR | 19.483 | 23.37 | 40.075 |
| ATOM1035 | CA | TYR | 21.007 | 22.831 | 41.485 |
| ATOM1036 | HA | TYR | 21.335 | 21.921 | 41.988 |
| ATOM1037 | CB | TYR | 22.156 | 23.187 | 40.545 |
| ATOM1038 | HB2 | TYR | 21.913 | 24.114 | 40.026 |
| ATOM1039 | HB3 | TYR | 23.159 | 23.286 | 40.961 |
| ATOM1040 | CG | TYR | 22.308 | 22.069 | 39.53 |
| ATOM1041 | CD1 | TYR | 23.009 | 20.908 | 39.965 |
| ATOM1042 | HD1 | TYR | 23.47 | 20.815 | 40.937 |
| ATOM1043 | CE1 | TYR | 23.155 | 19.868 | 39.041 |
| ATOM1044 | HE1 | TYR | 23.643 | 18.975 | 39.401 |
| ATOM1045 | CZ | TYR | 22.667 | 19.955 | 37.669 |
| ATOM1046 | OH | TYR | 22.963 | 18.928 | 36.855 |
| ATOM1047 | HH | TYR | 22.737 | 19.115 | 35.942 |
| ATOM1048 | CE2 | TYR | 22.01 | 21.143 | 37.2 |
| ATOM1049 | HE2 | TYR | 21.656 | 21.12 | 36.179 |
| ATOM1050 | CD2 | TYR | 21.848 | 22.218 | 38.173 |
| ATOM1051 | HD2 | TYR | 21.278 | 23.121 | 38.01 |
| ATOM1052 | C | TYR | 20.76 | 23.905 | 42.606 |
| ATOM1053 | O | TYR | 19.757 | 24.548 | 42.735 |
| ATOM1054 | N | ARG | 21.775 | 24.175 | 43.429 |
| ATOM1055 | H | ARG | 22.577 | 23.605 | 43.2 |
| ATOM1056 | CA | ARG | 21.69 | 25.256 | 44.498 |
| ATOM1057 | HA | ARG | 20.828 | 25.906 | 44.346 |
| ATOM1058 | CB | ARG | 21.316 | 24.633 | 45.921 |
| ATOM1059 | HB2 | ARG | 20.452 | 23.969 | 45.903 |
| ATOM1060 | HB3 | ARG | 22.207 | 24.029 | 46.093 |
| ATOM1061 | CG | ARG | 21.028 | 25.574 | 47.103 |
| ATOM1062 | HG2 | ARG | 21.927 | 26.125 | 47.38 |
| ATOM1063 | HG3 | ARG | 20.218 | 26.219 | 46.762 |
| ATOM1064 | CD | ARG | 20.558 | 24.621 | 48.297 |
| ATOM1065 | HD2 | ARG | 19.791 | 23.881 | 48.066 |
| ATOM1066 | HD3 | ARG | 21.433 | 24.031 | 48.572 |
| ATOM1067 | NE | ARG | 20.179 | 25.383 | 49.481 |
| ATOM1068 | HE | ARG | 20.888 | 25.648 | 50.149 |
| ATOM1069 | CZ | ARG | 18.98 | 25.87 | 49.709 |
| ATOM1070 | NH1 | ARG | 17.946 | 25.689 | 49.002 |
| ATOM1071 | HH11 | ARG | 18.023 | 25.149 | 48.152 |
| ATOM1072 | HH12 | ARG | 17.061 | 26.165 | 49.1 |
| ATOM1073 | NH2 | ARG | 18.754 | 26.664 | 50.713 |
| ATOM1074 | HH21 | ARG | 19.522 | 26.774 | 51.36 |
| ATOM1075 | HH22 | ARG | 17.837 | 27.032 | 50.924 |
| ATOM1076 | C | ARG | 22.88 | 26.151 | 44.558 |
| ATOM1077 | O | ARG | 23.986 | 25.707 | 44.781 |
| ATOM1078 | N | LEU | 22.725 | 27.449 | 44.259 |
| ATOM1079 | H | LEU | 21.816 | 27.86 | 44.1 |
| ATOM1080 | CA | LEU | 23.888 | 28.345 | 44.059 |
| ATOM1081 | HA | LEU | 24.548 | 27.856 | 43.343 |
| ATOM1082 | CB | LEU | 23.456 | 29.68 | 43.449 |
| ATOM1083 | HB2 | LEU | 22.762 | 30.061 | 44.199 |
| ATOM1084 | HB3 | LEU | 24.266 | 30.406 | 43.509 |
| ATOM1085 | CG | LEU | 22.814 | 29.742 | 42.135 |
| ATOM1086 | HG | LEU | 21.848 | 29.241 | 42.196 |
| ATOM1087 | CD1 | LEU | 22.515 | 31.151 | 41.684 |
| ATOM1088 | HD11 | LEU | 23.407 | 31.631 | 41.281 |
| ATOM1089 | HD12 | LEU | 21.768 | 31 | 40.905 |
| ATOM1090 | HD13 | LEU | 22.07 | 31.77 | 42.463 |
| ATOM1091 | CD2 | LEU | 23.63 | 29.061 | 41.09 |
| ATOM1092 | HD21 | LEU | 23.659 | 27.992 | 41.299 |
| ATOM1093 | HD22 | LEU | 23.251 | 29.175 | 40.074 |
| ATOM1094 | HD23 | LEU | 24.639 | 29.467 | 41.169 |
| ATOM1095 | C | LEU | 24.756 | 28.426 | 45.347 |
| ATOM1096 | O | LEU | 24.215 | 28.288 | 46.468 |
| ATOM1097 | N | ARG | 26.061 | 28.698 | 45.236 |
| ATOM1098 | H | ARG | 26.525 | 28.744 | 44.341 |
| ATOM1099 | CA | ARG | 26.874 | 29.15 | 46.372 |
| ATOM1100 | HA | ARG | 26.158 | 29.176 | 47.194 |
| ATOM1101 | CB | ARG | 27.999 | 28.144 | 46.626 |
| ATOM1102 | HB2 | ARG | 27.809 | 27.086 | 46.81 |
| ATOM1103 | HB3 | ARG | 28.625 | 28.005 | 45.744 |
| ATOM1104 | CG | ARG | 28.981 | 28.611 | 47.781 |
| ATOM1105 | HG2 | ARG | 29.87 | 27.981 | 47.798 |
| ATOM1106 | HG3 | ARG | 29.443 | 29.567 | 47.534 |
| ATOM1107 | CD | ARG | 28.406 | 28.611 | 49.186 |
| ATOM1108 | HD2 | ARG | 27.446 | 28.114 | 49.054 |
| ATOM1109 | HD3 | ARG | 29.022 | 27.899 | 49.736 |
| ATOM1110 | NE | ARG | 28.171 | 29.905 | 49.827 |
| ATOM1111 | HE | ARG | 27.211 | 30.129 | 50.047 |
| ATOM1112 | CZ | ARG | 29.162 | 30.684 | 50.21 |
| ATOM1113 | NH1 | ARG | 30.38 | 30.357 | 49.902 |
| ATOM1114 | HH11 | ARG | 30.499 | 29.438 | 49.499 |
| ATOM1115 | HH12 | ARG | 31.263 | 30.836 | 50.003 |
| ATOM1116 | NH2 | ARG | 28.916 | 31.723 | 50.936 |
| ATOM1117 | HH21 | ARG | 27.971 | 32.026 | 51.126 |
| ATOM1118 | HH22 | ARG | 29.701 | 32.116 | 51.435 |
| ATOM1119 | C | ARG | 27.439 | 30.56 | 45.979 |
| ATOM1120 | O | ARG | 28.093 | 30.516 | 44.948 |
| ATOM1121 | N | VAL | 27.104 | 31.598 | 46.711 |
| ATOM1122 | H | VAL | 26.522 | 31.403 | 47.514 |
| ATOM1123 | CA | VAL | 27.219 | 32.985 | 46.131 |
| ATOM1124 | HA | VAL | 27.786 | 32.906 | 45.204 |
| ATOM1125 | CB | VAL | 25.788 | 33.541 | 45.764 |
| ATOM1126 | HB | VAL | 25.223 | 33.649 | 46.689 |
| ATOM1127 | CG1 | VAL | 25.864 | 34.835 | 44.962 |
| ATOM1128 | HG11 | VAL | 24.856 | 35.222 | 44.817 |
| ATOM1129 | HG12 | VAL | 26.308 | 35.562 | 45.642 |
| ATOM1130 | HG13 | VAL | 26.44 | 34.675 | 44.05 |
| ATOM1131 | CG2 | VAL | 25.131 | 32.466 | 44.946 |
| ATOM1132 | HG21 | VAL | 24.218 | 32.9 | 44.538 |
| ATOM1133 | HG22 | VAL | 25.797 | 32.055 | 44.187 |
| ATOM1134 | HG23 | VAL | 24.826 | 31.713 | 45.672 |
| ATOM1135 | C | VAL | 27.908 | 33.825 | 47.15 |
| ATOM1136 | O | VAL | 27.464 | 33.9 | 48.308 |
| ATOM1137 | N | GLU | 29.111 | 34.352 | 46.873 |
| ATOM1138 | H | GLU | 29.579 | 34.375 | 45.978 |
| ATOM1139 | CA | GLU | 29.943 | 34.979 | 47.887 |
| ATOM1140 | HA | GLU | 29.231 | 35.469 | 48.551 |
| ATOM1141 | CB | GLU | 30.707 | 33.983 | 48.665 |
| ATOM1142 | HB2 | GLU | 31.14 | 34.333 | 49.602 |
| ATOM1143 | HB3 | GLU | 30.146 | 33.148 | 49.087 |
| ATOM1144 | CG | GLU | 31.917 | 33.346 | 47.941 |
| ATOM1145 | HG2 | GLU | 31.515 | 32.758 | 47.116 |
| ATOM1146 | HG3 | GLU | 32.661 | 34.055 | 47.581 |
| ATOM1147 | CD | GLU | 32.76 | 32.454 | 48.801 |
| ATOM1148 | OE1 | GLU | 32.514 | 32.186 | 50.033 |
| ATOM1149 | OE2 | GLU | 33.732 | 31.911 | 48.204 |
| ATOM1150 | C | GLU | 30.876 | 36.11 | 47.317 |
| ATOM1151 | O | GLU | 31.069 | 36.292 | 46.139 |
| ATOM1152 | N | PHE | 31.326 | 36.923 | 48.23 |
| ATOM1153 | H | PHE | 31.063 | 36.862 | 49.203 |
| ATOM1154 | CA | PHE | 32.434 | 37.837 | 47.895 |
| ATOM1155 | HA | PHE | 32.289 | 38.338 | 46.938 |
| ATOM1156 | CB | PHE | 32.43 | 38.905 | 48.994 |
| ATOM1157 | HB2 | PHE | 32.425 | 38.478 | 49.997 |
| ATOM1158 | HB3 | PHE | 33.299 | 39.563 | 48.967 |
| ATOM1159 | CG | PHE | 31.296 | 39.912 | 48.805 |
| ATOM1160 | CD1 | PHE | 30.291 | 39.998 | 49.766 |
| ATOM1161 | HD1 | PHE | 30.262 | 39.21 | 50.505 |
| ATOM1162 | CE1 | PHE | 29.284 | 40.966 | 49.752 |
| ATOM1163 | HE1 | PHE | 28.593 | 40.985 | 50.582 |
| ATOM1164 | CZ | PHE | 29.369 | 41.895 | 48.681 |
| ATOM1165 | HZ | PHE | 28.553 | 42.594 | 48.57 |
| ATOM1166 | CE2 | PHE | 30.345 | 41.84 | 47.711 |
| ATOM1167 | HE2 | PHE | 30.266 | 42.46 | 46.831 |
| ATOM1168 | CD2 | PHE | 31.392 | 40.921 | 47.853 |
| ATOM1169 | HD2 | PHE | 32.179 | 40.887 | 47.113 |
| ATOM1170 | C | PHE | 33.701 | 36.906 | 47.908 |
| ATOM1171 | O | PHE | 33.961 | 36.32 | 48.963 |
| ATOM1172 | N | PRO | 34.609 | 36.938 | 46.913 |
| ATOM1173 | CD | PRO | 34.437 | 37.679 | 45.686 |
| ATOM1174 | HD2 | PRO | 34.242 | 38.739 | 45.848 |
| ATOM1175 | HD3 | PRO | 33.61 | 37.265 | 45.109 |
| ATOM1176 | CG | PRO | 35.695 | 37.454 | 44.842 |
| ATOM1177 | HG2 | PRO | 36.455 | 38.191 | 45.1 |
| ATOM1178 | HG3 | PRO | 35.374 | 37.574 | 43.807 |
| ATOM1179 | CB | PRO | 36.082 | 36.029 | 45.329 |
| ATOM1180 | HB2 | PRO | 37.142 | 35.864 | 45.141 |
| ATOM1181 | HB3 | PRO | 35.429 | 35.352 | 44.778 |
| ATOM1182 | CA | PRO | 35.764 | 36.04 | 46.842 |
| ATOM1183 | HA | PRO | 35.517 | 34.987 | 46.973 |
| ATOM1184 | C | PRO | 36.913 | 36.411 | 47.839 |
| ATOM1185 | O | PRO | 37.2 | 37.53 | 48.15 |
| ATOM1186 | N | ARG | 37.57 | 35.376 | 48.308 |
| ATOM1187 | H | ARG | 37.215 | 34.482 | 48.002 |
| ATOM1188 | CA | ARG | 38.858 | 35.369 | 48.996 |
| ATOM1189 | HA | ARG | 39.067 | 36.332 | 49.462 |
| ATOM1190 | CB | ARG | 38.769 | 34.472 | 50.231 |
| ATOM1191 | HB2 | ARG | 38.517 | 33.477 | 49.864 |
| ATOM1192 | HB3 | ARG | 39.759 | 34.465 | 50.687 |
| ATOM1193 | CG | ARG | 37.618 | 34.922 | 51.193 |
| ATOM1194 | HG2 | ARG | 36.674 | 35.067 | 50.667 |
| ATOM1195 | HG3 | ARG | 37.352 | 34.132 | 51.894 |
| ATOM1196 | CD | ARG | 38.036 | 36.218 | 52.033 |
| ATOM1197 | HD2 | ARG | 38.907 | 36.104 | 52.679 |
| ATOM1198 | HD3 | ARG | 38.314 | 37.043 | 51.377 |
| ATOM1199 | NE | ARG | 36.891 | 36.759 | 52.856 |
| ATOM1200 | HE | ARG | 36.561 | 36.243 | 53.66 |
| ATOM1201 | CZ | ARG | 36.145 | 37.769 | 52.51 |
| ATOM1202 | NH1 | ARG | 36.31 | 38.505 | 51.387 |
| ATOM1203 | HH11 | ARG | 37.119 | 38.34 | 50.805 |
| ATOM1204 | HH12 | ARG | 35.757 | 39.345 | 51.293 |
| ATOM1205 | NH2 | ARG | 35.136 | 38.164 | 53.186 |
| ATOM1206 | HH21 | ARG | 34.852 | 37.54 | 53.928 |
| ATOM1207 | HH22 | ARG | 34.528 | 38.902 | 52.86 |
| ATOM1208 | C | ARG | 39.98 | 35.063 | 48.034 |
| ATOM1209 | O | ARG | 40.477 | 33.906 | 47.886 |
| ATOM1210 | OXT | ARG | 40.391 | 36.026 | 47.303 |

**Table S6.** The list of 50 candidate SRSF1 inhibitor screened in vitro.

| tID | tName | docking score | Formula | MolWt | Pathways | Receptor | Target |
| --- | --- | --- | --- | --- | --- | --- | --- |
| T1517 | Benserazide hydrochloride | -6.3166776 | C10H15N3O5HCl | 293.70001 | GPCR/G Protein; Neuroscience | DOPA decarboxylase; Dopamine | Dopamine Receptor inhibitor |
| T4613 | Arg-Gly-Asp TFA (99896-85-2(free base)) | -5.7156186 | C14H23F3N6O8 | 460.35999 | Cytoskeletal Signaling | Integrin | Integrin |
| T1638 | N-Acetylneuraminic acid | -5.6001134 | C11H19NO9 | 309.26999 | Others | Others | Others |
| T19412 | Macrozamin | -4.9649568 | C13H24N2O11 | 384.34 | Others | Others | Others |
| T4706 | UDP disodium salt | -5.3865013 | C9H12N2Na2O12P2 | 466.14001 | Neuroscience | P2Y6; P2Y14 | P2 Receptor |
| T7969 | Dipyrithione | -5.3899646 | C10H8N2O2S2 | 252.32001 | Others | Others | Others |
| T0771 | Casanthranol | -5.2179589 | C21H22O10 | 396.57999 | Others | Others | Others |
| TQ0104 | CRT0066101 dihydrochloride | -5.0290542 | C18H22N6O.2HCl | 411.32999 | Apoptosis | PKD1; PKD2; PKD3 | Serine/threonin kinase |
| TN2008 | Okanin | -4.7493348 | C15H12O6 | 288.29999 | Immunology/Inflammation;NF-Kb | TLR4; NF-KB | TLR; NF-KB |
| T0257 | Mepiroxol | -5.0430369 | C6H7NO2 | 125.13 | Others | Others | Others |
| T0067 | Nicotinic acid N-oxide | -4.9151311 | C6H5NO3 | 139.11 | Others | Others | Others |
| T0877 | Gallic acid | -4.9059796 | C7H6O5 | 170.12 | Immunology/Inflammation | ROS | ROS |
| T6S0721 | Orientin | -4.836132 | C21H20O11 | 448.38 | Others | Others | Others |
| T2987 | Epigallocatechin | -4.8519936 | C15H14O7 | 306.26999 | Metabolic Enzyme/Protease | MMP | MMP |
| T1170 | Olopatadine hydrochloride | -4.8548131 | C21H23NO3HCl | 373.87 | GPCR/G Protein; Neuroscience | HT | 5-HT Receptor antagonist |
| T5065 | GUANOSINE 3':5'-CYCLIC MONOPHOSPHATE SOD | -4.8527784 | C10H11N5NaO7P | 367.19 | GPCR/G Protein | PKG | PKA |
| T0771 | Casanthranol | -4.8264265 | C21H22O10 | 396.57999 | Others | Others | Others |
| 8014-8811 |  | -4.7792258 | C10H12N4O6 |  |  |  |  |
| T4718 | Uridine 5'-monophosphate | -4.6681561 | C9H13N2O9P | 324.17999 | Others | Others | Others |
| T2366 | Tipiracil hydrochloride | -4.5710559 | C9H12Cl2N4O2 | 279.12 | DNA Damage/DNA Repair | Nucleoside Antimetabolite/Analog | Nucleoside Antimetabolite/Analog inhibitor |
| T6S1657 | Paeonolide | -4.7273345 | C20H28O12 | 460.42999 | Others | Others | Others |
| T5S1645 | Apiopaeonoside | -4.6594887 | C20H28O12 | 460.42999 | Others | Others | Others |
| T3834 | 8-Epideoxyloganic acid | -4.6342936 | C16H24O9 | 360.39999 | Immunology/Inflammation | Immunology & Inflammation related;ROS | Immunology & Inflammation related;ROS |
| T4185 | lavendustin C | -4.6113734 | C14H13NO5 | 275.26001 | Proteases/Proteasome | tyrosine kinase | Tyrosinase inhibitor |
| T1738 | Taxifolin | -4.5722079 | C15H12O7 | 304.25 | GPCR/G Protein; Angiogenesis; Apoptosis; Proteases/Proteasome; Tyrosine Kinase/Adaptors | ??-adrenergic receptor; TNF-??; Collagenase Tyrosinase; Beta-nerve growth factor; VEGFR2 | Adrenergic Receptor antagonist; TNF inhibitor; Tyrosinase inhibitor; VEGFR inhibitor |
| T22459 | Xanthinol Nicotinate | -4.3752022 | C13H21N5O4.C6H5NO2 | 434.45001 | Others | Others | Others |
| T0970 | Anisodamine | -4.4432316 | C17H23NO4 | 305.38 | Neuroscience; GPCR/G Protein | Adrenergic Receptor | Adrenergic Receptor inhibitor |
| T0739 | Guaifenesin | -4.4180942 | C10H14O4 | 198.22 | Others | Others | Others |
| T8358 | Fenoterol | -4.3918085 | C17H21NO4 | 303.35001 | GPCR/G Protein | ??2-adrenergic receptor | Adrenergic Receptor |
| TQ0208 | SAH | -4.3872671 | C14H17N6O5S | 384.41 | Others | Others | Others |
| T7571 | 1-Methyladenosine | -4.319849 | C11H15N5O4 | 281.26999 | Metabolic Enzyme/Protease | Endogenous Metabolite | Endogenous Metabolite |
| T1037 | Doripenem Hydrate | -3.9008746 | C15H24N4O6S2??H2O | 438.51999 | Microbiology&Virology | Antibiotic | Antibiotic |
| 7649-0007 |  | -3.9831612 | C22H28N2O4 |  |  |  |  |
| T0158 | Mitoxantrone hydrochloride | -4.0713549 | C22H30Cl2N4O6 | 517.40002 | DNA Damage/DNA Repair | Topo II | Topoisomerase inhibitor |
| 3836-0073 |  | -4.0410786 | C15H17N3O4 |  |  |  |  |
| T3232 | HigenamineHydrochloride | -3.9327462 | C16H17NO3??HCl | 307.76999 | Neuroscience | ??2-adrenergic receptor | Adrenergic Receptor |
| T6652 | Salbutamol Sulfate | -4.0431881 | C13H21NO3??H2SO4 | 337.39001 | Neuroscience;GPCR/G Protein | ??2-adrenergic receptor | Adrenergic Receptor agonist |
| T6448 | Clindamycin HCl | -3.9781594 | C18H33ClN2O5S??HCl | 461.44 | Microbiology&Virology | Antibiotic | Antibiotic |
| T1643 | Penciclovir | -3.9505467 | C10H15N5O3 | 253.25999 | Microbiology&Virology; Proteases/Proteasome | EBV; HCMV; HSV-1; HSV-2; VZV | Antifection inhibitor; HCV Protease inhibitor; HSV inhibitor |
| T0688 | Ganciclovir | -3.9409869 | C9H13N5O4 | 255.23 | Microbiology&Virology | FHV-1 | Antifection inhibitor |
| T4006 | Pentostatin | -3.8454115 | C11H16N4O4 | 268.26999 | Neuroscience | Adenosine receptor | AChR inhibitor |
| T7203 | Oglufanide | -3.7872519 | C16H19N3O5 | 333.34 | Angiogenesis;Tyrosine Kinase/Adaptors | VEGFR | VEGFR |
| TN1051 | 1-Caffeoylquinic acid | -3.8483174 | C16H18O9 | 354.31 | NF-kB | NF-kB | NF-kB |
| T1203 | Nadolol | -3.760489 | C17H27NO4 | 309.39999 | GPCR/G Protein | ??-adrenergic receptor | Adrenergic Receptor antagonist |
| T4063 | Delafloxacin | -3.6389544 | C18H12ClF3N4O4 | 440.76001 | Microbiology&Virology | Bacterial | Antibacterial inhibitor |
| T0964 | Floxuridine | -3.6409581 | C9H11FN2O5 | 246.19 | DNA Damage/DNA Repair | Thymidylate synthase | DNA/RNA Synthesis inhibitor |
| T5755 | Aloesin | -3.6133518 | C19H22O9 | 394.37 | Proteases/Proteasome | Tyrosinase | Tyrosinase |
| TN1406 | Aromadendrin | -3.5772419 | C15H12O6 | 288.29999 | Apoptosis | Bcl-2;Bcl-xL | BCL |
| T6884 | 2-Cl-IB-MECA | -3.4749081 | C18H18ClIN6O4 | 544.72998 | GPCR/G Protein | A3 | Adenosine Receptor |
| T0085L | Entecavir | -3.4398305 | C12H15N5O3 | 277.28 | Microbiology&Virology | HBV | HBV inhibitor |

| **Table S7.** List of primers sequences and shRNA sequences used in this research. | |
| --- | --- |
| **Real-time qPCR primers sequences** | **Sequence (5'→3')** |
| SRSF1 F | GGAAGACGCGGTGTATGGTC |
| SRSF1 R | CACCTGCTTCACGCATGTG |
| mSrsf1 F | GGTCCGAGAACAGAGTGGTT |
| mSrsf1 R | AGGCAGTTTCTCCCTCGTGA |
| β-actin F | GGGAAATCGTGCGTGACATTAAG |
| β-actin R | TGTGTTGGCGTACAGGTCTTTG |
| LDHA-F | CAAAGACTACTGTGTAACTGCGA |
| LDHA-R | TGGACTGTACTTGACAATGTTGG |
| PGK1-F | ATGTCGCTTTCCAACAAGCTG |
| PGK1-R | GCTCCATTGTCCAAGCAGAAT |
| PGAM1-F | AGCGACACTATGGCGGTCT |
| PGAM1-R | TGGGACATCATAAGATCGTCTCC |
| mItgae F | GTTCCGCAGCCTCAACTCAGAG |
| mItgae R | GCCATCTTCCTCCTCGTCTTCCT |
| mGzma F | TCATTGGAGGAGACACGGTTGTTC |
| mGzma R | CGTTACAGTGGGCAGCAGTCAA |
| mIrf7 F | GTCACCACACTACACCATCTACCT |
| mIrf7 R | AGACAAGCACAAGCCGAGACTG |
| mIfng F | CATGGCTGTTTCTGGCTGTTACTG |
| mIfng R | TGACGCTTATGTTGTTGCTGATGG |
| mNlk F | TGGGCAACAACAGCCATATTT |
| mNlk R | GTGCGCCTTAACTGTAGCAG |
|  |  |
| **RIP-PCR-primers** |  |
| c-JUN_RIP_F | TTCCTCCAGTCCGAGAGCG |
| c-JUN_RIP_R | TGAGAAGGTCCGAGTTCTTGG |
| JUNB_RIP_F | TCACGACGACTCTTACGCAG |
| JUNB_RIP_R | CCTTGAGACCCCGATAGGGA |
| c-MYC_RIP_F | ATGCCCCTCAACGTGAACTTC |
| c-MYC_RIP_R | GTCGCAGATGAAATAGGGCTG |
|  |  |
| **RT-PCR-primers** |  |
| c-JUN_F | CCTTCTACGACGATGCCCTC |
| c-JUN_R | GGTTCAAGGTCATGCTCTGTTT |
| JUNB_F | TCACGACGACTCTTACGCAG |
| JUNB_R | CCTTGAGACCCCGATAGGGA |
| c-MYC_F (post-spliced) | TGGAACTTACAACACCCG |
| c-MYC_R (post-spliced) | CCTCGTCGCAGTAGAAAT |
| c-MYC_F (pre-spliced) | CCTTCTCTCCGTCCTCGGAT |
| c-MYC_R (pre-spliced) | TATCCAGCCGCCCACTTTTG |
|  |  |
| **ChIP assays primers sequences** |  |
| Nfatc2-Srsf1 site F | ACCTGTGGGGATCCGACTTA |
| Nfatc2-Srsf1 site R | TTCCTGCCTTCTGAGTGCTG |

**Table S8.** The list of antibodies used in CyTOF assays.

| **List** | **Label** | **marker** | **clone** | **dilution** | **Extracellular** |  |
| --- | --- | --- | --- | --- | --- | --- |
| 1 | 89Y | CD45 | 30-F11 | 200 | Biolegend |  |
| 2 | 115In | CD3e | 145-2C11 | 50 | Biolegend |  |
| 3 | 139La | Ki-67 | SolA15 | 100 | eB | intra |
| 4 | 141Pr | CD117-c-kit | 2B8 | 200 | Biolegend |  |
| 5 | 142Nd | MHC II | M5-114.15.2 | 800 | Biolegend |  |
| 6 | 143Nd | CD45R-B220 | RA3-6B2 | 100 | Biolegend |  |
| 7 | 144Nd | CX3CR1 | SA011F11 | 800 | Biolegend |  |
| 8 | 145Nd | CD163 | S15049I | 200 | Biolegend |  |
| 9 | 146Nd | CD38 | 90 | 200 | Biolegend |  |
| 10 | 147Sm | Ly6G | IA8 | 800 | Biolegend |  |
| 11 | 148Nd | Ly-6C | HK1.4 | 400 | Biolegend |  |
| 12 | 149Sm | CD19 | 6D5 | 400 | Biolegend |  |
| 13 | 150Nd | CD127-IL-7Ra | A7R34 | 100 | Biolegend |  |
| 14 | 151Eu | CD62L | MEL-14 | 800 | Biolegend |  |
| 15 | 152Sm | CD11c | N418 | 200 | Biolegend |  |
| 16 | 153Eu | CD44 | IM7 | 400 | Biolegend |  |
| 17 | 154Sm | CD24 | M1/69 | 200 | Biolegend |  |
| 18 | 155Gd | CD103 | 2E7 | 200 | Biolegend |  |
| 19 | 156Gd | Gr-1-Ly6G-C | RB6-8C5 | 400 | Biolegend |  |
| 20 | 157Gd | FceRIa | MAR-1 | 100 | Biolegend |  |
| 21 | 158Gd | TCRgd | GL3 | 200 | Biolegend |  |
| 22 | 159Tb | F4-80 | C1:A3-1 | 100 | Biorad |  |
| 23 | 160Gd | TCRb | H57-597 | 100 | Biolegend |  |
| 24 | 161Dy | CD64-FcrRI | X54-5-7.1 | 50 | Biolegend |  |
| 25 | 162Dy | CD69 | H1.2F3 | 400 | Biolegend |  |
| 26 | 163Dy | CD25-IL-2R | 3C7 | 100 | Biolegend |  |
| 27 | 164Dy | CD86 | GL-1 | 200 | Biolegend |  |
| 28 | 165Ho | CD161c-NK1.1 | PK136 | 100 | Biolegend |  |
| 29 | 166Er | CD27 | LG.3A10 | 100 | Biolegend |  |
| 30 | 167Er | CD206 | C068C2 | 50 | Biolegend | intra |
| 31 | 168Er | FoxP3 | FJK-16s | 50 | eB | intra |
| 32 | 169Tm | CD317-BST2 | 44E9R | 800 | RD |  |
| 33 | 170Er | IgD | 11-26c.2a | 100 | Biolegend |  |
| 34 | 171Yb | CD80 | 16-10A1 | 200 | Biolegend |  |
| 35 | 172Yb | CD279-PD1 | 29F.1A12 | 200 | Biolegend |  |
| 36 | 173Yb | CD172a | P84 | 100 | Biolegend |  |
| 37 | 174Yb | CD192-CCR2 | 475301 | 100 | RD |  |
| 38 | 175Lu | SiglecF | E50-2440 | 100 | BD |  |
| 39 | 176Yb | MertK | 2B10C42 | 100 | Biolegend |  |
| 40 | 197Au | CD4 | RM4-5 | 400 | Biolegend |  |
| 41 | 198Pt | CD8 | 53-6.7 | 200 | Biolegend |  |
| 42 | 209Bi | CD11b | M1-70 | 400 | Biolegend |  |
